# Speaker Abstracts

**DOI:** 10.1002/jia2.25263

**Published:** 2019-04-02

## O11 – Keynote Lectures

## O111

### Keynote Lecture: The safety of antiretroviral drugs during the first trimester of pregnancy

**L Mofenson**

Elizabeth Glaser Pediatric AIDS Foundation, Washington, DC, USA

There are only limited data on antiretroviral drugs (ARVs) in pregnancy. Of the thirty‐two currently approved ARVs, six lack any data in pregnancy, including four of the nine drugs approved by the FDA since 2011. Most ARVs receive approval with only animal preclinical data to evaluate potential fetal effects; for those ARVs that are studied in pregnancy, the mean lag time between drug approval and pregnancy study is >4 years. Exposure timing is critical in terms of safety, as teratogenic risk is highest very early in pregnancy, before most women recognise they are pregnant. To determine if a birth defect is associated with a drug or simply reflects the baseline population rate of a defect, the number of required exposures will vary based on the defect population prevalence. With 200 early pregnancy exposures, a twofold increase in overall birth defects with population prevalence 3% can be ruled out; however, for lower prevalence defects like neural tube defects, with 0.05% prevalence in countries with folic acid food fortification and 0.10% without fortification, a minimum of 2000 early exposures are needed to rule out a threefold increase. Data on birth defects with ARV exposure will be discussed. In addition to birth defects, some data suggest that preconception use of ARVs may be associated with adverse pregnancy outcome such as preterm delivery, low birthweight or stillbirth, and that there may be differences between ARV regimens in such effects; examples will be discussed.

## O112

### Keynote Lecture: Treatment and prevention in adolescents and their transition to adult care

**A Agwu**

Pediatric and Adult Infectious Diseases, Johns Hopkins University of School of Medicine, Baltimore, MD, USA

With greater than 50% of new HIV infections occurring in adolescent and young adults worldwide and increasing numbers of children surviving into adolescence, there is a critical need to specifically consider this key population in HIV prevention and treatment strategies. There are unique developmental, cognitive, biologic, psychosocial and societal factors that contribute to the challenges encountered in optimising care and outcomes for adolescents and young adults. This population has lower rates of diagnosis, engagement and ultimately viral suppression, which threaten to derail the gains in HIV treatment and care for this population. Furthermore, our systems of care, often fragmented for this evolving population, can worsen outcomes. This whirlwind lecture will discuss the worldwide landscape of adolescents, review the current epidemiology of HIV in this population, assess the factors that contribute to risk and management challenges, analyse systems of care and their impact on outcomes (including transition to adult care), highlight promising models and interventions, and areas of need for research to optimise outcomes for this key, growing population.

## O12 – HIV in HPV

## O121

### Keynote Lecture: HPV in HIV: state‐of‐the‐art for clinicians

**T Wilkin**

Weill Cornell Medicine, New York, NY, USA

Human papillomavirus (HPV)‐related cancers, including cervical, anal and oropharyngeal cancer, occur more frequently in individuals living with HIV infection than in the general population. Strategies for prevention among individuals with HIV infection include HPV vaccination, cervical cancer and anal cancer screening programmes, and early initiation of antiretroviral therapy (ART). HPV vaccination is not yet optimally used as a preventive vaccine; a stronger and more persistent effort is needed to increase vaccination rates and combat anti‐vaccine stigma. Much of Latin America relies on cytology‐based methods for cervical cancer screening; however, primary HPV‐based screening is gaining acceptance worldwide. Although anal cancer screening is not recommended by all authorities, there is at least some evidence that screening and treatment of anal high‐grade squamous intraepithelial lesions may prevent progression to cancer. However, more definitive evidence is needed. More data is needed on the epidemiology of HPV‐related oropharyngeal cancer in Latin America and prevention modalities are urgently needed. Early initiation of ART reduces the risk of infection‐related cancers, with some evidence of benefit in preventing HPV‐associated cancer in individuals with HIV infection.

## O122

### Current anal cancer screening practices in HIV‐positive MSM population: a single centre, 10‐year follow‐up study in Mexico City

**R Ville‐Benavides^1^, Y Caro‐Vega^1^, B Delgado‐Avila^1^, J Sierra‐Madero^1^, A García‐Carrancá^2^ and B Crabtree‐Ramírez^1^**

^1^Infectious Diseases, Instituto Nacional de Ciencias Médicas y Nutrición Salvador Zubirán, Mexico City, Mexico. ^2^Biología Molecular y Biotecnología, Instituto de Investigaciones Biomédicas, Mexico City, Mexico

**Introduction**

In Mexico, the HIV epidemic is concentrated in men who have sex with men (MSM), in whom anal HPV infection (HPVI) ranges from 70% to 100% [1]. This population is considered to be at the highest risk for developing anal cancer [2]. Therefore, screening is recommended, but there is a lack of local guidelines on this regard [3]. In Mexico, although universal access to free ART has been available since 2002, anal cytologies (AC), biopsies and anorectal specialist consultations expenditures are covered by patients. The aim of this study was to evaluate the frequency and results of anal cancer screening practices at our centre during a 10‐year period.

**Methods**

We performed a 10‐year follow‐up observational study of HIV‐infected MSM, who were screened for anal HPVI at baseline (year 2008). We retrospectively analysed all AC and biopsies performed from 2008 to 2018, and described their frequency and results. In 2013, a prospective questionnaire to address sexually transmitted diseases including HPVI was implemented. The frequency of anal cancer screening was described in two groups: those with high‐risk HPV (HR‐HPV) (16 and/or 18 serotypes) versus those with any other HPV serotype at baseline.

**Results**

Of 326 patients, 279 (85%) were infected with HPV, 94 (33%) were HR‐HPV. At 10‐year follow‐up, 298 (85%) were retained in HIV care. Three hundred and five AC were performed (median per patient 1.0, IQR: 0 to 2). 99% were performed during and after 2013. At 10 years follow‐up, 65% of patients without HR‐HPV had at least one AC versus 57% of patients with HR‐HPV. 43% AC were benign, 26% had a low‐grade lesion, 2 (0.5%) had a high‐grade lesion (HGL), 3% had atypical cells of undetermined significance (ASC‐US) and 21 (7%) were inappropriate. Of 33 anal biopsies, 15 (55%) had HR‐HPV; 18 (54%) were benign, 5 (15%) had grade 1 anal intraepithelial neoplasia (AIN), 4 (12%) had grade 3 AIN and 6 (18%) had *in situ* carcinoma. At baseline, HR‐HPV serotypes were found in three of those who developed cancer (75%) and in one of HGLs (50%). Only two of the four anal cancer and two HGL patients had a previous AC performed in 10 years of follow‐up. None of the ASC‐US were prospectively biopsied.

**Conclusions**

We found that anal cancer screening is suboptimal even in HR‐HPV and HGL individuals. Adding a prospective questionnaire increased practices of anal cancer screening, considerably. This supports that implementing awareness strategies is effective, and reinforces the need for public funding to improve anal cancer screening practices as part of routine care in HIV patients.

**References**

1. Lin C, Franceschi S, Clifford M. Human papillomavirus types from infection to cancer in the anus, according to sex and HIV status: a systematic review and meta‐analysis. Lancet Infect Dis. 2018;18(2):198–206.

2. de Pokomandy A, et al. HAART and progression to high‐grade anal intraepithelial neoplasia in men who have sex with men and are infected with HIV. Clin Infect Dis. 2011;52(9):1174–181.

3. Gosens KC, Richel O, Prins JM. Human papillomavirus as a cause of anal cancer and the role of screening. Curr Opin Infect Dis. 2017;30(1):87–92.

## O13 – Keynote Lecture

## O131

### Keynote Lecture: First‐line therapy in the region

**P Cahn**

Fundación Huésped, Buenos Aires, Argentina

Latin America and the Caribbean (LAC) is a region with a wide heterogeneity and fragmentation at the regional and local level. Access to care is unequal, and this, in the HIV field has a direct impact on people's chances to receive appropriate counselling and access to a combination prevention package, which includes timely testing and access to ARV therapy. Albeit *in paper* access to testing and treatment is free in all LAC countries, in real life key populations are frequently set aside due to a complex array of factors, including poverty, social injustice, stigma, discrimination, migration, bad governance, inefficiency and corruption. Most (but not all) countries in the region have adopted the World Health Organization recommendation regarding the immediate universal offer of HAART initiation. Uptake of integrase inhibitors in first‐line is slow and limited to a reduced number of countries. While access to plasma viral load testing has expanded, access to baseline resistance testing is still limited to a few countries. Efavirenz is still used as first‐line anchor drug, notwithstanding the increasing resistance rate to NNRTIs. Cost of medicines consumes at least one third of the national health budget in most countries. The Pan American Health Organization Strategic Fund is a regional mechanism for pooled procurement of medicines, including antiretrovirals, but a minority of countries take advantage of this tool. Apart from Argentina, Brazil, Chile, Colombia, Mexico and Uruguay, HIV programmes focused on key populations are too dependent on donor funding, which threatens their sustainability. The region has made good progress but we still have a long way to go.

## O132

Abstract withdrawn

## O21 – Keynote Lectures and GHESKIO Oral Papers

## O211

### Keynote Lecture: Challenges of TB/HIV in the region

**A Piñeirúa‐Menendez**

Clinica Especializada Condesa Iztapalapa, Mexico City, Mexico

Worldwide, tuberculosis (TB) remains the leading cause of mortality among people living with HIV (PLWH); accounting for about one third of deaths. TB incidence and outcomes are extremely related to other development determinants such as expenditure per capita, sanitation facilities and life expectancy at birth. According to UNAIDS, by 2016 TB was responsible for 4800 deaths in the Latin American region and 1000 deaths in the Caribbean. From the clinical perspective, many challenges remain in the TB/HIV field; including screening and management of latent tuberculosis infection (LTBI), atypical clinical presentations, multidrug resistant TB, duration of anti‐TB treatment in PLWH, concomitant use of antiretroviral therapy (ART) with anti‐TB agents and immune reconstitution inflammatory syndrome related to TB. Recent evidence has proven that the use of preventive isoniazid as treatment for LTBI has an effect on mortality among PLWH, a similar effect has been observed in studies assessing early ART initiation after TB diagnosis and treatment. Another major issue has been the co‐administration of anti‐TB treatment and ART in low‐ and middle‐income countries with restricted access to rifabutin and integrase inhibitors. Efavirenz is still the first option in this group of patients, although available data show that raltegravir and twice daily dolutegravir might represent a safe and effective option for this population. Advocacy to strengthen health policies and programmes to improve availability of adequate treatments and diagnostic tools are undoubtedly needed in our region in order to tackle this important public health challenge.

## O212

### Substantial declines in HIV, syphilis, genital ulcers and urethral discharge over two decades, with recent increases in sexually transmitted infection (STI) prevalence in an STI clinic in urban Haiti

**B Liautaud^1^, S Koenig^2^, M Greenberg^3^, E Serra^3^, G Hilaire^1^, A Marcelin^1^, J Bernadin^1^, M Macheca^3^ and J Pape^1^**

^1^Medicine, GHESKIO, Port‐au‐Prince, Haiti. ^2^Medicine, Brigham and Women's Hospital, Boston, MA, USA. ^3^Analysis, Analysis Group, Boston, MA, USA

**Purpose**

A retrospective study was conducted to describe the prevalence of HIV, syphilis, genital ulcers and urethral discharge among patients presenting to a sexually transmitted infections STI clinic in urban Haiti over the last two decades.

**Methods**

Patients who presented to the GHESKIO STI clinic in Port‐au‐Prince, Haiti from 1995 to 2015 were included. At initial entry into the STI clinic, HIV and syphilis testing were offered to all patients. Patients were tested for HIV with two rapid tests, in accordance with World Health Organization guidelines. They were classified as testing positive for syphilis if they had a positive treponemal or non‐treponemal test.

**Results**

A total of 102,724 (81,913 female, 20,811 male) visits were included in the study, with 782 (498 female, 284 male) visits occurring in 1995, and 11,293 (9060 female, 2233 male) occurring in 2015. HIV prevalence declined over time (*p* < 0.001). In females, the proportion of patients testing positive for HIV declined from 19% in 1995 to 5% in 2015, and in males, it declined from 32% to 6% over this time period. Syphilis prevalence declined from 1995 to 2012, and then increased in both females and males from 2012 to 2015. In females, the proportion of patients testing positive for syphilis decreased from 11% in 1995 to 3% in 2012, and then increased to 8% in 2015 (*p* < 0.001). In males, it decreased from 29% in 1995 to 5% in 2012, and then increased to 13% in 2015 (*p* < 0.001). Genital ulcer disease declined throughout the study period, from 19% in males and 3% in females in 1995 to 0.8% in males and 0.2% in females in 2015 (*p* < 0.001). The prevalence of urethral discharge decreased in males from 22% in 1995 to 18% in 2012, but then increased to 57% in 2015 (*p* = 0.04) (Figure 1).

**Conclusion**

The proportion of patients testing positive for HIV, syphilis, genital ulcer disease and urethral discharge at presentation to an STI clinic in urban Haiti decreased from 1995 to 2012. We attribute this decline to the scale‐up in access to comprehensive HIV and STI prevention and treatment services, and massive national information programmes about safer sexual practices. However, the proportion of patients testing positive for syphilis and urethral discharge (in males) has increased in recent years, coincident with an increasing economic crisis in Haiti. Additional efforts are needed to prevent a resurgence of STIs and HIV in Haiti.

Abstract O212‐Figure 1. Male patient diagnosis trends, as a percentage of all male patients, 1995 to 2015.

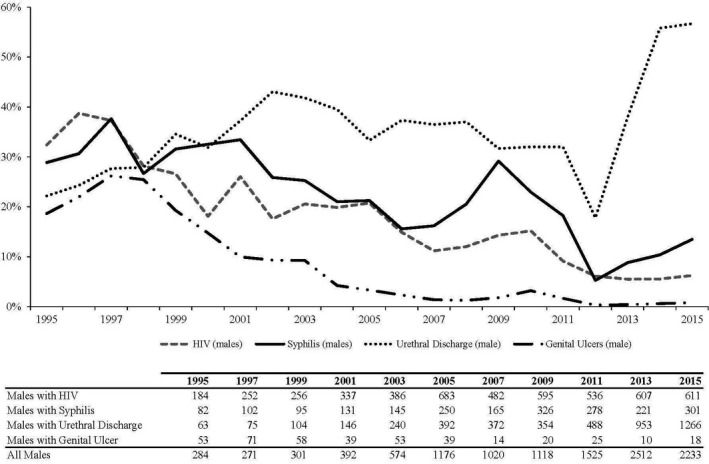

## O213

### Factors associated with disengagement from HIV care at GHESKIO, main HIV centre in the Caribbean

**M Deschamps, C Bertil, A Marcelin, Y Macius and J Pape**

Global Health, Centres GHESKIO, Port‐au‐Prince, Haiti

**Objective**

To define the intrinsic factors associated with lost to follow‐up of HIV‐positive patients in order to address the issues and achieve significant reductions in patient disengagement.

**Methods**

Retrospective review of data collected as part of routine clinical care was performed from HIV‐positive patients tested at GHESKIO from January 2014 to October 2018. Data on patients are recorded in an electronic database by doctors and nurses. Patient's information is recorded at each visit into a file which is entered into the computer system. Patients retained in care were defined as those actively receiving antiretroviral (ART) at the GHESKIO clinic and patients defined as lost follow‐up (LTFU) are those not returning for HIV services for at least one month after their last expected appointment date during study period. Data of patients who remained in care and received ART were compared with those who did not return for HIV services to determine characteristics associated with LTFU. Abstracted data included, gender, age, education level, income, sociodemographic profile, ART treatment, last CD4 count and viral load. Chi‐square analyses were used to determine predictors of LTFU. Using a threshold of *p* < 0.1, we conducted a multivariate regression analysis to determine independent predictors of LTFU.

**Results**

During the study period, there were 10,672 patients visiting the site with 4265 LTUP (42%) and 497 deaths and 646 transfer to other sites. The median time before LTFU was: seven months (interquartile range: one to ninteen month): of the 4265 (13 %) came once. Majority, 2006 (47%) of the LTFU occur within the first six months with an incidence rate of 356 per 1000‐person years. Factors associated with becoming LTFU included: young age female (20 to 29 years); adjusted hazard ratio (aHR): 2.05, 95% CI: 1.78 to 2.37, *p* = 0.0001; no education (aHR) 1.94, 95% CI: 1.51 to 2.48, *p* = 0.0001; with low income (aHR) 1.27, 95% CI: 1.03 to 1.57; *p* = 0.0001; detectable viral load at last visit (VL) (aHR) 2.51, 95% CI: 2.15 to 2.92, *p* < 0.0001; low BMI (<18.5) (aHR) 3.70, 95% CI: 2.92 to 4.69, *p* < 0.0001. Patients that previously had irregular visit records had a higher risk (aHR): 0.62, 95% CI: 0.26 to 0.40, *p* = 0.0001 of becoming LTFU than those that had not.

**Conclusion**

Despite dramatic reduction in HIV prevalence in Haiti, the incidence rate of LTFU is high; mainly within the first six months. Intensify measures to improve quality and condition of life must be prioritised in patients with limited resources that are at increased risk of becoming lost to follow‐up [1‐4].

**References**

1. Mberi MN, et al. Determinants of loss to follow‐up in patients on antiretroviral treatment, South Africa, 2004 to 2012: a cohort study. BMC Health Serv Res. 2015;15:259.

2. Kaung Nyunt KK, et al. Factors associated with death and loss to follow‐up in children on antiretroviral care in Mingalardon Specialist Hospital, Myanmar, 2006 to 2016. PLoS One. 2018;13(4):e0195435.

3. Kaara F. Factors associated with loss to follow up of adult patients living with HIV in Kiambu County and Referral Hospital, Kiambu County, Kenya. Global J Health Sci. 2017;2(2):1–19.

4. De La Mata NL, et al. Loss to follow‐up trends in HIV‐positive patients receiving antiretroviral treatment in Asia from 2003 to 2013. J Acquir Immune Defic Syndr. 2017;74(5):555–62.

## O214

### Keynote Lecture: The Future of ART and HIV Cure

**D Kuritzkes**

Harvard Medical School and Brigham Women's Hospital, Boston, MA, USA

The advent and global rollout of triple‐drug combination antiretroviral therapy (ART) for the treatment of HIV infection has resulted in a dramatic reduction in morbidity and mortality from this disease worldwide. Despite the availability of once‐daily single‐tablet regimens, the need for daily administration poses a burden for many patients. The development of long‐acting formulations, including the combination of cabotegravir plus rilpivirine; the novel nucleoside reverse transcriptase translocation inhibitor MK8591; the investigational HIV capsid inhibitor GS‐CA2; and broadly neutralising antibodies (bNAbs) permit administration of ART weekly, monthly, quarterly or even less frequently. Such advances may greatly simplify drug administration and increase adherence to ART for many patients. According to press releases, the phase 3 trials of injectable cabotegravir plus rilpivirine have met their non‐inferiority endpoint; results will be presented formally at CROI in early March, 2019. Although long‐acting formulations may improve the efficacy of antiretroviral therapy or pre‐exposure prophylaxis in persons who have difficulty adhering to daily oral regimens, the very long terminal half‐life of these agents carries the risk of drug resistance, particularly in HIV‐infected persons, who discontinue treatment. Research towards an HIV cure or a treatment that could lead to durable drug‐free remission remains a high priority for clinicians, investigators and patients. Current efforts at eradicating HIV infection or inducing long‐term ART‐free remission include activation of HIV transcription in latently infected CD4+ T cells; enhancing HIV‐specific immunity in order to target and destroy cells harbouring latent infectious proviruses; and cell‐based therapies using genetically modified CD4+ T cells or haematopoietic stem cells. Several exploratory human pilot studies are underway with each of these approaches, but none have borne fruit to date. The difficulty of quantifying the HIV reservoir, the uncertain safety of the experimental treatments under study, and the need to balance the risk of these interventions against the generally well‐tolerated and proven efficacy of long‐term ART remain significant challenges.

## O22 – HIV and Ageing

## O221

### HIV and ageing: a role for inflammation?

**P Hunt**

University of California San Francisco, San Francisco, CA, USA

While HIV‐infected individuals with access to modern antiretroviral therapy (ART) have experienced a dramatic improvement in life expectancy, they remain at higher risk than the general population for morbidity and mortality, particularly from non‐AIDS complications typically associated with ageing. While lifestyle factors (e.g. smoking, drug use, obesity, etc.) as well as ART toxicities likely play a role, it is now well recognised that abnormal immune activation and inflammation persist in many ART‐suppressed individuals – independent of lifestyle factors – and that the extent of these immunologic defects strongly predicts morbidity and mortality from non‐AIDS conditions. While the inflammatory state can be attenuated by early ART initiation, it may persist even in individuals who start ART at high CD4+ T‐cell counts, but appears to predict a narrower set of infectious and neoplastic morbidities in this setting. Multiple causes of the persistent inflammatory state in treated HIV infection have been proposed including HIV persistence, microbial translocation, CMV and other prevalent coinfections. While earlier initiation of ART appears to be beneficial in reducing the inflammatory state and in reducing some morbidities, and some commonly used medications with anti‐inflammatory properties (e.g. statins) are being studied in clinical outcomes trials, there is a clear need for effective interventions to reverse persistent immune activation in this setting. These issues will become increasingly important as the HIV epidemic gets older, particularly in resource‐limited settings, where the vast majority of HIV‐infected individuals live.

## O222

### Ageing gracefully with HIV: challenges and goals

**J Falutz**

Montreal General Hospital, Department of Geriatrics, Montreal, Canada

Long‐term survival of treated people living with HIV (PLWH) currently approaches that of the general population, with cofactors modulating individual outcomes. This reality, coupled with an increasing age at seroconversion, has resulted in ageing of the PLWH population. In resource‐rich countries most PLWH are older than 50 and this proportion will continue to increase. Similar trends occur in most other parts of the world. This profound impact of cART is however associated with sobering clinical realities. The profile of ageing PLWH resembles that of the general population about five to ten years older. In addition to the earlier occurrence of common age‐related conditions, multimorbidity has also increased. This is associated with polypharmacy and its complications. Furthermore, other common geriatric syndromes also impact this younger population. These conditions are challenging to evaluate and manage, and include impaired mobility and falls, sensory complaints, age‐related body composition changes, impaired cognition and frailty. Frailty is relevant as it increases other serious health outcomes. Frailty is a state of increased vulnerability to biologic and environmental stressors, with reduced ability to maintain homeostasis. In the general population, a simple and reliable test for frailty in the clinic remains elusive, although several are used in research settings. The investigation of common determinants of frailty in both the geriatric and PLWH populations, including immune‐senescence, chronic inflammation, epigenetics and mitochondriopathy, will improve prevention and management and provide insight into the described discordance between chronologic and biologic age. While research investigates whether PLWH represent a model of accelerated versus accentuated ageing, evaluation of predictors of successful ageing in PLWH is ongoing. Emerging insights into psychological and physical resilience will contribute to both increased lifespan and improved healthspan for PLWH.

## O223

### Fragility fractures and risk assessment in a cohort of adult women living with HIV in Argentina

**M Laurido, I Cassetti and R Mauas**

Infectious Diseases, Helios Salud, Buenos Aires, Argentina

**Background**

A rising number of women living with HIV (WLHIV) are now reaching menopausal age. Low bone mineral density (BMD) is a common finding with elevated fracture risk (FR) in this population. Modified‐FRAX® is widely used for fracture prediction but a few concerns about its accuracy have arisen recently. The aim of this study was to evaluate the prevalence of fragility fractures (FF) and associated factors in a cohort of adult WLHIV receiving care at our institution.

**Material and methods**

A retrospective, observational study including WLHIV over 50 years old attending an HIV reference centre in Buenos Aires, Argentina from 1997 to 2017 was performed. Clinical, laboratory and demographic data were reviewed assessing the prevalence of common comorbidities, including low BMD measured by DXA scan, and FF defined as events evidenced by imaging techniques that occurred spontaneously or by low‐level trauma. FR assessment was performed using modified‐FRAX®. Information was collected in an *ad hoc* database and *t*‐test, chi‐square, Fisher exact or mid‐P tests, and maximum likelihood odds ratio were used as appropriate. Variables with *p* < 0.15 were included in a multiple regression model.

**Results**

Two hundred and fifty patients were included. Demographic and clinic characteristics are shown in Table 1. The prevalence of low BMD was 67% (88/132 women) and FF 6.4% (18 events in 16 patients). Factors associated with FF according to multiple regression model were: increased age (*p* = 0.007), HCV coinfection (*p* = 0.001), increased modified‐FRAX score for hip fracture (*p* = 0.002) and major osteoporotic fracture (*p* < 0.001). Those patients with FF showed a 75th percentile in current hip and major osteoporotic fracture scores of 1.1 and 5.6, respectively; and patients without FF showed a 75th percentile for each scores of 1.2 and 4.7 respectively (Figure 1). Taking into account the latest percentiles as cutoff values, scores for hip fracture ≥1.2 or major osteoporotic fracture ≥4.7 were associated with a significantly higher likelihood of FF with an OR: 6.7 (95% CI: 1.9 to 29.4, *p* < 0.01) and OR: 11.9 (95% CI: 3.1 to 67.2, *p* < 0.001) respectively.

**Conclusions**

Risk factors for FF found in this study were similar to those previously described in the literature, highlighting the impact of HCV coinfection. Despite known modified‐FRAX® limitations, we believe that a new cutoff could be more accurate for risk prediction and deserve further investigation in a prospective study.

**Abstract O223‐Table 1. Demographic and clinical characteristics**

**Table:** 

Variables	Results
Mean age, years (SD)	58.1 (6.1)
Mean time since HIV diagnosis, years (SD)	13.8 (6.9)
Mean BMI, kg/m^2^ (SD	26.9 (5.1)
Habits, n (%)	
Current or former smoker	116 (46.4)
History of alcohol/substance abuse	22 (8.8)
Hepatitis C coinfection, n (%)	21 (8.4)
AIDS at diagnosis, n (%)	116 (46.4)
On cART, n (%)	246 (98.4)
Mean total cART duration, years (SD)	11.7 (5.9)
Plasma HIV‐1 RNA <20 copies/mL, n (%)	217 (88.2)
Mean current CD4 T‐cell count, cells/μL (SD)	673 (312)

Abstract O223‐Figure 1. Modified‐FRAX® for hip fracture (1) and major osteoporotic fracture (2) in patients with (A) and without (B) fragility fractures. Box‐Plot.

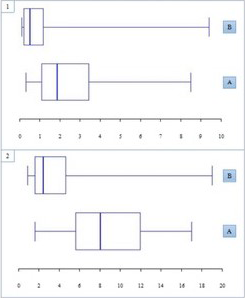

## O23 – Key Topics in Hepatitis

## O231

### Non‐viral liver disease and HIV/NASH

**M Pessôa**

University of São Paulo, São Paulo, Brazil

The investigation of normal liver enzyme in patients infected with HIV can be as challenging as it is for a patient negative for any hepatotropic virus. In the absence of alcohol abuse, we have to keep in mind the possibility of drug‐induced liver injury (DILI), especially in patients with polipharmacy and cART. Non‐alcoholic fatty liver disease (NAFLD) is increasingly recognised as an important aetiologic factor of chronic liver disease in the HIV‐infected population. As NAFLD can coexist with other liver diseases, it can be also responsible for a faster progression of fibrosis towards cirrhosis. The term NAFLD includes a wide spectrum of liver disease, from simple steatosis to steatohepatitis with different grades of fibrosis, including cirrhosis. NAFLD is also recognised as an important risk factor for the development of hepatocellular carcinoma, independent of the presence of cirrhosis. The prevalence of NAFLD is extremely variable depending on the geographical area, probably due to different diet behaviour in the different continents. Overall, the prevalence is estimated to be around 25% in the general population. In the HIV‐infected population, prevalence is described to range from 13% to 73%, with this wide variation probably due to the different methods for fatty liver assessment. Although up to now, only life style modification is effective in the management of this NAFLD, several drugs are under investigation, including antifibrotic drugs, with interesting results in this disease, who is becoming the leading aetiology in the liver transplant list in several countries.

## O232

### Hepatitis C eradication: is it for real in this day and age of economic constriction?

**J Santana**

University of Puerto Rico School of Medicine, San Juan, Puerto Rico

Hepatitis C has become an alarming public health threat, becoming the number one cause of end‐stage liver disease and of liver transplant in many countries. New short duration and simple all oral treatment options have demonstrated efficacy close to a 100% of cure in treated patients. Theoretically, both scientifically and mathematically, eradication of the disease is feasible but will require great efforts to diagnose patients who are unaware of their infection as well as real commitment from the sociopolitical and cultural arena to achieve this goal allocating the required resources. We are nevertheless in a historic junction in modern medicine where the feasibility of eradicating a global disease is being contemplated and might be possible with simple tools of prevention, linkage to care and curative oral treatment. Unfortunately, hepatitis C is still not considered in many countries a public health priority despite great efforts by many scientific and health regulatory agencies including the World Health Organization. In addition, there has been no iconic figure identified who can raise the emotional passion of collective political and community awareness as we have seen with other chronic and terminal illnesses such as HIV and cancer. Individuals as well as the civil society need to thrive with determination, partnerships, collaboration and advocacy to build and sustain support for these efforts. Upfront fair and just negotiations with government‐pharmaceutical leverage needs to be played in order to lower the price even further of these highly effective medications. We can and we must as individuals within the civil society stimulate the collective awareness in order to screen, identify and diagnose at risk individuals so we can sensitise politicians, key stakeholders and opinion leaders to the reality of response that a secure initial investment will help save many lives and will bear long‐term benefits to the society as a whole. Strong and sustained political, community advocacy in conjunction with people living with the disease is needed in order to build and support effective measures. If this is to happen in our lifetimes, the time for action needs to wait no longer.

## O233

### The impact of HEV infection on the disease severity of patients with chronic HCV infection

**P Yoshimura Zitelli^1^, M Gouvea^1^, J da Motta Singer^2^, A Ferreira^1^, J Pinho^1^, V Avancini Ferreira Alves^3^, R Tanigawa^3^, F Carrilho^1^ and M Pessôa^1^**

^1^Department of Gastroenterology and Hepatology, Hospital of Clinic of Faculty of Medicine of University of São Paulo, São Paulo, Brazil. ^2^Department of Statistics, University of São Paulo, São Paulo, Brazil. ^3^Department of Pathology, Hospital of Clinic of Faculty of Medicine of University of São Paulo, São Paulo, Brazil

**Background**

In Brazil, hepatitis E (HEV) is rare, with a prevalence ranging from 1% to 12.9% in healthy subjects. It is known that in patients with chronic hepatitis C (HCV), hepatic lesions may be exacerbated by coinfection with hepatitis A virus (HAV) or hepatitis B virus (HBV) [1]. However, few studies have evaluated HCV coinfection with HEV. Therefore, we evaluated the impact of HEV infection on the disease severity of chronic HCV infection. The aims were to determine the prevalence of HEV infection among patients with chronic HCV, assessing the impact of coinfection on liver disease severity and to evaluate histological characteristics to HEV infection in HCV‐HEV coinfected patients.

**Materials and methods**

A observational and cross‐sectional study was performed. Samples were collected from 2013 to 2016. Inclusion criteria were consecutive chronic HCV adults; naïve for HCV treatment. An exclusion criteria was HBV or HIV infection. HEV serology was performed with IgG and IgM antibody assays (ELISA, Mikrogen, Germany) and, HEV RNA by QIAamp® MinElute® Virus Spin (Qiagen, United States of America). Liver histology was analysed with a 1:2 pairing of monoinfected and coinfected patients.

**Results**

Anti‐HEV IgG was positive in 22/181 (12.0%) patients, anti‐HEV IgM was positive in 3/181 (1.6%) patients and real‐time HEV RNA was positive in 9/181 (4.9%) patients. When comparing patients with and without HEV coinfection, we observed a significant difference in the following laboratory parameters: APRI score (*p* = 0.013), albumin (*p* = 0.01), total bilirubin (*p* = 0.007), platelets (*p* < 0.001) and INR (*p* = 0.032). In addition, HEV‐positive cases also had a higher severity of liver disease in the following clinical parameters: fibrosis ≥3 vs. ≤2 (*p* = 0.001), oesophageal varices (*p* = 0.007), ascites (*p* = 0.010) and CHILD‐PUGH score (*p* = 0.036). Of the 96 patients who underwent liver biopsy, 11 had a positive serological marker for HEV (Table 1). We then performed a 1:2 match for age, gender, HCV genotype and grade of liver fibrosis. No particular histological abnormality could be attributed in HEV coinfection when compared to HCV monoinfected patients.

**Conclusions**

The prevalence of anti‐HEV IgG (12.0%) in chronic HCV infection was not higher than in the general population. HEV infection had an important impact on severity of liver disease in our chronic HCV patients. When paring liver biopsies with the same amount of fibrosis, we could not observe any particular characteristic of HEV coinfection.

**Absract O233‐Table 1. Compared of HEV positive with HEV negative relatively with category variable**

**Table:** 

	Hepatitis E						IC95%		*p* value
	NO	%	YES	%	Total	OR	Inf.	Sup	
Fibrosis									
Grade ≤2	95	90.5%	10	9.5%	105	1
Grade ≥3	54	71.1%	22	28.9%	76	3.87	1.7	8.7	0.001
Total	149	82.3%	32	17.7%	181	‐	‐	‐	‐
Oesophageal varices									
No	112	86.8%	17	13.2%	129	1
Yes	31	68.9%	14	31.1%	45	2.97	1.32	6.69	0.007
Total	143	82.2%	31	17.8%	174	‐	‐	‐	‐
Encephalopathy									
No	138	83.1%	28	16.9%	166	1
Yes	3	50.0%	3	50.0%	6	4.92	0.94	25.6	0.073
Total	141	82.0%	31	18.0%	172	‐	‐	‐	‐
Ascite									
No	134	84.8%	24	15.2%	158	1
Yes	9	56.3%	7	43.8%	16	4.34	1.47	12.7	0.010
Total	143	82.2%	31	17.8%	174	‐	‐	‐	‐
CHILD‐PUGH score									
A	36	80.0%	9	20.0%	45	1
B	10	52.6%	9	47.4%	19	3.6	1.12	11.4	0.036
Total	46	71.9%	18	28.1%	64	‐	‐	‐	‐

**Reference**

1. Sagnelli E, Sagnelli C, Pisaturo M, Coppola N. Hepatic flares in chronic hepatitis C: spontaneous exacerbation vs hepatotropic viruses superinfection. World J Gastroenterol. 2014;20(22):6707–15.

## O31 – Public Health HIV Programmes of Latin America and the Caribbean Moving Beyond NNRTI‐Based First‐Line Regimens

## O311

### Landscape of current HIV care and treatment policies and practice

**G Ravasi**

Pan American Health Organization, Washington, DC, USA

In 2014, Latin America and the Caribbean (LAC) countries endorsed the “90‐90‐90” targets by 2020 as a milestone towards ending AIDS. By December 2017, 77% of people with HIV in Latin America (LA) knew their status, 61% were receiving antiretroviral therapy (ART) and 52% were virally suppressed, respectively, 73%, 57% and 40% in the Caribbean (CAR). Approximately 1.3 million were receiving ART in LAC. Significant gaps persist and countries will require efforts and investments to expand and innovate services, improve their effectiveness, efficiency and sustainability. Data on policy uptake and practices are derived from Global Aids Monitoring reports and PAHO desk review.

In 2018, the regional uptake of the WHO‐recommended “treat all” policy was 82% (27/33 countries); six countries are updating their guidelines in 2019. Only eight countries exceeded 60% ART coverage (Argentina, Brazil, Cuba, Guyana, Haiti, Mexico, Peru and Trinidad and Tobago). HIVDR to NNRTI is increasing in LAC, and 61% countries (20/33) already introduced DTG in their guidelines (nine are planning). Only six countries initiated the transition to preferred DTG‐based first‐line regimens (in remaining countries DTG is for alternative regimens (nine countries) or one of multiple first‐line options (four countries). Between 2010 and 2014, the number of ART sites increased in 62% of countries (16/26; 46% CAR, 77% LA). In 2017, 45% countries (14/31) already established ART services in the first level of care by primary care providers (8 CAR; 6 LA). ART integration into general care is still scarce: 41% (12/29) countries reported ART delivered separately from general care in all facilities; 41% (12/29) integrated in some; fully integrated only in five countries. Challenges in integrated service for HIV‐TB coinfected persons: 23% of countries (7/31), ART is available in TB clinics by TB providers (40% CAR; 6% LA); in 55% (17/31) TB treatment is available in ART settings by ART providers (40% CAR; 63% LA); in 23% (7/30) ART and TB treatment are delivered separately. Same challenges apply to other comorbidities such as viral hepatitis and HPV. Service delivery for HIV care should be decentralised, integrated based on a person‐ and community centred approach with greater involvement of the first level of care.

## O312

### The emerging threat of HIV drug resistance: findings to accelerate transition

**S Ávila‐ Rios**

Infectious Diseases Research Centre, National Institute of Respiratory Diseases, Mexico City, Mexico

The World Health Organization (WHO) Global HIV Drug Resistance (HIVDR) Report shows pretreatment drug resistance (PDR) levels to non‐nucleoside reverse transcriptase inhibitors (NNRTI) approaching 10% in several low/middle‐income countries (LMICs) that have performed nationally‐representative surveys. HIVDR can potentially result in increasing mortality, HIV incidence and treatment costs, threatening UNAIDS goals to control the HIV pandemic by 2030. The WHO Global Action Plan on HIVDR recommends an urgent public health response to high PDR levels, moving away from NNRTI in settings where PDR to this drug class is higher than 10%. Some LMICs, including Botswana, Kenya and Brazil, have already initiated transition to first‐line regimens containing low‐cost generic dolutegravir. During this talk, we will review results on nationally representative PDR surveys performed by Latin American countries. We will also discuss progress made in the public health response to high NNRTI PDR levels in the region, as prevalence of efavirenz/nevirapine resistance in persons starting or restarting first‐line ART at the national level is reaching worrisome levels in several countries: Argentina 10.9% (95% CI: 8.2 to 14.3), Mexico 10.1% (8.9 to 11.5), Guatemala 13.2% (8.8 to 19.4), Nicaragua 19.3% (12.2 to 29.1), and Honduras 27.3% (21.1 to 34.5). This is particularly important in the Latin American context, where a large proportion of countries maintain efavirenz/nevirapine‐based regimens as preferred first‐line options in their guidelines. To achieve UNAIDS global targets, enhanced strategies to curb HIVDR are warranted, including rollout of low‐cost generic drug combinations with better efficacy and high genetic barrier to resistance, improved access to viral load monitoring, availability of HIVDR testing and reliable drug supply chains.

## O313

### Experience with dolutegravir in first‐line treatment in Brazil: the first 100,000 patients

**A Pati Pascom**

Department of STI, HIV/AIDS and Viral Hepatitis, Ministry of Health, Brasilia, Brazil

Brazil offers universal and free HIV care for all people living with HIV. Since January 2017, the Ministry of Health of Brazil (MoH‐B) has included 3TC+TDF+DTG as the preferred first‐line regimen and recommended the switch from RAL‐based regimens. Furthermore, in 2018, Brazilian guidelines included the switch for those who were virologically suppressed. By December 2018, around 200,000 PLHIV were using DTG‐containing regimens, among those 57% were treatment naïve and 16% switched from EFZ‐containing regimens. Studies conducted by the MoH‐B showed the superiority of DTG‐containing regimens when compared to other first‐line regimens. Odds ratio of failing to achieve viral load suppression, with 3TC+TDF+DTG as the reference and controlling for possible confounders, vary from 1.42 for 3TC+TDF+EFZ to 2.62 for 3TC+TDF+LPV/r. A recent study revealed that suppression is faster among those with DTG‐containing regimen compared to EFZ‐containing; for example, 81% of individuals that started with DTG‐containing regimens presented viral load below 50 copies/mL after three months of treatment and only 61% among those with EFZ‐containing. In another implementation study, it was observed that 3TC+TDF+DTG led to a higher CD4 cell recovery after the first year of treatment than did 3TC+TDF+EFZ, in all strata of CD4 analysed, and after controlling for possible confounders. MoH‐B adopted two strategies to offer DTG without major changes in its annual budget: price negotiation and reorganisation of the drug portfolio, including withdraw of obsolete drugs and major switch to the new regimens. Thus, MoH‐B offers the best HIV therapy available nowadays, maintaining the sustainability of universal access to ARV.

## O314

### ART and sexual and reproductive health of women living with HIV: challenges and opportunities

**R Zash**

Reproductive Health, Harvard University Center for AIDS Research, Cambridge, MA, USA

Paediatric HIV infections have been dramatically reduced because of the scale‐up of antiretroviral treatment (ART) among all adults, including pregnant women. Now, the magnitude of ART exposures in pregnancy in countries with high HIV prevalence outweighs that seen for any other drug in history. An increasing proportion of women start ART prior to conception, leading to more ART exposures in the first trimester when the foetus is most vulnerable to teratogens. Despite the magnitude of ART exposures, data on the safety of ART in pregnancy has lagged behind. Recent data from Botswana suggests a potential increased risk of neural tube defects with dolutegravir (DTG) exposure from conception. This signal has put country programmes, healthcare providers and women living with HIV in a difficult position with regard to HIV treatment decisions. This presentation will review recent data on ART safety in pregnancy, highlight knowledge gaps and discuss how to compare pregnancy safety data from varied settings. Despite the challenges presented by the Botswana findings, there is also now an opportunity to address reproductive health of HIV‐infected women more broadly. This includes improving access to contraception and family planning, creating models for individualised HIV treatment decisions in lower resource settings and expanding surveillance to more efficiently gather pregnancy safety data on new antiretroviral medications.

## O315

### The role of civil society in the evolving HIV treatment programmes: lessons learned and recommendations

**J Hourcade Bellocq**

Buenos Aires, Argentina

During the first darker decade of the epidemic, civil society (CS) organised a movement to fight stigma and discrimination, putting pressure on governments and scientist to find a cure or a treatment, including proper resource allocations. With highly effective treatment, the CS moved into treatment access advocacy and literacy, supporting adherence. One of the key stakeholders in the creation of the Global Fund (GF) and its architecture were the CS including people living with HIV (PLWH) movement and this resulted in a scale‐up to 17 million people on antiretroviral therapies (ARVs). Today, CS and PLWH are critical in replenishing the GF and in discussing a proper allocation. Under the new global funding for health trends, the investment of Official Development Assistance is moving away from middle‐income countries where most of the people live, jeopardising some of the gains of the grant investments. The introduction of ARV‐based prevention and the evidence Undetectable=Untransmittable (U=U) changes the landscape of the response. The role that CS and PLWH could play campaigning on U=U will significantly decrease stigma, discrimination and criminalisation of HIV transmission, that is a massive incentive for more people to come forward for HIV testing. PrEP requires accessing a diverse and heterogeneous population that is out of the reach of CS. The primary challenge is how to build demand on potential users and pressure on decision‐makers for funding PrEP access. CS needs to rethink itself and to create a new narrative around the current response that we need. There are lessons to be learned, but it will not be enough.

## O32 – Barriers and Facilitators to Early Treatment and PrEP Implementation in the Region

## O321

### PrEP 2019: where are we now?

**K Mayer**

Harvard Medical School and Beth Israel Deaconess Hospital, Boston, MA, USA

Over the past decade, multiple randomised controlled trials and demonstration projects have shown that tenofovir disoproxil fumarate/emtricitabine (TDF/FTC) taken daily as antiretroviral pre‐exposure prophylaxis (PrEP) is well‐tolerated, safe, and effective in preventing HIV transmission in at risk individuals. Since then more than 350,000 individuals have had PrEP prescribed, with the majority being from the United States, but active programmes have been established in several countries in Europe, Latin America, Africa, Asia as well as Australia and Canada. However, the majority of individuals who might benefit from PrEP have not yet accessed it, often due to residual structural barriers (e.g. costs, unsupportive healthcare professionals, medical mistrust). In addition, rates of persistence vary greatly, with the majority of PrEP initiators not continuing for more than a year in the most series. Innovative approaches designed to increase PrEP uptake, adherence and persistence have included training peer health system navigators, engaging other health system professionals (e.g. pharmacists), the use of academic detailing bringing PrEP education to busy clinical practices, the development of clinical support tools (e.g. electronic health record prompts) and the use of social media (i.e. websites and apps) to increase PrEP awareness and to support PrEP engagement. Daily oral TDF/FTC is the first PrEP approach, but on‐demand, pericoital TDF/FTC (two pills within 24 hours of sex and a pill a day for the two subsequent days) has been shown to be effective in a study of French and Canadian MSM. TAF/FTC is being evaluated for PrEP, with efficacy trial data expected to be presented soon. A dapivirine‐containing intravaginal ring inserted monthly has been shown to decrease HIV incidence in African women, and is being evaluated for licensing. Clinical trials of injectable cabotegravir, an integrase strand transfer inhibitor, and monoclonal antibodies (i.e. immunoprophylaxis) are also underway. Other approaches, ranging from rectal douches to implantable antiretroviral‐containing rods, are in earlier stages of clinical development. Having multiple choices may assist in scaling up PrEP to the extent that it can have wide population‐level impact in decreasing HIV incidence, as part of a combined public health programme, along with treatment as prevention.

## O322

### The Brazilian implementation plan

**A Schwartz Benzaken**

São Paulo, Brazil

Since 2013, the Ministry of Health (MoH) of Brazil has co‐financed PrEP implementation studies. In 2017, the MoH approved PrEP as the national policy and launched the PrEP national guideline. The first purchase of emtricitabina+tenofovir disoproxil fumarate was performed after registration by the national regulatory agency. The presentation aims to describe the implementation, the scaling up and the monitoring of PrEP offer in the free‐of‐charge Brazilian Health System (SUS). In one year of PrEP, the country has reached a total of 7418 PrEP users in 82 services through the country. In spite of this progress, there is still a need to accelerate the availability of PrEP to the vulnerable population nationwide to really impact the HIV epidemic.

## O323

### The case of Peru

**C Cáceres**

AIDS and Society, Cayetano Heredia University, Center for Interdisciplinary Research on Sexuality, Lima, Peru

**Background**

Ongoing demonstration projects are helping document and resolve difficulties that emerge in PrEP rollout. Among them, ImPrEP, conducted in Brazil, Mexico and Peru, is enrolling eligible MSM and transgender women (TW) aged 18 or older. Here, we present an interim analysis of the ImPrEP implementation process in nine public study sites in Peru.

**Methods**

We conducted eight semi‐structured interviews with health providers in three cities: Lima/Callao, Pisco and Pucallpa, to identify key issues in programme implementation. We also analysed monitoring data of the PrEP implementation process in the nine public sites, to identify: basic existing infrastructure and gaps; time of implementation; and emerging issues affecting enrolment and quality of care.

**Results**

The Ministry of Health co‐sponsored the study, although facilities were free to get involved. The programme generated interest both among providers and potential users. Providers expressed concerns about work overload, post‐study PrEP availability and drug resistance. For implementation, basic infrastructure gaps in each site were addressed (e.g. laboratory equipment, furniture, space renovation) in variable periods (from three to nine months) according to varying fluidity of internal procedures across sites. Once enrolment started, new bottlenecks became visible: effective limited time for blood sample collection (most sites); limited/unpredictable availability of physicians (five sites), long waiting times (most sites). Morning working times were too inconvenient for transwomen.

**Conclusions**

Providers and users of public facilities are interested in PrEP. However, well‐planned and sustainable PrEP rollout in these public facilities implies some kind of upgrade in the general conditions of HIV prevention services, hence offering the opportunity to invest in strengthening and revamping the overall programmatic response to HIV in Peru and similar countries.

## O324

### Sexual health clinics and PrEP implementation in Buenos Aires

**M Losso**

HIV Unit, Hospital Ramos Mejia, Buenos Aires, Argentina

The advancements in prevention of HIV infection and the needs of new strategies to achieve a better diagnosis and treatment of sexually transmitted infections (STIs), requires innovative ideas in order to attain its effective implementation in different settings. Previous experience with the sexual health clinics network in New York, USA and the Dean Street Clinic in London, UK provides evidence of new avenues to explore. The experience of ClinSex in Buenos Aires, a creative alternative for the provision of proven prevention and treatment strategies of HIV infection and STIs is the main objective of this presentation. ClinSex arise to address the unserved sexual health of several populations, regardless of gender, sexual preferences or lifestyles. A multidisciplinary team including community managers, counsellors, nurses, psychologists, pharmacists and physicians was established. ClinSex (Clinsex.org) is a sexual health clinic that provides friendly, easy and free access to prevention and treatment of STIs and HIV infection. The Clinic is located in the Hospital Ramos Mejia in the densely populated neighbourhood of Balvanera in Buenos Aires. Services provided includes diagnosis of HIV and syphilis through rapid tests, diagnosis of viral hepatitis and STIs, counselling, harmonisation and specialist consultation, access to clinical research trials, same day appointment with an HIV specialist and early access to ARV. The achievement of and effective channel of communication with the people at risk and the establishment of innovative ways of reaching these populations is one of the main assets of the strategy that reached 3000 consultations in the first year.

## O325

### PrEP implementation in transgender women

**F Badial Hernandez and E Roman**

Clínica Especializada Condesa Iztapalapa, Mexico City, Mexico

Transgender women (TGW) represent 3% of all the new HIV infections in Latin America. A study done in TGW in meeting places in Mexico City reported 19.8% prevalence. Only 26% of those with a positive result in this study were aware of their serostatus. A hormone substitution therapy clinic for both HIV‐positive and HIV‐negative TGW and men started in 2009 in the Mexico City's HIV Programme “Condesa Clinics” with a total registry of 2229 persons by 2018, 77% of which are TGW (24.5% HIV positive). A PrEP demonstration project for TGW and men who have sex with men (MSM) was initiated in July 2018 in the Condesa Clinics and in local non‐governmental organisations (NGOs). Since then 724 people have sought PrEP, of which only 11 (1.52%) are TGW. In contrast, 313 TGW asked for an HIV test in the Clinics’ voluntary counselling and testing (VCT) service in 2018; 251 (80.2%) were HIV negative. Even if TGW represent only 1.05% of the people asking for an HIV test in the VCT service, the number of TGW asking for PrEP remains low. Fear of adverse events and of drug–drug interactions with hormone therapy as well as low PrEP awareness have been reported. TGW in our cohort have also expressed fear of stigmatisation for taking HIV drugs. In addition, in previous PrEP studies TGW with high‐risk behaviours have shown lower adherence to PrEP than MSM with similar behaviours. Evidence‐based communication and linkage‐to‐care interventions specific for TGW are needed to increase the number of TGW asking for and adhering to PrEP.

## Poster Abstracts

## Co‐Morbidities and Complications of Disease, Non‐AIDS Morbidity and Cancers

## P001

### Significant decrease of HIV/AIDS mortality in Mexico during the last decade

**E Bravo‐García^1^ and H Ortiz‐Pérez^2^**

^1^Censida, Mexico City, Mexico. ^2^Departamento de Atención a la Salud, UAM Xochimilco, Mexico City, Mexico

**Background**

Free and universal access to HAART in Mexico since 2003, involved the construction of new specialised medical units (CAPASITS) and the adaptation of services within hospitals (SAIH); the updating of national guidelines for the management of HAART and training of treating physicians. In the first years of its application (2003 to 2007), the network of CAPASITS and SAIH gradually spread throughout the country, which allowed an increase in the number of people receiving HAART. Starting in 2008, the mortality for HIV finally began to decrease significantly.

**Materials and methods**

Using official information on deaths (INEGI) and population estimates (CONAPO), crude and standardised rates were calculated, according to sex, age groups, place of residence and access to social security health services.

**Results**

From 1990 to 2017, 119429 HIV deaths occurred in Mexico, representing about 1% of the total. Deaths grew continuously from 1990 to 2008, reaching 5183 in 2008, the highest figure in history. With the consolidation of access to HAART, between 2008 and 2017 mortality decreased 17% in both sexes; 18% in men in the same period; and 23% in women, as of 2009. Despite this reduction, 8/32 entidades federativas increased their rate from 2013 to 2017. Tabasco (9.7 per 100 thousand inhabitants), Quintana Roo (9.5) and Campeche (8.5) had the highest rates; while Zacatecas (1.2), Guanajuato (1.6) and Hidalgo (1.7), the lowest. It is unacceptable that in Tabasco, HIV mortality was eightfold than Zacatecas (Figure 1).

**Conclusions**

A decrease of HIV/AIDS mortality in Mexico shows the impact of the policy of free and universal access to HAART, driven by Censida since 2003. However, for this trend to continue, it is necessary to significantly increase the early diagnosis of people with HIV, and its link to HAART, especially in the entidades federativas with the highest mortality rates.

Abstract P001‐Figure 1. HIV mortality in México from 1990 to 2017 by sex.

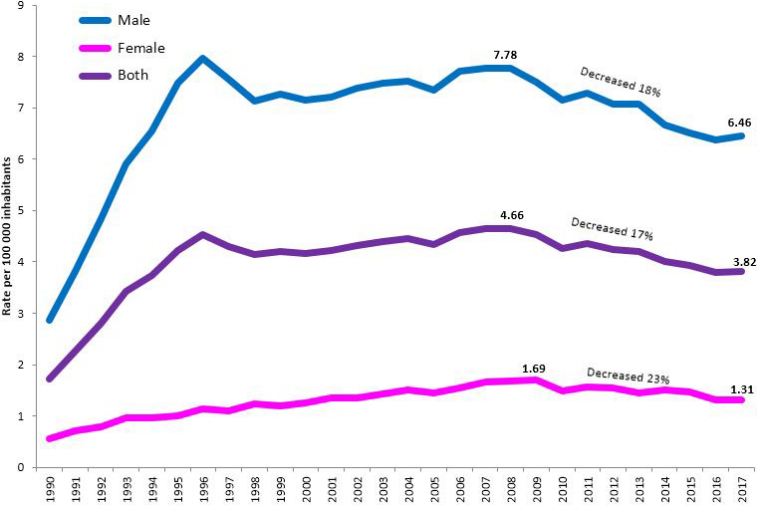

## P002

### Clinical characteristics of HIV‐positive patients with lymphoma in a third‐level hospital in Mexico City

**A Cárdenas‐Ortega^1^, F Rivera‐Buendía^1^, A Ramirez‐Ibrarguen^2^ and A Martin‐Onraët^1^**

^1^Department of Infectious Diseases, Instituto Nacional de Cancerología, Mexico City, Mexico. ^2^Department of Haematology, Instituto Nacional de Cancerología, Mexico City, Mexico

**Background**

HIV infection is a predisposing factor for lymphomas (AIDS and non‐AIDS associated). After the advent of antiretroviral therapy, the epidemiology of lymphomas has changed in international cohorts. So far, the literature including data from Mexico is scarce.

**Materials and methods**

This retrospective study included all adult patients with HIV and lymphoma diagnosis from January 2010 to December 2017 at the Instituto Nacional de Cancerología, Mexico City. Data obtained from the electronic files are described as frequencies and measures of central tendency. We used T test or Mann–Whitney tests for quantitative variables, and chi‐squared analysis for qualitative variables. We evaluated variables associated with death by logistic regression.

**Results**

One hundred and fifty‐five patients were included; 140 (90%) were men and the median age was 40 years (31 to 48). Ninety per cent were previously healthy, 52% smoked, 29% reported alcohol abuse and 19% reported other substance abuse. Chronic hepatitis B was found in 14 (9%) patients, hepatitis C in 5 (3%) and syphilis in 24 (15%). Non‐Hodgkin lymphoma (NHL) was diagnosed in 132 (85%) patients and Hodgkin's lymphoma (HL) in 23 (15%). Among NHL, 62 (47%) were diffuse B‐cell lymphoma, 34 (26%) plasmablastic, 19 (14%) Burkitt, 10 (8%) primary central nervous system and 7 (5%) others. Of the NHL, 107 (81%) presented with advanced stages (Ann Arbor IV); 57% had a high‐risk international prognostic index (IPI). The median CD4 count at lymphoma diagnosis was 100 (35 to 234) cel/mm^3^for NHL and 167 (106 to 331) cel/mm^3^ for HL (*p* = 0.007). Forty‐five per cent of NHL patients were on ART before lymphoma diagnosis vs. 65% of HL (*p* = 0.069). Of all lymphomas, 37 (24%) patients had an opportunistic infection at lymphoma diagnosis: oesophageal candidiasis (35%), *Pneumocystis jirovecci* pneumonia (24%), CMV (13%), histoplasmosis (13%) and tuberculosis (8%). Fifty‐one opportunistic and 231 non‐opportunistic infections were diagnosed during the first year after chemotherapy (Table 1).

**Abstract P002‐Table 1. Baseline characteristics**

**Table:** 

Baseline characteristics	Total population n (%)	NHL n (%) n = 132	HL n (%) n = 23
Gender			
Men	140 (9.3)	119 (90.2)	21 (91.3)
Women	15 (9.7)	13 (9.8)	2 (2.87)
Age, years			
Median, (IQR)	40 (31 to 48)	40 (30 to 45)	36 (31 to 49)
Co‐morbidities			
None	140 (90.3)	119 (90.1)	21 (91.3)
High blood pressure	3 (1.9)	3 (2.3)	‐
Metabolic syndrome	3 (1.9)	3 (2.3)	‐
Others	9 (5.8)	7 (5.3)	2 (8.6)
Substance abuse			
Smoking	80 (51.6)	66 (50)	14 (60.8)
Alcohol	45 (29.0)	38 (28.8)	7 (30.4)
Drug abuse	29 (18.7)	26 (19.7)	3 (13.0)
Other infections			
Kaposi's sarcoma	13 (8.4)	11 (8.3)	2 (8.7)
Skin and mucous	7 (53.8)	7 (63.6)	‐
Disseminated	6 (46.2)	4 (36.4)	2 (100)
Hepatitis B infection			
AgS (+)	14 (9.0)	14 (10.6)	‐
Anti‐core (+)	33 (21.3)	27 (25)	6 (26.1)
Hepatitis C	5 (3.2)	3 (2.3)	2 (8.7)
Syphilis (positive VDRL)	24 (15.5)	24 (18.2)	‐
Lymphoma histopathology			
Diffuse large B‐cell lymphoma	62 (40)	62 (47)	‐
Plasmablastic lymphoma	34 (22)	34 (25.8)	‐
Burkitt's lymphoma	19 (12)	19 (14.4)	‐
Primary central nervous system lymphoma	10 (6)	10 (7.6)	‐
Other NHL	7 (4)	7 (5.2)	‐
International Prognostic Index Risk			
Low	34 (22)	28 (21.2)	6 (26.1)
Medium	34 (22)	29 (22.0)	5 (21.7)
High	86 (49)	74 (56.1)	12 (52.2)
Ann Arbor stage			
I to III	33 (21)	22 (16.7)	11 (47.8)
IV	119 (77)	107 (81.0)	12 (52.2)

**Conclusions**

Overall, 34% of patients died, 41% are currently in remission, 86% had an undetectable HIV viral load at the end of treatment and 14% were lost to follow‐up. The cause of death was attributed to lymphomas in 53%, and infections in 47% (12% due to opportunistic infections, 35% to nosocomial infections). After adjusting for confounders, a high‐risk IPI at diagnosis was associated with death (aOR 6.2, 95% CI: 1.48 to 25.95, *p* = 0.012). In our institution, NHL remains the most frequent lymphoma, mostly due to late HIV diagnosis. The prevalence of opportunistic infections at baseline was high (25%), which underscores the importance of exhaustive workup. As previously reported, a high‐risk IPI had an impact on mortality. More efforts are needed to improve early diagnosis in HIV and lymphoma.

## P003

### Persistent low level plasma HIV RNA in HIV‐infected patients previously virologically suppressed on antiretroviral therapy in a Colombian cohort: a retrospective observational study

**C Herrera‐Díaz, E Maldonado‐Lara, A Sánchez, E Ruíz, D Díaz‐Caicedo, C Angelica, D Hernández, A Jimenez, F Carranza and Y Chaparro**

HIV Infectious Diseases, Mederi‐Universidad del Rosario, Bogotá, Colombia

**Background**

Persistent low‐level viraemia (PLLV) of HIV RNA can be found in previously virologically suppressed HIV patients. When persistent (more than 2 LLVs detected), it becomes a challenge for the clinician. The aim of this study was to characterise patients with PLLV and determine factors associated with virological failure (VF) viral load >200 copies/mL following the detection of PLLV.

**Materials and methods**

A retrospective study was carried out using the database of a third‐level hospital (Barrios Unidos ‐Mederi) in Bogotá, Colombia. It included all the patients on ART with HIV RNA between 50 and 200 copies/mL more than once between 2013 and 2018. Univariate and bivariate analyses were also done.

**Results**

Of the 1238 patients being currently treated in the service, 74 had PLLV (5.97%). The mean age was 49.6 years, 97.2% were men. The mean time of HIV diagnosis was 13.4 years. At the time of diagnosis, most of the patients had less than 200 T CD4 lymphocytes 55% (41/74). ART backbone mostly included tenofovir DF/emtricitabine 52% (39/74). Other agents mostly included: protease inhibitors (PI) 41%, non‐nucleoside reverse transcriptase inhibitors drugs 28%, integrase inhibitors 12% and 17% were with a salvage therapy. 39% (29/74) had syphilis at the time of detection of LLV. In terms of comorbidities, 12% had diabetes, 13% chronic kidney disease, 16% cardiovascular disease, 63% dyslipidaemia, and 18% hypothyroidism. 35% had more than seven other drugs for their comorbidities (polypharmacy). During follow‐up done until 2018, 15 patients (20%) still have detectable viraemia. 12% (9/74) patients progressed to VF. The average time from detection of low viraemia to undetectability was 10 ± 5 months. Of the factors evaluated, polypharmacy was associated with VF (OR 4.24 9 IC 95% 0.059 to 0.043‚ *p* = 0.034) and chronic kidney disease (CKD) with patients who still have detectable viraemia (OR 5.2 9 IC 95% 0.04 to 0.766‚ *p* = 0.012).

**Conclusions**

PLLV following virological suppression is common. In our cohort, most of them are with protease inhibitors. It is well known that PLLV can be due to problems in adherence. It is common in our centre to work in adherence with these patients. 12% progressed to VF and the only factor associated was polypharmacy. On the other hand, CKD is associated with still having detectable viraemia. It is important to evaluate in the future the influence of adherence improvement, reduction of polypharmacy as well as the impact of PI in these patients.

## P004

### AIDS‐related death in Uruguay: high proportion in patients not retained in HIV care

**S Cabrera^1^, D Pérez^1^, J Meré^2^, V Frantchez^3^, C Iglesias^3^ and E Cabeza^3^**

^1^Programa ITS‐VIH/Sida, Ministerio de Salud, Montevideo, Uruguay. ^2^Fondo de Poblaciones, Naciones Unidas, Montevideo, Uruguay. ^3^Enfermedades Infecciosas, Facultad de Medicina, Montevideo, Uruguay

**Background**

AIDS mortality rate has stabilised since 2005 in Uruguay – 5/100.000 inhabitants. But, it has not decreased as expected despite the universal access to care and ART therapy since mid‐1990s. Reducing AIDS mortality rate by 2020 is a priority goal for the Ministry of Health of Uruguay. The aim of this study was to evaluate the distribution of deaths due to AIDS in the HIV cascade of continuum of care.

**Materials and methods**

We conducted a cross‐sectional study through the review of deaths coded as “AIDS” in 18 years old or more in the National Mortality Registry in 2014.

**Results**

We found 175 deaths coded as AIDS, accessing to 124 records, of which only 105 fulfilled the CDC definition of AIDS‐related death. The remaining 19 were deaths due to conditions non‐AIDS related (coding error). The mean age was 43.7 ± 11.6 years, 68% were men, 72% low educational level (nine years), 47.1% drug users, 15.4% were homeless, 15.4% prisoners and 37.5% were included in social assistance plan. The public health system provided care to 77% study sample. Sexual transmission explained 98% of cases, the mean of age between HIV diagnosis and death was 6.2 ± 6.2 years; 72% had CD4 count <200/mL at diagnosis; 43.3% had opportunistic infections (OI) at diagnosis; 64% receipt HAART at any time; 19.4% reached viral load suppressed at any time. HIV late diagnosis was seen in 71.4% and 37.1% of the death occurred in the first year of the diagnosis. Distribution of death in cascade of continuum of HIV care, to AIDS‐related versus those who died due to events unrelated to HIV/AIDS, is shown in Table 1. Mortality AIDS‐related was independently associated with no retention in care (OR 14.09, 95% CI: 1.946 to 700.42); OI at diagnosis (OR 11.66, 95% CI: 1.35 to 514.25); CD4 count nadir for each unit of increase (OR 0.989, 95% CI: 0.984 to 0.999); viral load suppressed before death (OR 0.034, 95% CI: 0.002 to 0.354).

**Conclusions**

High proportion of patients who died due to AIDS before the linkage and follow‐up in the health system demonstrates the need to adopt proven strategies to improve access and continuity of care. In addition, the strategies must be adequate for the conditions of social vulnerability of this population.

**Abstract P004‐Table 1. Distribution of deaths in “cascade of continuum of HIV care,” AIDS‐related deaths versus non‐AIDS–related deaths**

**Table:** 

Step at death	Death with AIDS conditions (n: 105) % (n)	Death with non‐AIDS conditions (n: 19). % (n)	*p* value
At diagnosis	28.6 (30)	0.2 (1)	**0.04**
Before – linkage	11.4 (12)	15.8 (3)	0.7
No retention in care	48.6 (51)	15.8 (3)	**0.011**
Retention in care	8.6 (9)	21.0 (4)	0.11
Retention in care on HAART and VL suppressed 2.9 (3) 42.1 (8) 0.000	2.9 (3)	42.1 (8)	**0.000**

## P005

Abstract withdrawn

## P006

## Stable housing is critical to linkage of newly HIV diagnosed patients to medical care

**D Murphy^1^, E Aguilera^1^, G Del Bianco^1^, G Rodriguez^1^, J Murphy^1^, G Heresi^1^ and S Prater^2^**

^1^University of Texas, McGovern Medical School, Pediatrics, Infectious Diseases, Houston, TX, USA. ^2^University of Texas, McGovern Medical School, Emergency Medicine, Houston, TX, USA

**Background**

Finding and bringing into effective antiretroviral treatment individuals with undiagnosed HIV infection is required to limit and reduce the burden of HIV. Where to effectively invest limited resources to optimise positive results is an evolving question.

**Methods**

Attempts to link patients to HIV care are reviewed for patients newly diagnosed with HIV infection as a result of universal opt‐out screening made in a major hospital's emergency department in Houston, Texas. Standard opt‐out HIV testing was offered to all presenting adults. An infectious diseases team including linkage workers attempted linkage to HIV care all patients with a new HIV diagnosis.

**Results**

From January to December 2018, 7043 HIV fourth Gen ADVIA HIV Ag/Ab tests were performed. 123 (1.7%) were positive of which 18 (0.25%) were new diagnoses. 94.4% of new HIV diagnoses were successfully disclosed to the patients; 79% of new HIV diagnoses were successfully linked to care with an HIV specialist. The 21% of linkage failures were further investigated to determine characteristics that differentiated them from those who were successfully linked to HIV care. A single finding fully discriminated the groups successfully and unsuccessfully linked to HIV care: those with stable housing were 100% successfully linked to HIV care (Table 1).

**Conclusion**

The importance of finding and bringing into effective HIV treatment individuals with undiagnosed HIV infection was recognised years ago and interventions including emergency room (ER) testing of all presenting patients have been implemented. With the maturation of the ER HIV screening, data are now accumulating on how to make the implementations more effective. In the case of our population in Houston, Texas, the issue of housing of newly diagnosed patients is the most notable constraint on successful HIV intervention.

**Abstract P006‐Table 1. Linkage to care in patients found HIV positive at the Emergency Department**

**Table:** 

	Patients found HIV positive at ER visit n, (%)	Patients found HIV positive at ER visit n, (%)
	New diagnosis, 18 (15%)	Previously diagnosed, 105 (85%)
Contacted by linkage worker	14 (78)	89 (85)
Linkage to HIV care		
Linked	11 (79)
Established	55 (62)
Relinked	29 (32)
Left care	2 (14)	3 (3)
Declined	1 (7)	2 (2)
Housing status		
Stable	12 (86)	75 (84)
Unstable	4 (4)
At risk	1 (7)
Homeless	1 (7)	1 (1)
Hospice	4 (4)
Unknown	5 (6)
Not contactable by linkage	4 (22)	16 (15)
Linkage to HIV care		
Unknown	4 (100)	16 (100)
Housing status		
Unstable	2 (12)
Homeless	4 (25)
Unknown	4 (100)	10 (62)

## P007

Abstract withdrawn

## P008

### Prevalence of dyslipidaemia and characteristics of the lipid profile in patients with HIV infection on highly active antiretroviral therapy in a public Mexican general hospital

**L Ibarra Cobas^1^, B González Flores^2^, M Flores Padilla^3^**

^1^HIV Clinics, Instituto Mexicano del Seguro Social, Saltillo, Mexico. ^2^HIV Clinics, Clínica Especializada Condesa‐Iztapalapa, Mexico City, Mexico. ^3^Internal Medicine, Centro Médico Nacional Siglo XXI, Mexico City, Mexico

**Background**

As far as 2018, 202295 cases of HIV infection have been reported in Mexico [1]. In the general population, it is estimated that the prevalence of dyslipidaemia exceeds 30% [2,3]. People living with HIV (PLWHIV) have increased cardiovascular risk associated with chronic inflammation, immune dysfunction and antivirals [4]. Dyslipidaemia is becoming more prevalent due to the increase in life expectancy and other risk factors in PLWHIV [5]. The purpose of the present study was to evaluate the frequency of dyslipidaemia in Mexican mestizo HIV patients receiving antiretroviral treatment in a Mexican secondary healthcare facility.

**Materials and methods**

A cross‐sectional study was carried out. Clinical records of patients over 18 years of age who had a complete lipid profile and at least six months of continuous antiretroviral treatment were evaluated. Dyslipidaemia was defined as cholesterol level ≥200 mg/dL, triglycerides ≥150 mg/dL, LDL‐C ≥115 mg/dL or HDL‐C <40 mg/dL [6]. Variables are expressed in median and range, bivariate analysis was performed using c2 and *p* < 0.05 was considered statistically significant.

**Results**

A total of 183 patients were included. The median age was 38 years (20 to 72). Out of 183 patients, 88.5% were male, and 80.3% met at least one criterion for dyslipidaemia. The most frequent disorder was hypertriglyceridaemia (60.7%), with a median 214 mg/dL (37 to 713 mg/dL) followed by low HDL‐C levels (49.7%), median 42 mg/dL (25 to 179 mg/dL), hypercholesterolaemia (31.7%), median 183 mg/dL (86 to 381 mg/dL) and high concentrations of LDL‐C (30.6%), median 101 mg/dL (4 to 362 mg/dL). On the bivariate analysis there was a tendency for hypercholesterolaemia in patients receiving protease inhibitors; however, it was not statistically significant in this study.

**Conclusions**

Higher triglyceride values and decrease in HDL‐C were the most commonly encountered disorders. There appears to be an association between the use of protease inhibitors with hypercholesterolaemia; however, it was not statistically significant in this study group. Limitations of the study were the size of the sample and other factors that can cause dyslipidaemia that have not been taken into account. Due to a higher life expectancy of PLWHIV and the cardiovascular risk it represents, it is necessary to implement measurements to identify and control dyslipidaemia and to adjust the antiretroviral drugs associated with this disorder. The results obtained are similar to other series performed in Latin American patients. Currently there are no local guidelines or consensus on the control and treatment of dyslipidaemia in PLWHIV.

**References**

1. Vigilancia Epidemiológica de casos de VIH/SIDA en México. Registro Nacional de Casos de SIDA. Noviembre 2018. DGEPI.

2. Encuesta Nacional de Salud y Nutrición de Medio Camino 2016. Informe final de resultados. INSP.

3. Escobedo‐de la Peña, et al. Dislipidemias en México. Estudio CARMELA. Gaceta Médica de México. 2014;150.

4. Documento de consenso sobre alteraciones metabólicas y riesgo cardiovascular en pacientes con infección por el virus de la inmunodeficiencia humana. Grupo de Estudio sobre Alteraciones Metabólicas  de la Secretaría del Plan Nacional sobre el Sida y del Grupo de Estudio de Sida. Enferm Infecc Microbiol Clin. 2015;33(1):40.e1–40.e16.

5. Tratamiento Antirretroviral del Paciente Adulto con Infección por el VIH. Ciudad de México, Secretaría de Salud, 2017.

6. Diagnóstico y tratamiento de dislipidemias (hipercolesterolemia) en el adulto. México: Secretaría de Salud, 2017.

## HIV and Adolescents, Children and Women

## P009

### Pharmacokinetics, safety and efficacy of bictegravir/emtricitabine/tenofovir alafenamide (B/F/TAF) single‐tablet regimen in HIV‐1‐infected children (6 to <12 years)

**M Cotton^1^, A Liberty^2^, C Rodriguez^3^, K Chokephaibulkit^4^, E Hellstrom^5^, E Natukunda^6^, P Wong^7^, S Majeed^8^, H Graham^9^, R Chirino^10^, F Silva^10^ and C Pikora^9^**

^1^Tygerberg Hospital, Stellenbosch University, Cape Town, South Africa. ^2^Developmental Pathways for Health Research Unit, Chris Hani Baragwanath Academic Hospital, Soweto, South Africa. ^3^Division of Pediatric Infectious Diseases, University of South Florida, Tampa, FL, USA. ^4^Department of Pediatrics, Siriraj Hospital, Mahidol University, Bangkok, Thailand. ^5^Be Part Yoluntu Centre, Western Cape, South Africa. ^6^Department of Pediatrics, Joint Clinical Research Centre, Kampala, Uganda. ^7^Biostatistics, Gilead Sciences Inc., Foster City, CA, USA. ^8^Clinical Pharmacology, Gilead Sciences Inc., Foster City, CA, USA. ^9^Clinical Research, Gilead Sciences Inc., Foster City, CA, USA. ^10^Public Health and Medical Affairs, Gilead Sciences, Mexico City, Mexico

**Background**

Bictegravir (BIC, B), a novel, unboosted integrase strand transfer inhibitor (INSTI) with a high barrier to resistance and low potential for drug interactions, has been coformulated with the recommended NRTI backbone of emtricitabine (F, FTC) and tenofovir alafenamide (TAF) (B/F/TAF) into a once‐daily (QD), single‐tablet regimen (STR). We report pharmacokinetics (PK), safety and efficacy in children who switched from a stable antiretroviral regimen to B/F/TAF.

**Materials and methods**

We conducted a prospective, single‐arm, open‐label, 2‐part, 48‐week (W) clinical trial to evaluate switching to the adult formulation of B/F/TAF (50/200/25 mg) QD in virologically suppressed children (6 to <12 years) weighing ≥25 kg. Intensive PK was evaluated at W2 or W4. PK parameters were compared to B/F/TAF‐treated adults to confirm BIC dose. Adverse events (AE), laboratory tests and HIV‐1 RNA were assessed. We report follow‐up data through W12.

**Results**

Twenty‐five children were enrolled; median (range) age 10 (6 to 11) years, median (range) weight 28.4 (25.0 to 39.0) kg, 52% female, 64% Black, median CD4 count 928 cells/μL. BIC AUCtau was similar, Cmax 77% higher, and Ctau 22% lower in children ≥25 kg than adults. BIC Ctau was well above protein adjusted effective concentration for wild‐type virus (162 ng/mL) in all children. FTC and TAF exposures were within safe and efficacious ranges of adults (Table 1). Through median (Q1, Q3) exposure to study drug of 16.1 (15.9, 17.7) weeks, most common AEs were grade 1 diarrhoea and upper respiratory tract infection (each 16%, 4/25); no other AE occurred in >2 participants. No participant discontinued for an AE. All (100%) had HIV‐1 RNA <50 c/mL at W12; none met criteria for resistance testing.

**Abstract P009‐Table 1. PK parameters of BIC, FTC and TAF after B/F/TAF single‐tablet regimen administration in children and adults**

**Table:** 

	Parameter	Children^a^	Adult^b^	%GLSM Ratio (90% CI)
BIC	AUCtau’ ng × h/mL	121034 (36.4)	102001 (26.9)	116 (104, 130)
	Cmax’ ng/mL	10989 (28.3)	6146 (22.9)	177 (162, 194)
	Ctau’ ng/mL	2367 (78.8)	2610 (35.2)	78.3 (63.4, 96.7)
FTC	AUCtau’ ng × h/mL	17565 (36.9)	12294 (29.2)	142 (127, 159)
	Cmax’ ng/mL	3888 (31.0)	2127 (34.7)	185 (162, 210)
	Ctau’ ng/mL	227 (323)	96.0 (37.4)	95.0 (69.9, 129)
TAF	AUCtau’ ng × h/mL	435 (94.9)	229 (63.0)	175 (136, 226)
	Cmax’ ng/mL	582 (99.9)	277 (62.4)	170 (120, 241)

Parameters are presented as arithmetic mean (%CV); GLSM, geometric least‐squares mean^a^ n = 25 from intensive PK substudy in current cohort of children (6 to <12 years) weighing ≥26 kg^b^; From population PK modelling (BIC,  n = 1193) or pooled intensive PK (FTC and TAF, n = 74 to 77) data from 4 Phase 3 Studies in HIV‐infected adults Statistical comparisons of the PK parameters in children (test) versus adults from Phase 3 studies (reference) were made using GLSM rations and associated 90% confidence intervals (CI) with PK equivalence boundary of 70% to 143%.

**Conclusions**

B/F/TAF maintained virologic suppression and was well tolerated in children through at least 12 weeks. Similar to adults, therapeutic plasma concentrations of all B/F/TAF components of B/F/TAF were achieved. Efficacy and safety in children are consistent with phase 3 B/F/TAF results in adults and adolescents, showing high proportions with viral suppression, excellent tolerability, and no resistance. B/F/TAF may be an important unboosted INSTI, STR option for HIV‐infected children due to its small tablet size, high barrier to resistance and lack of food requirement.

## P010

### Community cohort care: a differentiated model of HIV care for pregnant adolescent girls in Haiti

**T Bell^1^, V Rivera^1,2^, R Riche^1^, Y Cadot^1^, J Bonhomme^1^, Y Macius^1^, M Deschamps^1^, J Pape^1,2^ and M McNairy^1,2^**

^1^Haitian Group for the Study of Kaposi's Sarcoma and Opportunistic Infections (GHESKIO), Port‐au‐Prince, Haiti. ^2^Center for Global Health, Department of Medicine, Weill Cornell Medical College, New York, NY, USA

**Background**

We designed a differentiated model of HIV care to improve retention and adherence among pregnant adolescents in Haiti designed to target stigma, social isolation and lengthy clinic visits.

**Materials and methods**

Community cohort care groups pregnant teens in groups of ˜15 peers who meet monthly in the same group for integrated clinical care and counselling. Groups occur in a community setting. From 1 February 2018 to 31 August 2018, HIV‐infected pregnant adolescents (10 to 24 years) were invited to participate. A PMTCT nurse led each group session including assessment for ART adherence, ANC care, medication dispensation and facilitated peer group counselling. Retention at six months was defined as being alive with a visit between five and seven months; viral suppression was defined as <1000 copies/mL. Retention was compared between cohort care participants and pregnant teens receiving standard care at the PTMCT clinic 1999 to 2014.

**Results**

Twenty‐eight adolescents participated with a median age of 21 years (IQR 20 to 22) and of whom 7% (2/28) were ART naïve, and among those on ART, 58% (15/26) had viral suppression at enrolment. In cohort care, 100% of adolescents initiated ART and 89% (25/28) were retained in care at six months compared to 71% initiating ART and 71% retention in standard care. In cohort care, 4% (1/28) of pregnant teen mothers died and 7% (2/28) were lost. At six months, 85% (23/27) were virally suppressed. A total of 88% (22/25) deliveries were reported born alive among 100% (25/25) teen mothers retained through delivery; 52% of deliveries were assisted by a health professional. The median number of prenatal and postnatal visits per adolescent mother enrolled in cohort care was 3 (IQR 2 to 5) and 5 (IQR 3 to 6) respectively.

**Conclusions**

PTMCT cohort care for HIV‐infected pregnant adolescents plays a critical role in decreasing social isolation, increasing patient knowledge and increasing patient outcomes among Haitian teen mothers.

## P011

### The HIV continuum of care among recently diagnosed adolescents in two large clinics in Mexico City

**M Del Hoyo‐Alvarado^1^, M Hernández‐Leyva^2^, A Piñeirúa‐Menéndez^1^, F Badial‐Hernández^3^, R Leyva‐Flores^4^, R Niño‐Vázquez^5^ and A González‐Rodríguez^3^**

^1^Investigation, Clinica Especializada Condesa Iztapalapa, Mexico City, Mexico. ^2^Investigation, Universidad Nacional Autónoma México, Mexico City, Mexico. ^3^Direction, Clinica Especializada Condesa Iztapalapa, Mexico City, Mexico. ^4^Evaluación de Sistemas y Economía de la Salud, Mexico's National Health Institute, Mexico City, Mexico. ^5^Informática, Clinica Especializada Condesa Iztapalapa, Mexico City, Mexico

**Background**

Worldwide, adolescents and young adults account for 30% of new HIV infections [1]. Furthermore, adolescents living with HIV (ALWH) have worse clinical outcomes [2]. Little is known regarding the HIV epidemic among adolescents in our country. The aim of this study was to describe the continuum of care of recently diagnosed adolescents in two large HIV clinics in Mexico City.

**Methods**

Transversal analysis, nested in a dynamic adolescent cohort starting on 1 December 2017. Data were obtained from the counselling area, medical files and the national antiretroviral surveillance system (SALVAR). SALVAR includes all patients in care at any facility linked to the Ministry of Health. All individuals born after 1 December 1997 and over 14 years old were included.

**Results**

From 1 December 2017 to 31 October 2018, 3536 adolescents were tested for HIV at the clinics. 211 (5.9%) had a positive HIV result. Proportions varied widely between genders: 9.5% of males (199/2073), 3% of transgender women (3/100) and 0.66% of women (9/1363) tested positive. 16% of those who were HIV positive were coinfected with syphilis. The mean CD4+ cell count at diagnosis was 406 cells/mm^3^. 14.7% of the sample had less than 200 CD4+ cell counts at diagnosis. One hundred and twelve (53.1%) adolescents were linked to care through SALVAR; either in Mexico City or other states. Forty‐five (21.3%) were referred to other social security programmes and 53 (25.1%) were lost to follow‐up (LTFU) before linkage to care. Of those who were LTFU, we were able to record data from ten patients, nine males and one female. Their mean age was 18.8 years; regarding their socio demographic characteristics, 80% of those who were LTFU had monthly family incomes under 200 USD, 60% of them were full‐time employees and were born outside Mexico City. Of those who were linked to HIV care through SALVAR, 11 (9.7%) were LTFU after linkage to care. Of 102 currently linked to care patients, 92 (90%) are receiving ART; 65.2% on NNRTI and 33% on integrase inhibitors regimens. Of those on ART and currently in care, 72 (70.6%) are in virological suppression.

**Conclusions**

Adolescents living with HIV remain an important challenge in terms of the HIV continuum. We report a high proportion of LTFU previous to linkage to care, similar to what has been reported in other countries. Specific measures to improve linkage and retention in care among ALWH are urgently needed in our country.

**References**

1. United Nations Programme on HIV‐AIDS (UNAIDS). Databook 2017 [Internet] Available from: http://www.unaids.org/sites/default/files/media_asset/20170720_Data_book_2017_en.pdf

2. Zanoni B, Mayer K. The Adolescent and Young Adult HIV Cascade of Care in the United States: Exaggerated Health Disparities. AIDS Patient Care STDS. 2014;28(3):128–35.

## P012

### Barriers to access HIV‐healthcare services for women living with HIV in Mexico

**I Ramírez Rodríguez^1^, I López Coria^2^, P Belaunzarán‐Zamudio^1^, N Lombardini Vega^2^, J Mejía‐Castrejón^1^, A Piñeirúa‐Menéndez^3^, A Amuchástegui^4^, on behalf of Women Companions in HIV, Yantzin**

^1^Departamento de Infectología, Instituto Nacional de Ciencias Médicas y Nutrición Salvador Zubirán, Mexico City, Mexico. ^2^Mujeres Acompañantes Pares en VIH, Yantzin, Mexico City, Mexico. ^3^Unidad Iztapalapa, Clínica Especializada Condesa, Mexico City, Mexico. ^4^Departamento de Educación y Comunicación, Universidad Autónoma Metropolitana, Mexico City, Mexico

**Background**

Women receiving care for HIV infection in Mexico disengage from medical services, and interrupt treatment more frequently than men, but little is known about the barriers for women to access clinical services, continue in care, and adhere to ART. We describe potential barriers to access HIV‐care services among women receiving care in Mexico; and compare characteristics of women that interrupted ART with those that never did.

**Methods**

A cross‐sectional study was conducted. We enrolled women in care in three centres located in geographically and culturally diverse regions (Mexico City, Cuernavaca and Oaxaca) during 2018. We collected data on individual, family, socio‐economic, physical and gender‐role characteristics, and compared these between those that self‐reported interrupting ART with those never withdrawing ART.

**Results**

We interviewed 164 women (85 in Mexico City, 41 in Oaxaca and 38 in Cuernavaca). The median age was 43 years (range 12 to 87) and most (n = 135, 85%) had been diagnosed before 2013. Women in Oaxaca were younger (32, 12 to 72 years) and more frequently diagnosed in 2017 to 2018 (31/41, 76%). Most women were receiving ART (135/164, 82%). A higher proportion were housewives (68% in Oaxaca and 46% elsewhere). Only 45 (24%) live in a household with a monthly income higher than 200 USD, and in Oaxaca, most women depended on their partners´ income (78% vs. 28%). A higher proportion of women in Oaxaca had children (85% vs. 68%); live more than 4 hours away from their clinic (56% vs. 18%) and spent more to get to the clinic (54 USD vs. 14 USD each visit). The frequency of self‐reported treatment interruption was similar across centres (20%). The most frequent cited reasons for treatment interruption were: adverse effects (32%) and related to depression (26%). Women that interrupted treatment were younger (44 vs. 41 years), and more frequently single (42% vs. 25%). They had a lower education level (79% had <9 years of school vs. 54%), and more likely to have a child (84.2% vs. 63%), or a child with HIV (25% vs. 12%). They were also more likely to be economically dependent from their partners (37% vs. 26%) or the single contributor to family income (32% vs. 17%) and lived outside the urban area where they receive care (60% vs. 45%).

**Conclusions**

In this small sample of women receiving care for HIV infection in three different regions in Mexico, we observed that a high proportion of women self‐report treatment interruptions. A diverse set of individual (age, education), family situation (partnerships status), socio‐economic (family and individual income), gender roles (having children, being economically dependent) and geographic factors (distance) appear to negatively impact the ability of women to adhere to ART over prolonged periods of time.

## P013

### Effectiveness of a protocol for reduction of HIV vertical transmission in Colombia

**K Romero^1^, K Paez^1^, H Paez Ardila^2^and O Sussman^2^**

^1^Asistencia Cientiica de Alta Complejidad, Bogotá, Colombia. ^2^Infectious Diseases, Asistencia Cientiica de Alta Complejidad, Bogotá, Colombia

**Background**

Preventing vertical HIV transmission is one of the major goals in HIV programmes around the world. This study was made to evaluate the effectiveness of the World Health Organization (WHO) protocol for prevention of vertical HIV transmission.

**Materials and methods**

A retrospective descriptive analysis of a cohort of pregnant women diagnosed with HIV infection who attended an HIV programme located in Bogotá Colombia between January 2009 and December 2018 is presented. The primary outcome was perinatal HIV transmission, other variables in study were: gestational age, current and previous antiretroviral (ARV) regimens, viral load of newborn, initial and last HIV viral load plus CD4 count.

**Results**

Data from 152 pregnant women were recollected, although 14 patients were excluded by incomplete data; the majority were diagnosed during first trimester of gestation (24), followed by 41 and 21 diagnosed in second and third trimester respectively. The most preferred ARV regimen was: zidovudine/lamivudine associated with lopinavir/ritonavir. Raltegravir was used in high‐risk cases (third trimester at diagnosis, recent infection; Figure 1). There were no serious adverse effects related to ARV. In the newborn group, all received prophylaxis: 117 of them with zidovudine for 42 days and less than 10% received dual therapy with zidovudine plus nevirapine. The mean HIV viral load at the beginning of treatment in the mothers was 16727 copies/mL, and pre‐cesarea the mean viral load was 45.6 copies/mL. The newborn's follow‐up indicate the absence of HIV perinatal infection in all of them.

**Conclusions**

For the mothers, ARV was safe and effective. WHO protocol guarantees the effectiveness in terms of reducing HIV vertical transmission.

Abstract P013‐Figure 1. Preferred third agent.

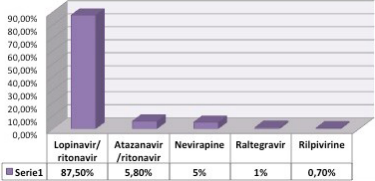

## P014

### Experiences and narratives of HIV women victims of intimate partner violence

**L Arevalo**

HIV Program, Centro de Expertos para Atencion Integral CEPAIN IPS, Bogotá, Colombia

**Introduction**

In the context of violence in HIV, consequences are implicated in all areas for women, which include the loss of social support, social rejection, abandonment and violation of confidentiality. This descriptive exploratory qualitative study focused on the perceptions of HIV women, who have been victims of intimate partner violence to apprehend about the phenomenon and its clarification from their experience.

**Materials and methods**

Participants were women attended in an HIV care centre in Bogotá, and identified as victims of intimate partner violence; group interviews were conducted with a number of two to four people, a total of thirteen women participated. In the analysis of the obtained data, units, categories and patterns were formed, in order to explain contexts, situations, facts and phenomena that establish links between categories, and the relationships between them.

**Results**

It was found manifestations mainly of physical violence, the majority of women interviewed relate situations that have different triggers but all end in a forceful imposition of their partners in blows, or the use of forceful objects. It is followed by verbal violence and psychological violence, which leads to an intense and continuous humiliation, threats of violence, control, disapproval, contempt and/or threat of abandonment. In the moment that they were asked if they considered that the diagnosis of HIV had been a factor in the occurrence of intimate partner violence, only in some cases it was explicitly recognised that HIV increased various types of violence. However, although the majority of women established that there was no direct relationship of partner violence with the diagnosis, in the development of their stories some women narrated situations of violence than corresponded to a correlation with living with the infection. The data were segmented by group of informants and subjects, the process of generating categories was mainly abductive, considering prior categories constructed in the light of the referential framework and at the same time an inductive analysis based on the categories that were emerging from the interviews (Figure 1).

**Conclusions**

Intimate partner violence has multiple causes such as cultural, social, personal, and relationship factors; if personal variables can be intervened, it is possible to break the circle of violence established by disparate power relations that still persist in our society. The accompaniment that we can give as health personnel is essential to guide and give tools to women and empower them when they are victims of violence.

Abstract P014‐Figure 1. Analysis categories.

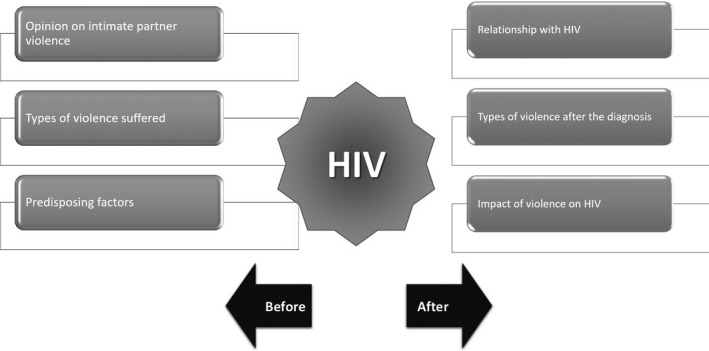

## HIV and Hepatitis Co‐Infection, and Tuberculosis

## P015

### Safety and efficacy of dolutegravir‐based ART in TB/HIV coinfected adults at week 48

**O Sued^1^, K Dooley^2^, R Kaplan^3^, N Mwelase^4^, B Grinsztejn^5^, E Ticona^6^, M Lacerda^7^, E Belonosova^8^, M Ait‐Khaled^9^, K Angelis^10^, D Brown^11^, R Singh^12^, C Talarico^13^, A Tenorio^13^, M Keegan^14^ and M Aboud^15^**

^1^Infectious Diseases, Fundación Huesped, Buenos Aires, Argentina. ^2^Pharmacology, Johns Hopkins University School of Medicine, Baltimore, MD, USA. ^3^Infectious Disease, Desmond Tutu HIV Foundation, Cape Town, South Africa. ^4^Clinical HIV Research Unit, Johannesburg, South Africa. ^5^Clinical Trials, Instituto de Pesquisa Clínica Evandro Chagas FIOCRUZ, Rio de Janeiro, Brazil. ^6^Infectious Diseases, Hospital Dos de Mayo, Lima, Peru. ^7^Infectious Diseases, Fiocruz/Tropical Medicine Foundation Dr Heitor, Vieira Dourado, Manaus, Brazil. ^8^Regional Center for Prevention and Treatment of AIDS and Infectious Diseases, Moscow, Russian Federation. ^9^Virology, ViiV Healthcare, Brentford, United Kingdom. ^10^Statistics, GlaxoSmithKline, Stockley Park, United Kingdom. ^11^ViiV Healthcare, Abbotsford, Australia. ^12^Clinical Pharmacology Modeling and Simulation, ViiV Healthcare, Upper Merion, PA, USA. ^13^Clinical Development, ViiV Healthcare, Research Triangle Park, NC, USA. ^14^Clinical Investigation, ViiV Healthcare, Brentford, United Kingdom. ^15^Medical Affairs, ViiV Healthcare, Brentford, United Kingdom

**Background**

Concurrent treatment of tuberculosis (TB) and HIV is challenging owing to drug interactions, overlapping toxicities and immune reconstitution inflammatory syndrome (IRIS). The efficacy and safety of dolutegravir (DTG) in adults with HIV/TB coinfection were assessed.

**Materials and methods**

INSPIRING (NCT02178592) is a phase 3b, non‐comparative, active control, randomised, open‐label study in HIV‐1‐infected adults’ naïve to antiretroviral therapy (CD4+ 350 cells/µL) with drug‐sensitive TB. Participants on rifampicin‐based TB treatment ≤8 weeks were randomised (3:2) to receive DTG (50 mg twice daily during and two‐week post‐TB therapy, followed by 50 mg once daily) or efavirenz (EFV; 600 mg once daily), with two nucleoside reverse transcriptase inhibitors (NRTIs) for 52 weeks. The week 48 primary endpoint was the proportion of DTG participants with plasma HIV‐1‐RNA <50 c/mL (responders) using the FDA Snapshot algorithm (intent‐to‐treat–exposed (ITT‐E) population). An independent committee adjudicated IRIS episodes. The study was not powered to show a difference between arms; no formal statistical hypothesis was tested.

**Results**

Participants were randomised to DTG (n = 69) or EFV (n = 44). The median baseline HIV‐1‐RNA and CD4+ counts were 5.10 log10 c/mL and 208 cells/µL for DTG and 5.24 log10 c/mL and 202 cells/µL for EFV. The proportions of week 48 responders (ITT‐E) were 52/69 (75%; 95% confidence interval (CI): 65 to 86) for DTG and 36/44 (82%; 95% CI: 70 to 93) for EFV. The DTG non‐response rate was primarily driven by non–treatment‐related discontinuations: 11 participants (16%) for DTG and 3 (7%) for EFV discontinued due to non–treatment‐related reasons while suppressed (mainly loss to follow‐up). There were two protocol‐defined virologic failures (PDVFs) and no treatment‐emergent resistance‐associated mutations (RAMs) for DTG and 1 PDVF in EFV with NRTI and non‐NRTI RAMs. Week 48 median CD4+ increases were 220 cells/µL (interquartile range (IQR): 111, 271) for DTG and 190 cells/µL (IQR: 104, 252) for EFV. TB treatment success was 61/69 (88%) and 39/44 (89%) in DTG and EFV respectively. Two EFV participants discontinued due to adverse events. TB‐associated IRIS rates were low (DTG, n = 4 (6%); EFV, n = 4 (9%)). No participants discontinued due to IRIS or liver events. Median DTG trough concentrations during twice‐daily dosing with rifampicin were similar to that with once‐daily DTG without rifampicin.

**Conclusion**

DTG is effective and well tolerated in HIV/TB coinfected adults receiving rifampicin‐based TB treatment.

## P016

### Phylogenetic distribution of hepatitis B genotypes in individuals coinfected with HIV in Mesoamerica

**M Pérez‐García^1^, C García‐Morales^1^, D Tapia‐Trejo^1^, J Martínez‐Mandiche^2^, J Pascale^2^, S Avila‐Rios^1^, G Reyes‐Terán^1^, A Martínez^2^**

^1^National Institute of Respiratory Diseases, Centre for Research in Infectious Diseases, Mexico City, Mexico. ^2^Genomics and Proteomics, Gorgas Memorial Institute for Health Studies, Panama City, Panama

**Background**

Globally, 2.7 million people are coinfected with HIV and HBV. Little is known regarding the prevalence and genotype distribution of HBV in HIV‐infected subjects in the Mesoamerican region. As the HBV epidemic evolves, it is important to understand the dynamics of HIV/HBV coinfection in the region. In this study, we analyse HBV prevalence, genotype distribution and treatment resistance prevalence in six countries of the Mesoamerican region.

**Materials and methods**

HIV‐infected antiretroviral treatment‐naïve persons were enrolled after written informed consent, as part of a multicentre study evaluating HIV pretreatment drug resistance and HBV coinfection in Mesoamerica. Participants were selected by convenience sampling from key HIV clinics and research centres in the region. Serology for HBsAg was performed for all participants and HBV was genotyped using an in‐house amplification method in those testing positive. HBV sequences were reviewed for quality and a consensus was generated for each participant. Treatment resistance was evaluated with a web‐based application. For genotyping consensus, sequences for each participant were compared phylogenetically to reference HBV sequences from NCBI using MEGA, PhyML and BEAST.

**Results**

In total, 8503 patients were surveyed; 441 (5.19 %) were HBsAg positive. HBV prevalence by country was: Belize 8.7% (n = 139), Panama 6.5% (n = 611), Mexico 6.2% (n = 4111), Honduras 5.0% (n = 1489), Nicaragua 3.6% (n = 392) and Guatemala 2.6% (n = 1761). The median age was 33.5 years, and the most prevalent risk factor was heterosexual transmission (32.7% of all cases), followed by MSM (23.5%), and bisexual (4.9%). Genotype H was identified in 40.6% of amplified samples, followed by genotypes F (26.2%), A (26.2%), G (6.4%) and D (0.5%). Genotype A was the most frequent genotype in Belize and Panama; genotypes H and F in Guatemala and Mexico; Honduras and Nicaragua had equal distribution of the three genotypes. Phylogenetic analysis showed evidence of region‐specific clades, suggesting that subgenotypes may evolve as the virus migrates between countries. Major drug mutations to lamivudine and telbivudine were observed in 17 (8%) of the sequences, and to entecavir in 19 (9%).

**Conclusions**

Overall, HBV coinfection in persons living with HIV in Mesoamerica was under the global prevalence of 7.4% (except for Belize), but over 4%, suggesting middle to high prevalence in the region. Sexual transmission, including heterosexual, remains the leading risk factor for coinfection, warranting focused interventions. Overall, drug resistance prevalence approached 10% for most drugs, underscoring the importance of baseline drug resistance tests and warranting continuous surveillance.

## P017

### Direct‐acting antivirals in HIV/HCV‐coinfected patients from an ambulatory care centre in Buenos Aires, Argentina: real‐life efficacy and safety

**M Laurido, I Cassetti, P Rodriguez Iantorno**

Infectious Diseases, Helios Salud, Buenos Aires, Argentina

**Background**

Several reports from randomised clinical trials and real‐life settings have shown that direct‐acting antiviral (DAA) therapy for chronic HCV infection in HIV/HCV‐coinfected patients yield high response rates in both scenarios. We evaluated the efficacy and safety of these regimens in a private and ambulatory clinical facility.

**Materials and methods**

Retrospective study assessing all HIV/HCV‐coinfected patients underwent HCV treatment with a DAA regimen in routine clinical practice from an ambulatory care centre in Buenos Aires, Argentina, between January 2016 and September 2018. Demographic features, liver fibrosis status, DAA use, adverse events, and sustained virological response 12 weeks after the end of therapy (SVR12) were included in an *ad hoc* data base to be analysed.

**Results**

One hundred and three HIV/HCV‐coinfected patients were enrolled in the study (male 70%; median age (range): 48 years (35 to 74)). All patients were on antiretroviral treatment, 81.5% of them had undetectable HIV‐1 viral load, and 99% had <200 copies/mL. The median CD4 count (range) was 672/mm^3^ (48 to 1276), HCV genotype 1: 89%, cirrhosis: 43.9%, pegylated interferon and ribavirin experienced 38.8%. In terms of DAA use, 53.4% received daclatasvir + sofosbuvir, 40.8% ledipasvir/sofosbuvir, 2.9% paritaprevir/ritonavir/ombitasvir + dasabuvir, 1.9% elbasvir/grazoprevir and 1% sofosbuvir/velpatasvir. Out of 103 patients, 88 ended their DAA therapy and 83 of them reached the time to perform the HCV viral load 12 weeks after the end of therapy. The SVR12 was 98.8% (95% CI: 93.5 to 99.8). Four patients reported grade 1/2 adverse events (3.9%) that were mild and transient. Only one patient (0.9%) had a serious adverse event related to ledipasvir/sofosbuvir that led to its discontinuation.

**Conclusions**

Treatment of HIV/HCV‐coinfected patients for chronic HCV infection with different DAA combinations in a real‐life setting led to high rates of SVR12 similar to those previously described in literature, and the occurrence of adverse events was low. Our results should be encouraging to expand the DAA treatments in the HIV/HCV‐coinfected population in order to control HCV infection.

## P018

### Hepatitis B and C co‐infections in HIV‐infected patients in northern Mexico

**E Pérez‐Alba^1^, M Lozano‐Montemayor^1^, E Díaz‐Chuc^2^, R Cázares‐Tamez^2^ and J Ramos‐Jiménez^1^**

^1^Infectious Diseases, Hospital Universitario Dr. José Eleuterio González, Monterrey, Mexico. ^2^Clinical Pathology, Hospital Universitario Dr. José Eleuterio González, Monterrey, Mexico

**Background**

Co‐infection with HIV, hepatitis B and C virus has important implications especially when considering treatment and prognosis. Since the transmission route of these viruses is shared, co‐infection is not uncommon. HIV has been shown to increase the persistence of HCV and HBV and the risk of hepatocellular carcinoma. According to CENSIDA in Nuevo León, our state, 3402 persons live with HIV, but the co‐infection with other viruses has not been studied. In 2018, the Mexican consensus on the treatment of HCV was updated to include for the first time the use of direct‐acting antivirals. Thus, we consider that it has become critically important to establish the local prevalence of these infections for physicians to know the burden of the disease and establish protocols in their clinics to adapt to newly available treatments.

**Materials and methods**

The Hospital Universitario Dr. José Eleuterio González is a 500‐bed teaching hospital in Nuevo León, Mexico. We aimed to report the seroprevalence of HCV antibodies and HBV surface antigen in the population living with and without HIV that receives medical attention at our institution. A retrospective review of medical and electronic charts was done looking for HIV, HCV and HBV diagnosis, especially looking for co‐infections. Demographic information was also obtained including age, year of infection diagnosis, and gender among others.

**Results**

From 1 January 2013 through to 31 December 2018, 888 persons were diagnosed with HIV infection. They were all tested for HBV and HCV. HCVAb was positive in 4.6% (41/888) of the studied population, while HBsAg was positive in 2.3% (21/888). When compared with the total of positive tests, the +HCVAb/+HIVAg‐Ab represented 9.5% (41/431) of all the reactive HCVAb tests. The +HBvsAg/+HIVAg‐Ab on the other hand was 22.8% of the total HBV infections (chronic and acute).

**Conclusions**

We cannot establish the prevalence since viral loads to confirm infections and rule out false positive tests have not yet been obtained. Nevertheless, it is very alarming that more than a fifth of the new HBV diagnosis are being done in HIV‐infected people. We realise a bias in our results since all our HIV‐infected patients are being tested both for HCV and HBV. We conclude that the preventive measures for hepatitis should be reinforced including HBV vaccination and early detection of HBV must be sought in HIV new diagnosis.

## P019

### HIV and tuberculosis in incarcerated patients in Mexico City during the 5 years, 2013 to 2018

**H Vargas González^1^, H Gudiño Solorio^2^, M Zghaib Rivero^1^, D Molina Martínez^2^ and D Banderas Lares^2^**

^1^HIV/Prisons Programme, Clínica Especializada Condesa, Mexico City, Mexico. ^2^Internal Medicine, Clínica Especializada Condesa, Mexico City, Mexico

**Background**

Currently, the global leading cause of death among people living with HIV is tuberculosis (TB) [1]. Without appropriate treatment, 28% to 53% is at risk of dying, and this risk increases to 65% to 94% among those who are coinfected with HIV [2].

**Material and methods**

A retrospective cohort study to obtain prevalence, incidence, mortality rate and percentage cure among incarcerated patients living with HIV/TB was performed. We used as a diagnostic method for TB: chest X‐ray, basiloscopy, culture and GeneXpert in expectoration and/or gastric juice. Inclusion criteria: (1) incarcerated, male patients infected with HIV and (2) any type of TB diagnosis within the time period of May 2013 to April 2018. The number of study subjects by time period was as follows: 185 from May 2013 to April 2014, 197 from May 2014 to April 2015, 212 from May 2015 to April 2016, 195 from May 2016 to April 2017 and 197 from May 2017 to April 2018.

**Results**

A prevalence rate of 4.1% (8 cases) was estimated up to 1 April 2013. During the time period of May 2013 to April 2018, 63 cases of TB (51 pulmonary, 9 milliary, 2 ganglionic and 1 testicular) were diagnosed, 3 of which were M. tuberculosis resistant to rifampin. We estimated the following incidence rates according to time period: 10.8% (2013 to 2014); 6.6% (2014 to 2015); 6.1% (2015 to 2016), 5.1% (2016 to 2017) and 3.5% (2017 to 2018). There were 11 deaths directly attributable to TB infection, with an estimated mortality rate for the following time periods of: 2.1% (2013 to 2014); 1.5% (2014 to 2015); 1.4% (2015 to 2016), and 0.5% (2016 to 2017 and 2017 to 2018) (Table 1). Of the total number of patients with HIV and TB: 81% were cured with anti‐TB drugs, 17.4% died and 1.6% were lost in follow‐up while on treatment (Figure 1).

**Conclusions**

Local conditions that are possibly driving the transmission of TB in the centre include: (1) overcrowding, (2) promiscuity, (3) little or no ventilation of the facilities and (4) use of inhalable drugs and sharing of drug paraphernalia. Detection and proper treatment allow for: (1) a better control of TB transmission, (2) an increase in the percentage of cured patients and (3) the decrease in mortality rate.

**Abstract P019‐Table 1. Incidence and Mortality rate in HIV/TB (2013 to 2018)**

**Table:** 

Period	Incidence	Mortality rate
2011	2.4	‐
2012	7	‐
May 2013 to April 2014 (n = 185)	10.8	2.1
May 2014 to April 2015 (n = 197)	6.6	1.5
May 2015 to April 2016 (n = 212)	6.1	1.4
May 2016 to April 2017 (n = 195)	5.1	0.5
May 2017 to April 2018 (n = 197)	3.5	0.5

Abstract P019‐Figure 1. Of the 63 patients, 51 (81%) were cured, 11 (17.4%) died and 1 (1.6%) was lost in treatment antifimic.

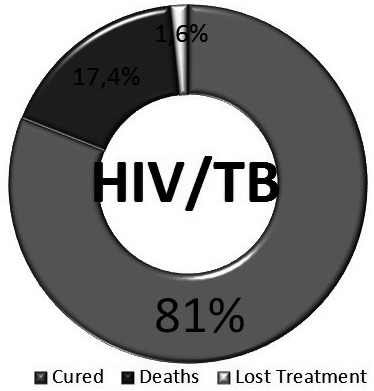

**References**

1. WHO. El control de la tuberculosis en prisiones: Manual para Directores de Programas. 2000. HO/CDS/TB/2000.281.

2. WHO. Global Tuberculosis Report 2015, France. 2015. WHO/HTM/TB/2015.22.

## P020

### Elbasvir/grazoprevir: real‐life experience in hepatitis C‐HIV coinfected patients in Buenos Aires, Argentina

**P Rodriguez Iantorno^1^, M Ferreiro^2^, E Amor^3^, S Paz^4^, M Gordovil^5^, N Porteiro^6^, C Brodersen^7^ and E Gonzalez Ballerga^2^**

^1^Infectious Diseases, Helios Salud, Buenos Aires, Argentina. ^2^Hepatology, Hospital de Clinicas Jose de San Martin, Buenos Aires, Argentina. ^3^ Hepatology, Sanatorio Itoiz, Buenos Aires, Argentina. ^4^Hepatology, Hospital Britanico, Buenos Aires, Argentina. ^5^Infectious Diseases, Hospital Privado de la Comunidad, Mar del Plata, Argentina. ^6^Infectious Diseases, Fundacion IDEAA, Buenos Aires, Argentina. ^7^Hepatology, Hospital Durand, Buenos Aires, Argentina

**Background**

Hepatitis C (HCV) and HIV coinfection is associated with accelerated hepatic fibrosis progression, higher rates of liver decompensation and death when compared to HCV monoinfection. In past years, patients with HCV/HIV coinfection were considered “hard to cure.” Direct‐acting antivirals (DAA) have changed dramatically this landscape. We analysed a cohort of HIV–HCV‐coinfected patients who received specific HCV treatment with elbasvir/grazoprevir (EBR/GZR).

**Methods and aim**

A retrospective analysis of HIV/HCV‐coinfected patients treated with EBR/GZR in several care centres in Buenos Aires, Argentina between February 2017 and August 2018 was carried out to assess the outcomes of EBR/GZR in coinfected patients in a real‐life setting.

**Results**

Twelve coinfected patients were treated during the study period: 11/12 were male. The average age was 50 years. 7/12 had a genotype (G) 1 infection (G1b: 4/7); 3/12 had a G4 and 2/12 had a G3 infection. Only 25% presented extra hepatic manifestations. 7/12 had a viral load higher than 800.000 IU/mL, 10/12 were treatment experienced (3/10 had received DAA as a previous regimen). 8/12 had advanced liver fibrosis (4/8 had cirrhosis). Among cirrhotic patients average MELD score was 7 points. 9/12 received Ribavirin (RBV) and all G3 patients received EBR/GZR+ Sofosbuvir (SOF) per label indication. Data regarding HIV viral loads and CD4 count were collected. Neither viral load nor CD4 count had an impact in sustained virologic response (SVR12). All patients were under antiretroviral treatment and all achieved SVR12 and only 1 patient presented mild asthenia.

**Conclusions**

The combination EBR/GZR was effective and safe in a real‐life setting in this cohort of coinfected patients.

## HIV and Vulnerable Populations

## P021

### Venezuela: the perfect storm: resurging epidemics, a broken health system, and lack of reliable data

**M Torres^1^ and A Nieves^2^**

^1^ICASO, Toronto, Canada. ^2^ACCSI & RVG+, Caracas, Venezuela

**Background**

There is an unprecedented humanitarian emergency in Venezuela. In 2018, the GDP plummeted by 17% and inflation rates soared to 1,698,488% (record). Economic collapse and wide spread corruption have made accessing food and life‐saving medicines impossible. The humanitarian crisis in Venezuela was created by the government that refuses to publish epidemiological data and censors and disciplines those who attempt to do so. For the government, the “absence” of data translates into the absence of a crisis. Their widely documented (IACHR, 2018) refusal to accept foreign “aid” (including medications) supports their denial of any crisis, yet clearly constitutes a violation of the basic human rights of their citizens.

**Materials and methods**

To document the humanitarian and health emergency in Venezuela, ICASO and ACCSI have performed quarterly rapid assessments since September 2017. They include desk reviews along with targeted key informant interviews of PLHIV and other diseases, doctors, advocates, academics and UN representatives.

**Results**

The information collected has been used to influence decision‐making processes of regional and international organisations (Global Fund, UNAIDS, PAHO, IACH) with respect to responding to the crisis in Venezuela. These reviews clearly demonstrate that Venezuela is experiencing several epidemics simultaneously (HIV, malaria, diphtheria, measles, TB and hepatitis), painting a picture of widespread suffering and death that the international community is now recognising and slowly trying to respond to. For example, the number of AIDS‐related deaths in the country has risen by nearly three quarters since 2011 and the widespread stock out of ARVs has resulted in viral suppression of 7% for HIV (UNAIDS, 2018), heightening fears of widespread resistance to a host of ARVs. In malaria, by 2017, the country had the world's worst malaria performance, having 80% of the estimated malaria‐related deaths in the region of the Americas.

**Conclusions**

Venezuela is a case study of the many ways in which international donor policies, based on widely applied generic metrics and standard data collection methods have failed to address a burgeoning crisis in the country and the region. This, combined with the lack of “official” data and the denial of the crisis by the Venezuelan government have created the perfect storm; a humanitarian crisis punctuated with resurgent epidemics that Latin America has never seen. But NGOs have demonstrated that they can respond, not only by providing direct relief, but by gathering the voice from the ground and rigorously collecting, analysing and sharing data that informs decision‐making.

## P022

### National study: prevalence of HIV, hepatitis B and C, syphilis and tuberculosis in people deprived of liberty in federal prisons in Argentina

**J Sotelo^1^, D Adaszko^2^, A Adaszko^2^, P Angeleri^3^, M Orlando^2^, L Angueira^2^ and R Aquino^2^**

^1^Directorate of STI Hepatitis Viral and TB, Ministry of Health and Social Development of the Nation, Buenos Aires, Argentina. ^2^Directorate of STI, Viral Hepatitis and TB, Ministry of Health and Social Development of the, Buenos Aires, Argentina. ^3^Directorate of Epidemiology, Ministry of Health and Social Development of the, Buenos Aires, Argentina

**Background**

One of the sanitarian policies implemented by the Ministry of Health of Argentina in the last decade was the improvement of the health conditions and access to care for imprisoned people. For this reason, between 2015 and 2017, a nationwide study was conducted to determine the prevalence of HIV, syphilis, hepatitis B and C and tuberculosis in federal prisons in Argentina, within the framework of the “justice with health, health convention to include” [1].

**Materials and methods**

An observational cross‐sectional study was designed based on a representative sample of the universe of 10300 imprisoned people in federal prisons. Extractions were made to study HIV (fourth generation ELISA), HBV (HBsAg and Anti‐HBc), hepatitis C (HCV) and syphilis (VDRL confirmed by TF‐PA). Samples were taken from those who had TB‐related symptoms to perform a baciloscopy. All the people who accepted to know their serology received pre‐ and post‐counselling. A self‐administered survey was conducted on sexual practices, care history and drug use. The fieldwork was carried out in 2016. The study was approved by a bioethics committee and was supported by UNAIDS, PAHO, UNODC and the Federal Penitentiary Service.

**Results**

In total, 2181 blood samples were taken and 2277 surveys were carried out in six federal prisons (89% men, 10% women and 1% transgender). All estimates and prevalence values presented here were adjusted based on prison population structure, through sample weights (taking in account 6 variables). The weighted prevalence of HIV was 2.7% (CI: 2.4% to 3%); syphilis 6.8% (5.8% to 7.7%); positive HBsAg was 0.51% (0.37% to 0.65%); positive Anti‐HBc and negative HBsAg was 6.1% (5.5% to 6.5%); HCV was 3.3% (3% to 3.6%). In one case, baciloscopy was positive, so a TB prevalence was 29.6 × 100000. More than 85% of the people tested received their results [2].

**Conclusions**

The findings demonstrate the high prevalence of the infections studied in the prison population and reinforce the need to develop interventions that facilitate access to prevention, diagnosis and care in this population. Federal prisons have 15% of the total number of people imprisoned in Argentina [3]. It is possible that the prevalence of the infections studied may be higher in the rest of the provincial prisons. With regard to the development of an articulated investigation with the management, the process of preparation and the development of the work of field allowed to weave new nets and to strengthen some that already existed, something that must be sustained in the time.

**References**

1. Sotelo J, Sadi M. (2013). VIH‐sida en Contextos de Encierro. *Respuesta preventivo‐asistencial de la Dirección de Sida y ETS*. Ministerio de Salud de la Nación, Argentina, DSyETS.

2. Adaszko D, Sotelo J, Orlando M, Angeleri P. (2017). Estudio de Prevalencia de VIH, sífilis, hepatitis virales y tuberculosis en personas en contextos de encierro en unidades del Servicio Penitenciario Federal.

3. Boletín sobre VIH‐sida en la Argentina N° 34. Dirección de Sida y ETS. Ministerio de Salud de la Nación. Argentina, 2017.

## P023

### Implementation of the strategy “Sexual Diversity Health Services”: systematisation of actions 2010 to 2015

**J Sotelo, J Recchi, S Weller, M Devoto Córdova, N Linares**

Directorate of STI, Viral Hepatitis and TB and Social Development, Ministry of Health, Buenos Aires, Argentina

**Background**

In 2009, the AIDS Direction (DSyETS) of Argentina made the decision to deepen its preventive care policy towards the population of sexual diversity. A first step was the realisation of a diagnostic investigation in 14 cities with the objective of knowing the experience of gay, transgender and bisexual people in relation to the health system. The conclusions of the study (fear of revealing sexual orientation, mistreatment and lack of training of professionals) prompted the DSyETS – in coordination with UN agencies – to a new project for the creation of Sexual Diversity Health Services (CA) as a strategy to improve the access of groups of sexual diversity to the public health system.

**Materials and methods**

The implementation of the strategy “Sexual Diversity Health Services” was developed between 2010 and 2015 by the DSyETS in 21 CA in coordination with local HIV programmes and civil society organisations (CSOs) [1]. It was observed that hospitals and primary healthcare are ideal places for their implementation because they facilitate referrals to other services [2]. Most of the CAs offered the attention in non‐traditional hours benefiting the target population [3]. The benefits most demanded by the population were: hormone, HIV‐STI test, proctology and health control. The DSyETS made technical and financial support to CSOs and health teams for the creation of CA (Table 1).

**Abstract P023‐Table 1.**

**Table:** 

Province	Municipality	Health effectors	Year of creation
Buenos aires	Mar del Plata	Centro de Salud 1	2010
	San Martín	Hospital Local Alexander Fleming	2012
	Lanús	Hospital Evita	2012
	José C. Paz	Centro de Salud Las Heras	2013
	Tigre	Program Municipality	2013
	Ezeiza	CPF I SPF	2013
	Chivilcoy	CIC	2014
	Morón	Hospital Municipal Lavignole	2014
CABA		Nexo AC	1992
		Hospital Ramos Mejía	2006
		Centro de Salud y Acción Comunitaria 11	2009
		Centro de Salud y Acción Comunitaria 25	2013
		Hospital Fernández	2013
		Hospital de Clínicas	1992
Santa Fe	Rosario	Centro de Salud Martin	2006
	Rosario	Hospital Centenario	2012
San Juan	San Juan	Hospital Dr. Guillermo Rawson	2010
	Rawson	Centro de Adiestramiento René Favaloro	2010
	Pocitos	Hospital Dr. Federico Cantoni	2014
	Chimba	Centro de Salud Báez Laspiur	2014
San Luis	Villa Mercedes	Policlínico Regional Juan Domingo Perón	2013

**Results**

The promotion activities of the CA are fundamental for its installation and it is highly recommended to be carried out by members of the collectives, since they know the circuits and codes of the potential beneficiaries [2]. Many CAs have achieved their sustainability thanks to political decisions made by local authorities, promoting changes in the schedule of professionals and including rented promoters. Over a total of 5509 people seen in the CAs, 2353 were gay men, 3 men who have sex with men, 900 transgender women, 119 transgender men, 467 lesbian women, 1465 heterosexual (both men and women) and 202 sexual workers (non‐transgender).

**Conclusions**

By 2018, Argentina has 44 CAs. The promulgation of the Gender Identity National Law (26.743) had a strong impact on people's access to health resources and, in this sense, there is much to be done, being the health sector with the main responsibility for generating this opening and new offers [2].

**References**

1. Boletín sobre VIH‐sida en la Argentina No 34. Dirección de Sida y ETS. Ministerio de Salud de la Nación. Argentina, 2017.

2. Consultorios amigables: n primer paso en la atención de las personas de la diversidad sexual.Sistematización de servicios de salud impulsados por la Dirección de Sida, ETS, Hepatitis y TBC (2010 to 2015).

3. Consultorios amigables: guía para su implementación. PNUD. Argentina, 2013.

## P024

### Cascade of HIV care in prisons in Mexico City and mortality rates during the 5 years, 2014 to 2018

**H Vargas González^1^, H Gudiño Solorio^2^, M Zghaib Rivero^1^, D Molina Martínez^2^ and D Banderas Lares^2^**

^1^HIV/Prisons Programme, Clínica Especializada Condesa, Mexico City, Mexico. ^2^Internal Medicine, Clínica Especializada Condesa, Mexico City, Mexico

**Background**

In Mexico City, approximately 30,000 men are imprisoned, distributed throughout eight centres. Prison inmates diagnosed with HIV infection are transferred to Santa Martha Penitentiary, where they are offered specialised medical care and antiretroviral treatment (ART).

**Objectives**

To construct and describe the cascade of care in patients deprived of their liberty living with HIV (PDLLHIV), and to estimate the annual mortality rates during 5 years (2014 to 2108).

**Material and methods**

Dynamic retrospective and descriptive cohort study. Data from 2014 to 2018 of the ARV Administration and Logistics System (SALVAR) were used, obtaining the annual total number and percentage of PDLLHIV and each component of the cascade of care: diagnosed, linked, retained in ART and virologically suppressed (VL < 40). Mortality rates were calculated for each year, using the annual record of death certificates of PDLLHIV from all Mexico City prisons. Inclusion criteria: male PDLLHIV over 18 years old in Mexico City, during 2014 to 2018.

**Results**

The PDLLVIH care cascade was built annually for 5 years, from an estimated prevalence of 1% [1], with a sample size (N) of between 300 and 350 patients for each year. An increase in the percentage of patients diagnosed throughout the 5 years was observed, from 58.8% in 2014 to 71.3% in 2018. The highest increase was observed in patients in virological control, from 37.1% to 59.7% in said period of time (Figure 1). Mortality rate decreased progressively, with an N of between 206 and 216 patients per year, being 4.8% (10 patients) in 2014, 2.3% (5) in 2015, 1.9% (4) in 2016, 1.4% (3) in 2017 and 2018, 0.47% (1).

**Conclusions**

Our results suggest that the Mexico City HIV Prison Programme has a positive effect on each one of the components of the care cascade, including the consequent virological control in most PDLLHIV, in addition to decreasing mortality due to ART initiation and maintenance. Further strengthen in diagnosis and adherence to ART is necessary to achieve the UNAIDS 90‐90‐90 target. This programme has proven to be effective and can be replicated in different prison systems in other states and countries, but not in the general population.

Abstract P024‐Figure 1. Cascade of HIV care in prisons in Mexico City (2014 to 2018).

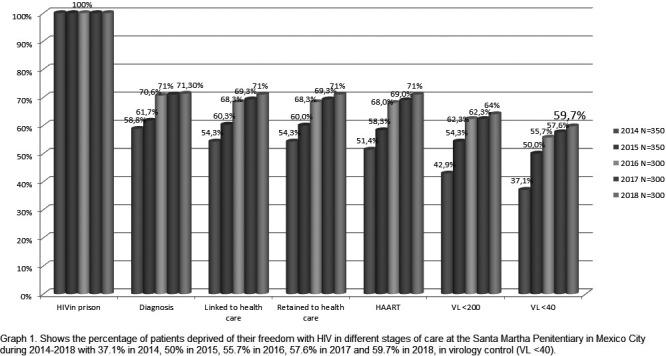

**Reference**

1. Gras N, Badial F, González A. VIH/SIDA y derechos humanos en los centros de reclusión. Revista de derechos humanos dfensor. Salud pública, Número 8. Agosto 2013, pp. 13–21.

## P025

### Peer support for people with recently diagnosed HIV infection improves adherence in the first year after the diagnosis

**E Bottaro^1^, L Faraone^2^, A Soncini^2^, M Pasarón^3^, N Roussilian^2^, M Cascasi^4^, A Otreras^1^, M De Bernardi^1^, S Rodríguez^2^, S Errea^1^, D D'Alessandro^1^, M Rodriguez Brieschke^1^, J Caccavo^1^, B Lauge^1^ and P Scapellato^1^**

^1^Infectious Diseases, Hospital General de Agudos D F Santojanni, Buenos Aires, Argentina. ^2^Social Service, Hospital General de Agudos D F Santojanni, Buenos Aires, Argentina. ^3^Mental Health, Hospital General de Agudos D F Santojanni, Buenos Aires, Argentina. ^4^Pharmacy, Hospital General de Agudos D F Santojanni, Buenos Aires, Argentina

**Background**

There are many challenges to link and retain in care people living with HIV (PLHIV). In order to promote adherence, several strategies were applied. One of those is the peer support. A peer is a PLHIV trained to help others who share their HIV status, to overcome barriers that arise in medical care. With that goal, we created, “Positivos para Positivos” (PPP), a peers group for supporting PLHIV with recent diagnosis of HIV infection. Our objective was to compare adherence and laboratory parameters in recently HIV‐diagnosed PLHIV as they were accompanied or not by members of PPP.

**Material and methods**

A team of physicians, social workers and psychologists selected adherent PLHIV with undetectable plasmatic HIV viral load (VL), assisted in our centre, and offered them to integrate PPP. Those who accepted were trained by the team. Recent diagnosis was defined when the first positive test was performed ≤6 months before the first consultation. Each PLHIV with recent diagnosis were offered contact with a member of PPP. If they agreed, two PPP members were designated for their accompaniment. Recently diagnosed PLHIV with at least one appointment with a PPP member constituted the “study group.” The “control group” was constituted by PLHIV who refused to contact and by those who were not offered (for any reason). Medical care to both groups was identical. The analysed variables were: deaths in every group; proportion of PLHIV with optimal clinical control (≥3 visits to the office during follow‐up); proportion of PLHIV who started HAART; pharmacy refill rate; unstructured HAART interruptions (≥3 consecutive months without pharmacy refill); CD4 evolution; proportion of PLHIV lost to follow‐up (≥6 consecutive months without contact with the healthcare system); proportion of PLHIV with good laboratory control (≥1 blood collection during follow‐up); proportion of PLHIV who achieved undetectable VL; proportion of PLHIV who increased >50 CD4/mm^3^ at the end of the follow‐up. Categorical variables were analysed by 2 × 2 tables and chi‐square (EpiInfo 7.2.2.6).

**Results**

In total, 127 PLHIV met the inclusion criteria. The “study group” had more advanced disease. Otherwise, both groups were similar. The results are shown in Table 1.

**Conclusion**

Peer support had a positive impact on adherence parameters of recently diagnosed PLHIV during the first year after HIV diagnosis. The influence on laboratory parameters was weak.

**Abstract P025‐Table 1. Comparison of studied parameters in recently HIV‐diagnosed PLHIV according to whether they received (study group) or not (control group) peer support**

**Table:** 

Variable	Study group (n = 32)	Control group (n = 95)	RR (95% CI)	*p* value
PLVIH died (all of them died before three months of follow‐up); n/n (%)	1/32 (3)	2/95 (2.1)	0.99 (0.92 to 1.06)	1
Optimal clinical control; n/n (%)^a^	30/31 (96.8)	70/93 (75.3)	1.29 (1.13 to 1.47)	0.008
PLVIH who started HAART; n/n (%)	32/32 (100)	79/95 (83.2)	1.2 (1.09 to 1.32)	0.01
Refill rate >90%; n/n (%)^a^	31/31 (100)	27/78 (34.6)	1.96 (1.33 to 2.89)	0.002
Unstructured HAART interruption; n/n (%)	4/31 (12.9)	29/78 (37.2)	0.34 (0.13 to 0.91)	0.02
PLVIH with ≥1 blood collection n/n (%)^b^	26/31 (83.9)	54/78 (69.2)	1.21 (0.98 to 1.5)	0.12
PLVIH who achieved CV <50; n/n with available data (%)^c^	26/26 (100)	45/54 (83.3)	1.2 (1.06 to 1.35)	0.05
PLVIH with increase >50 CD4/mm^3^; n/n with available date (%)^c^	19/26 (73.1)	38/52 (72.5)	1 (0.75 to 1.3)	1
PLVIH lost to follow‐up; n (%)	2/31 (6.5)	23/93 (24.7)	0.26 (0.07 to 1.04)	0.04

^a^Denominator excludes dead patients; ^b^Only PLHIV who started HAART; ^c^Only PLHIV with ≥1 blood collection (in two patients of the control group CD4 was not informed).

## P026

### Pregnant migrant patients in Chile's largest HIV care centre: changes derived from country's migration patterns

**M Rodríguez^1^, D Lizana^1^, M Mercado^2^, M Wolff^3^ and C Cortes^3^**

^1^Infectious Diseases/HIV, Fundación Arriarán, Santiago, Chile. ^2^Infectious Diseases Residency, Universidad de Chile, Santiago, Chile. ^3^Medicina, Universidad de Chile, Santiago, Chile

**Background**

There has been a significant rise in the migrant population in Chile in the last decade. National census reported 1.27% of foreign‐born people in 2002 and 4.35% in 2017. Fundación Arriarán (FA) is the largest HIV/AIDS care centre in Chile. It holds records of 7300 HIV patients, 1143 of them immigrants (15.7%). We describe demographic changes in migrant pregnant patients in FA since its beginning in 1991 (Figure 1).

**Materials and methods**

Retrospective analysis from FA databases.

**Results**

In total, 7337 patients have been to FA since 1991. Eighty‐nine were admitted through HIV screening during pregnancy. 76% (n = 68) were migrants. All of them were admitted after 2008. The median age at admission was 29 years. At admission, the mean CD4 + count was 374.5 cells/mm^3^ and the mean viral load (VL) was 10,985 copies/mL. Pregnant migrants admitted were 1/2008, 1/2013, 6/2014, 2/2015, 10/2016, 26/2017 and 23/2018. In 2008, 100% migrant admissions were from Peru; in 2017, 19% were Peruvian, 69% from Haiti; in 2018, 81% were from Haiti, and none of the admitted patients came from Peru. Current status of foreign‐born pregnant admissions at December 2018 was as follows: 0% deceased, 14.7% lost to follow‐up (LTFU), 1.4% transferred to other sites and 83.8% retained in care at site.

**Conclusions**

Overall, migrant pregnant women vastly surpassed local pregnant women. During the last decade, a significant increase in foreign pregnant admissions was observed. In the last two years an increasing number of them came from Haiti. Most women diagnosed through pregnancy HIV screening were foreign, mostly Haitian. LTFU rate in migrant pregnant population almost triplicates local LTFU (4.5%) and doubles general migrants LTFU rate (8.3%). Increasing and changing migrant population requires specific strategies to reduce care retention gaps. Special consideration should be taken towards those with language and cultural barriers, a new challenge in our country and centre.

Abstract P026‐Figure 1. Migrant pregnancy admissions through the years.

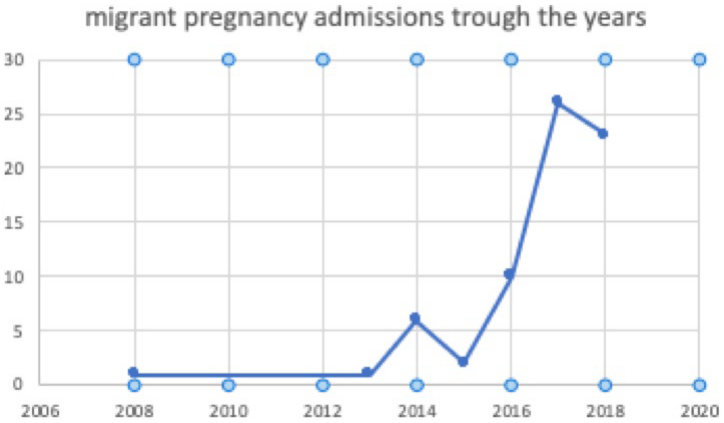

## P027

### Syphilis prevalence among men who have sex with men attending community‐based HIV testing clinic in the city of Guadalajara, Mexico (2016 to 2018)

**G Martinez Vizcaino^1^, P Belaunzarán‐Zamudio^2^ and V Galicia Juarez^3^**

^1^Detection Center, Comite Humanitario de Esfuerzo Compartido Contra el SIDA A.C., Guadalajara, Mexico. ^2^Infectology, Instituto Nacional de Ciencias Médicas y Nutrición Salvador Zubirán, Mexico City, Mexico. ^3^Director, Comite Humanitario de Esfuerzo Compartido Contra el SIDA A.C., Guadalajara, Mexico

**Background**

The HIV epidemic in Mexico is concentrated among men who have sex with (MSM). While increasing syphilis rates among MSM have been documented worldwide, little is known about its frequency in Mexico. We estimated syphilis prevalence in MSM receiving voluntary counselling and testing services in Guadalajara Mexico during 2016 to 2018.

**Methods**

The Humanitarian Committee of Joint Efforts Against AIDS (CHECCOS in Spanish) is a non‐governmental organisation that operates a community‐based clinic providing testing and counselling services for HIV and other sexually transmitted infections (STIs), and individual and group peer‐support activities in Guadalajara and Mexico Cities. Testing is provided free of charge using a fourth Gen EIA rapid testing platform and ONE STEP Syphilis anti‐TP tests. People testing reactive are invited to the peer‐support programme during the process of linkage to care with HIV/STI clinics. People testing reactive to syphilis are provided medical care, treatment and follow‐up. We used anonymised HIV and syphilis test results to estimate syphilis prevalence at the centre and compared prevalence between HIV‐negative and HIV‐positive MSM tested between 2016 and 2018.

**Results**

CHECCOS Community Center offered HIV and syphilis tests to 1398 MSM during this period. There were 198 (14.4%) syphilis‐ and 234 (17%) HIV‐reactive tests. Syphilis prevalence was lower among HIV‐negative MSM (n, 10.1%) than in HIV‐positive (n, 35.5%); (OR = 3.4; 95% CI: 2.7 to 4.2). The mean age of syphilis‐positive MSM was 31.2 years in both groups.

**Conclusions**

We observed a very high prevalence of syphilis among MSM in the city of Guadalajara, Mexico during 2016 to 2018, particularly among those HIV positive. Our results show that CHECCOS Community Center in Guadalajara targets populations at very high‐risk of HIV and other STIs and in need of treatment. This information provides important and unprecedented information that contributes to a deeper understanding of the HIV epidemic in Mexico among MSM and supports the need to expand community‐based services.

## P028

### Characterisation of the migrant population with HIV infection in a public hospital

**C Medina Collado and F Mejia Cordero**

Enfermedades Infecciosas, Hospital Cayetano Heredia, Lima, Peru

**Background**

There has been an increasing number of immigrants to Perú in the last years, especially from Venezuela. The phenomenon of immigration generates a strong impact on the health system [1‐3]. Cayetano Heredia Hospital serves a large number of patients with HIV infection. Started treatment for 2530 patients between 2016 and 2018, 13% of them were immigrants. The objective of this study, is to determine baseline clinical and epidemiological characteristics of the HIV infection in immigrants presenting to a referral HIV public hospital between 2016 and 2018.

**Methods**

This is a descriptive study, epidemiological data about HIV/AIDS in immigrant patients as well as immunological and virological data were used.

**Results**

Treatment was started in 321 immigrant patients with HIV infection between 2016 and 2018, 82% were Venezuelans, and 89% were male. 42% have university studies, 51% are homosexual and 31% are bisexual. 53% of patients had a diagnosis time of five years or less. 25% were symptomatic at the time of diagnosis. 60% was diagnosed in a routine consultation. The route of transmission was sexual in all cases. 25% had AIDS stage at the time of diagnosis in their country of origin. 42% of patients had CD4 greater than 500, 60% of patients had undetectable viral load and 11% were in AIDS stage at the time of entering the Peruvian health system. 69% had received treatment in their country and 18% had a history of having abandoned treatment. 71% of patients who started treatment in the Peruvian health system started with non‐nucleoside analogues.

**Conclusions**

The increase in the immigrant population with HIV infection in Peru is mainly from Venezuela. This increase is mainly of MSM population, with higher education and with good response to antiretroviral treatment. The response capacity of the Peruvian health system must be evaluated to ensure the sustainability of the treatment in this group of patients.

**References**

1. M Fernanda Rodríguez, Marcelo Wolff y Claudia Cortés. Características clínicas y epidemiológicas de la infección por VIH en inmigrantes latinoamericanos versus chilenos: estudio comparativo en un centro de atención de Santiago a partir de registros de 2003‐2013. Revista chilena de infectologia. 2015;32:72–80.

2. Llenas‐Garcíaa J, Rubioa R, Hernando A, et al. Características clínico‐epidemiológicas de los pacientes inmigrantes con infección por el VIH: estudio de 371 casos. Enferm Infecc Microbiol Clin. 2012;30(8):441–51.

3. Bermúdez, María Paz; Castro, Ángel; Madrid, Juan; Buela‐Casal, Gualberto. Análisis de la conducta sexual de adolescentes autóctonos e inmigrantes latinoamericanos en España. Int J Clin Health Psychol. 2010;10(1):89–103.

## P029

Abstract withdrawn

## P030

### Characteristics of hormone use by *travestis* and transgender women of the Brazilian Federal District: a respondent‐driven sampling study

**A Krüger^1^, S Sperandei^2^, X Diáz Bermúdez^3^, E Merchán‐Hamann^3^, N de Araújo^1^, G Casimiro da Silva^1^, A Schwartz Benzaken^4^ and G Fernando Mendes Pereira^1^**

^1^ Department of STI, HIV/AIDS and Viral Hepatitis, Ministry of Health of Brazil, Brasília, Brazil. ^2^ICICT, FIOCRUZ, Rio de Janeiro, Brazil. ^3^PPGSC/FS, Universidade de Brasília, Brasília, Brazil. ^4^Direction, FUAM, Manaus, Brazil

**Background**

*Travestis* and transgender women, in search of signs of corporal femininity, resort to different technologies for body modification, such as hormones [1]. Due to characteristics of frequent access denial to health services, the use of these hormones occurs, mainly, by self‐medication [1]. They are considered a key population due to high HIV prevalence found in Brazil and worldwide [2]. Therefore, discussing other aspects of health, such as access to inputs and healthcare services are part of the concept of HIV combination prevention, under its structural approaches. This study aims to describe the self‐reported prevalence of hormone use by *travestis* and transgender women in the Brazilian Federal District.

**Materials and methods**

A cross‐sectional study with RDS sampling, containing a KAP questionnaire, carried out with *travestis* and transgender women over 18 years of age, with some relationship to the Brazilian Federal District and never having participated in the study. The statistical analysis used 95% confidence intervals. Estimated prevalence used the RDS‐II estimator. All analyses were performed in programme R, version 3.4.4 using the RDS package [3].

**Results**

We analysed information from 201 participants. The study had a young sample, with a median age of 24 years. The overall prevalence of continuous hormone use was 64.5%. The most used formulation was the one that combines oestrogen and progesterone (86.2%), in the injectable (75.1%) and oral (66%) forms respectively. A great part (84%) of the participants got the hormones directly in the pharmacies, without medical prescription. The guidance on use of these medicines came from other *travestis* and transgender women in 41% of the cases. Satisfaction with the use of hormones was high (over 70%), as well as the side‐effects felt (in 63% of cases). Discontinuation of hormone use in the event of side‐effects was the attitude taken by a great part of the respondents (43%).

**Conclusions**

This study demonstrated the reality of great rates of self‐medication indicating poor access to healthcare services, reflected in high rates of side‐effects, discontinuation of use and receipt of technical information only from peers. Related to the structural approaches to HIV combination prevention, these kind of studies about access are very important because they can be considered a proxy to access of these persons, suggesting a lack of access to HIV inputs and healthcare services as well. More studies to confirm this hypothesis are fundamental.

**References**

1. The World Professional Association for Transgender Health. Standards of Care for the Health of Transsexual, Transgender, and Gender Nonconforming People 7th version. The World Professional Association for Transgender Health (WPATH): 2012. 131 p. Available from: https://www.wpath.org/media/cms/Documents/SOC%20v7/SOC%20V7_Portuguese.pdf. [cited 2019 an].

2. Brasil. Ministério da Saúde. Secretaria de Vigilância em Saúde. Departamento de Vigilância, Prevenção e Controle das Infecções Sexualmente Transmissíveis, do HIV/Aids e das Hepatites Virais. Prevenção Combinada do HIV/Bases conceituais para profissionais, trabalhadores (as) e gestores (as) de saúde– Brasília: Ministério da Saúde, 2017.

3. Bastos FI et al. HIV, HCV, HBV, and syphilis among transgender women from Brazil: Assessing Different Methods to Adjust Infection Rates of a Hard‐to‐Reach, Sparse Population. Medicine [Internet] 2018 [cited 2019 Jan] 97:16–24. Available from: https://www.ncbi.nlm.nih.gov/pubmed/29794601.

## P031

Abstract withdrawn

## P032

Abstract withdrawn

## P033

### Limitations of traditional risk functions to predict cardiovascular risk among transgender population with HIV in Buenos Aires, Argentina

**J Ballivian^1^, J Gago^2^, D Costa^3^, I Davolos^3^, A Benchetrit^4^, S Ivalo^1^, G Viloria^1^ and M Losso^1^**

^1^HIV Unit, Hospital Ramos Mejia, Buenos Aires, Argentina. ^2^Programa de Cancer Cervicouterino, National Cancer Institute, Buenos Aires, Argentina. ^3^Servicio de Cardiologia, Hospital de Clinicas Jose de San Martin, Buenos Aires, Argentina. ^4^Infectologia, Hospital Muñiz, Buenos Aires, Argentina

**Background**

The prevalence and incidence of cardiovascular disease (CVD) are expected to increase among people living with HIV as their lifespan improves in South America due to accessibility to new treatment regimes [1]. Transgender women (TW) have unique characteristics, thus, CVD risk factors in this group may differ from general population. The short lifespan of TW in Argentina, estimated around 40 years, constitutes a major challenge to use most of CVD risk scores. We aim to assess the impact of non‐traditional CVD risk factors in TW with the Framingham 30‐year risk score at their first visit to an HIV/STI clinic in Buenos Aires, Argentina.

**Methods**

We performed a cross‐sectional study to analyse non‐traditional CVD risk factors and evaluate their possible association with intermediate/high 30‐year CVD risk among TW at an HIV/STI clinic in Buenos Aires, Argentina, from 2014 to 2017. TW between 20 and 60 years at their first visit to the clinic were included. We evaluated CVD risk assessed with the Framingham 30‐year score as our outcome [2]. As independent variables, we evaluated HIV status, cocaine use and hormone therapy (HT). CVD risk was analysed as a dichotomic variable (low‐risk vs. intermediate/high‐risk). Finally, a logistic regressions analysis was performed to investigate the effect of these non‐traditional CVD risk factors on our outcome.

**Results**

One hundred and eleven TW were eligible for this analysis, 37 (33.3%) were HIV positive, 44 (39.6%) reported use of HT and 52 (46.8%) reported use of inhaled cocaine. The median age was 31 years (IQR 27 to 37). 80 patients (71.43%) were found to have low CVD risk. According to our model, cocaine use and HT use were not significantly associated with high CVD risk (OR 0.92 *p *= 0.853 and OR 0.58 *p* = 0.25 respectively). Moreover, HIV was associated with lower odds of having intermediate/high CVD risk, being these results statistically significant (OR 0.23 *p* = 0.026).

**Conclusion**

Well‐known non‐traditional CVD risk factors such as HIV infection and cocaine use were not associated with increased odds of having high CVD risk. Moreover, patients with HIV seemed to have lower odds of intermediate/high CVD risk according to our analysis. Traditional CVD risk functions are not accurate tools to establish CVD risk among people living with HIV [3]. Our study shows that this risk function might be even less accurate for assessing this outcome among the transgender population in our setting. More studies in this population need to be done in order to find better tools for measuring CVD risk more accurately.

**References**

1. Tunstall‐Pedoe H. Preventing Chronic Diseases. A Vital Investment: WHO Global Report. Geneva: World Health Organization, 2005.

2. Pencina MJ, et al. Predicting the 30‐year risk of cardiovascular disease: the Framingham Heart Study. Circulation. 2009;119(24):3078–84.

3. Eyawo O, et al. Changes in mortality rates and causes of death in a population‐based cohort of persons living with and without HIV from 1996 to 2012. BMC Infect Dis. 2017;17(1):174.

## P034

### HIV‐2 antibody characterisation among transwomen populations living with HIV in the Dominican Republic

**E Reynoso Vanderhorst, R Ibarra, L Tapia and R Paulino‐Ramirez**

Universidad Iberoamericana, Institute for Tropical Medicine and Global Health, Santo Domingo, Dominican Republic

**Background**

HIV‐2 infections predominate in the West African region but the presence of the virus has been identified in other parts of the world [1]. In the Dominican Republic, trans population has an elevated prevalence of HIV infection (3.9% to 6.9%) compared with general population [2]. Not all antiretrovirals developed for HIV‐1 treatment are equally effective in HIV‐2 infection, and limited data exist on the efficacy of new ARV on HIV‐2 [3,4]. The objective of this study was to further characterise the presence of HIV‐2 in transwomen populations living with HIV in the Dominican Republic.

**Methods**

Serological analysis to identify HIV‐2 antibodies by PCR and Multispotin previously confirmed HIV‐positive samples of transwomen volunteers. Socio‐demographic determinants were linked with serological data and statistical analyses were used to assess risk determinants of HIV‐2 infection.

**Results**

A total of 110 participants were evaluated for the presence of HIV‐2 serum antibody. We found the presence of HIV‐2 as a co‐infection with HIV‐1 in 5.45% (n = 6) of participants. Single infection with HIV‐2 was not found. Geographical origin of participants with confirmed co‐infections was located near the border with Haiti. No correlation was found with alcohol consumption and condom use. All positive results were returned and positive cases were enrolled in care for HIV infection in their localities (Table 1).

**Abstract P034‐Table 1. Demographic characteristics of transgender population infected with HIV‐2**

**Table:** 

	HIV‐2 Population (n = 6), n (%)
Sexual preference	
Transexual	2 (33.3)
Bisexual	1 (16.7)
Homosexual	1 (16.7)
Transgender Women	1 (16.7)
Age group	
18 to 24	2 (33.3)
25 to 29	1 (16.7)
30 to 34	3 (50)
Alcohol consumption	
2 to 4 drinks/month	3 (50)
2 to 3 drinks/week	1 (16.7)
4 or more/week	1 (16.7)
Condom use in the last month	
Never	1 (16.7)
Sometimes	2 (33.3)
Always	3 (50)

**Conclusion**

This study identified the presence and circulation of HIV‐2 in transwomen populations in the Dominican Republic. A more systematic screening for HIV‐2 should be considered for management, treatment and prevention among high‐risk populations, and its implications on new diagnosis and immediate treatment. Further evaluations should be considered to evaluate their progression through the continuum of care.

**References**

1. SM. HIV – 2 Infections from a Tertiary Care Hospital in India – A Case Report. Journal of Human Virology & Retrovirology 2017;5. https://doi.org/10.15406/jhvrv.2017.05.00161.

2. Paulino‐Ramírez R, Rodríguez‐Lauzurique M, Santo R. Encuesta de Vigilancia de Comportamiento con Vinculación Serológica en Población Trans de la República Dominicana. CONAVIHSIDA/COIN/UNIBE. 2016. [cited 2019 Jan 25] Available from: http://coin.org.do/wp‐content/uploads/2017/10/INFORME‐ECVS‐TRANS‐2016.pdf.

3. Witvrouw M, Pannecouque C, Switzer WM, Folks TM, De Clercq E, Heneine W. Susceptibility of HIV‐2, SIV and SHIV to various anti‐HIV‐1 compounds: implications for treatment and postexposure prophylaxis. Antivir Ther 2004;9:57–65.

4. HIV‐2 Therapy Guidelines Draft 2017 HIV‐2 Working Group genafor.org/arevir2017/presentation/2/Berzow_HIV‐2 European Guidelines_2017_Draft.pdf.

## P035

### Study of HIV cases registered among prisoners in South Santa Fe, Argentina

**F Biasutti^1^, E Rossi^1^, A Moro^2^, R Huanca^3^, M Lodigiani^3^, E Crochet^3^ and E Careno^1^**

^1^TB/HIV Program, Ministry of Health, Santa Fe, Argentina. ^2^Municipal System of Epidemiology, Ministry of Public Health, Rosario, Argentina. ^3^Ministry of Security, Penitentiary Service of Santa Fe, Santa Fe, Argentina

**Background**

Prisoners used to have disadvantaged life conditions or drug addiction, which leave them in a vulnerable population to different infections, such as HIV. According to WHO, HIV prevalence for prisoners is 15 times higher than in general adult population [1].

**Materials and methods**

This work studied HIV cases notified among prisoners of the six centres of detention of South Santa Fe region (SSF), Argentina; where 4250 people (4145 men and 105 women) were imprisoned during the analysis period (January 2017 to May 2018). SSF medical staff offer HIV screening tests to all the prison population, but prisoners accept to take them only voluntarily. In case the screening test results positive, additional tests, such as CD4 and viral load determinations, are carried out. All patients diagnosed with HIV received the corresponding treatment.

**Results**

During the studied period, the serological condition for HIV of 835 prisoners was known, which represents only a 19.6% of the total population in SSF prisons. Among them: 72 (8.6%) were HIV (+), being men 93.1%; 12 (16.7%) were also infected with hepatitis C virus; a 91.6% were put under treatment; and 175 viral load tests were carried out as a mean of treatment control, which represents an average of 2.4 viral load tests/year/patient. After the treatment, the population with undetectable or low grade viremia reached a 81.8%, which could be considered a good response. 7.6% (5 patients) presented virological failure, and all cases corresponded to under NNRTI treatment (Figure 1).

**Conclusions**

HIV infection among prisoners is a critic health problem which requires a proactive attitude by the medical staff to improve the epidemiological situation in prisons. In SSF, the immediate aim to achieve is being able to carry out a greater number of tests for HIV, which implies an active information campaign to make prisoners aware of the problem and their possibilities of treatment.

Abstract P035‐Figure 1. Distribution of patients on antiretroviral treatment, as a result of the treatment. Penitentiary Service Santa Fe South Region. Period: 01/01/2018 to 31/08/2018 (n = 66). Source: SISA/SVIH (Argentine integrated health information system HIV)

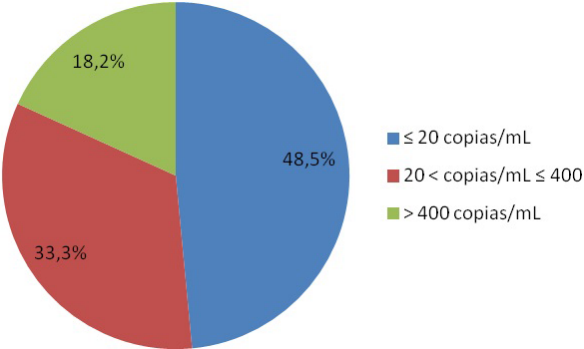

**Reference**

1. People in prisons and other closed settings. WHO, Available from: https://www.who.int/hiv/topics/prisons/en/.

## P036

### A positive prevention programme for HIV‐positive gay men in Colombia: pilot study evaluation

**B Alvarado^1^, J Martínez‐Cajas^2^, H Mueses‐Marín^3^, D Montaño^3^, D Correa^3^, B Adam^4^ and T Hart^5^**

^1^Public Health Sciences, Queens University, Queens, Canada. ^2^Infectious Diseases Division, Queens University, Queens, Canada. ^3^Investigaciones, Corporación de Lucha Contra el Sida, Cali, Colombia. ^4^Sociology, Anthropology, and Criminology, University of Windsor, Toronto, Canada. ^5^Psychology, Ryerson University, Toronto, Canada

**Background**

This study evaluated the effects of an individual counselling intervention that provides information, motivational interviews and behavioural skill building to reduce sexual transmission risk behaviours. The preliminary effects of this intervention, which is named Gay Poz Sex (GPS), were evaluated in terms of acceptability, appropriateness, and the psychosocial and HIV sexual risk behaviour outcomes.

**Method**

Two HIV‐positive peer counsellors administered six 2‐hour counselling sessions to 11 HIV‐positive gay men living in Cali, Colombia. A pre‐post study without control groups was designed to assess effects of the intervention on depression, loneliness, self‐efficacy of condom negotiation and on condom‐less anal sex (CAS). The paired t‐test and McNemar's test were used to assess the effects at the end of the intervention and three months later. Semi‐structured interviews with participants on intervention acceptability, effectiveness and appropriateness were also assessed after three months of the intervention.

**Results**

A total of 7 of the 11 eligible participants finished the GPS intervention and completed the follow‐up. We observed a reduction in CAS (any partner) from 83% at baseline to 46% at the three‐month follow‐up. After three months, a significant increase was found in condom use and negotiation (*p* = 0.01), and there was a decrease in the depression score (*p* = 0.07). Participants perceived the programme to be acceptable and highly appropriate. Favourable responses were mainly related to 1) the relevant nature of information, 2) a chance to discuss sex in a non‐judgemental place, 3) a well‐designed intervention, and 4) helping to make positive changes in their sexual life and decrease risky sexual behaviours (i.e. drug use and the use of Internet dating sites).

**Conclusions**

This work was done to fill an important gap in prevention of HIV transmission and acquisition in gay men who are HIV positive and living in Colombia. The findings provide preliminary evidence that a counselling intervention led by peers may offer an efficient way to concurrently reduce CAS, increase negotiation for condoms, and mental health problems in HIV‐positive gay men. These results favour GPS as an intervention to reduce the transmission of HIV in Colombia.

## P037

### A pilot study of a prevention programme for Latino gay men in Canada: results in terms of effectiveness

**B Alvarado^1^, B Adam^2^, T Hart^3^ and J Martinez‐Cajas^4^**

^1^Public Health Sciences, Queens University, Kingston, Canada. ^2^Ontario HIV Treatment Network, Toronto, Canada. ^3^Department of Psychology, Ryerson University, Toronto, Canada. ^4^Division of Infectious Diseases, Queens University, Kingston, Canada

**Background**

This study piloted the programme Gay Poz Sex (GPS) in order to fill an important gap in preventing HIV transmission and acquisition in gay men of Latino origin living in Canada. GPS is an individual counselling intervention that employs information provision, motivational interviewing, and behavioural skills building to reduce HIV and STI transmission risk behaviours. This paper presents the effects GPS had on the reduction of these behaviours and on psychosocial outcomes for participants.

**Materials and methods**

This pre‐post without control study was designed to assess GPS’ effects on depression, loneliness, the self‐efficacy of condom use negotiation and condom‐less anal sex (CAS). During the study, one HIV‐positive peer counsellor administered six 2‐hour counselling sessions to 11 HIV‐positive and 10 HIV‐negative gay Latino men living in Toronto, Canada. A T‐paired test and a McNemar test assessed GPS’ effects 12 months after the intervention began.

**Results**

Results show a reduction in CAS (any partner) from 90% of the sample at baseline to 62% at the 12‐month follow‐up. The findings also indicate a significant increase in the self‐efficacy of negotiating condom use (in both HIV‐positive and HIV‐negative men) and a decrease in loneliness in HIV‐negative men.

**Conclusions**

The findings provide preliminary evidence that GPS, a counselling intervention led by peers, may offer an efficient way of concurrently reducing CAS, mitigating mental health problems and increasing condom use negotiation in Latino gay men.

## P038

### ISTeja Prevenido: prevention actions developed by college student

**A Martinez^1^, J Horvath^2^, A Cruz^1^, C Martins^1^, G Mattei^1^, V Vedana^1^, Y Lombardo^1^, F Mucelini^1^, Y Borges^3^, M Alves^1^, R Cavalheiro^1^, K Minosso^1^, J Conterno^1^ and B Amaral^1^**

^1^Center of Biological Sciences and Health, State University of Western Paraná, Cascavel, Brazil. ^2^Municipal STD/AIDS and Viral Hepatitis Program, City Hall of Cascavel, Cascavel, Brazil. ^3^Center of Applied Social Sciences, State University of Western Paraná, Cascavel, Brazil

**Background**

In Brazil, we noted that HIV and syphilis in young people is increasing, then although the prevention strategies adopted in our country, we still observe that in this kind of population exist a low‐risk perception and testing, that are facts that induces a late access to treatment [1]. According to the period 2006 to 2015 epidemiologic data showed triple the numbers of cases in 15 to 19 years old age group and double in 20 to 24 years old age group [2]. Aiming contribute to sexually transmitted diseases (STD) prevention between college students, an extension project named “ISTeja Prevenido” begun at the State University of Western Paraná – Unioeste, Cascavel Campus.

**Materials and methods**

The college students who participated in the State Meeting of Youth Leadership in STD Prevention, AIDS and Viral Hepatitis, promoted by the Secretary of State for Health, begun the project by holding weekly meetings to discuss strategies to improve the STD prevention within the student college at Unioeste. Those activities include: creation of communicating ways, like social medias to share information with academic ways, condom distribution, educate activities and testing campaign, than this last one, the college students of the project were training by the Specialized Center for Parasitic Infectious Diseases (CEDIP) of Municipal Secretary of Health in Cascavel/PR.

**Results**

The project created communicating ways elaborating a Facebook page (https://www.facebook.com/coletivoistejaprevenido/) and an Instagram account (@istejaprevenido) to spread educate content and divulgate all project actions. Condoms distribution is a consoled strategy in health services, hence little disseminated out that ambiences. However, a strategy adopted in this project was distributed during congresses and letters, and a continuing way was leave custom boxes containing condoms, in bathrooms in university. To discuss this theme, were performed two educative activities, one of them was the Talk Show and another one was a conversation wheel, that made it possible to clarify doubts and discuss themes, like STD transmission, humanisation in health services and preconceptions related HIV. Lastly, two testing campaigns were done inside the university that made it possible to identify HIV and syphilis cases.

**Conclusions**

In 2018, 4946 condoms were distributed, 196 college students participated in our educational actions and 261 quick tests were performed. The development of this project evidences that the participation of own young people in realisation of those kind of prevention actions, made the dialogue more easily and encouraged the comportment change.

**References**

1. Guimarães Mark Drew Crosland, Carneiro Mariângela, Abreu Daisy Maria Xavier de, França Elisabeth Barboza. HIV/AIDS Mortality in Brazil, 2000‐2015: Are there reasons for concern?. Rev bras epidemiol [Internet]. 2017 [cited 2019 Jan 17]; 20 Suppl 1: 182–190. Available from: http://www.scielo.br/scielo.php?script=sci_arttext{00AMP00}pid=S1415‐790X2017000500182{00AMP00}lng=en. https://doi.org/10.1590/1980‐5497201700050015.

2. Brasil. Ministério da Saúde (BR). Secretaria de Vigilância em Saúde. Departamento de DST, Aids e Hepatites Virais. HIV/AIDS. Bol Epidemiológico [Internet]. 2016 [cited 2018 Jul 18];5(1):1–58. Disponível em: http://www.aids.gov.br/pt‐br/pub/2016/boletim‐epidemiologico‐de‐aids‐2016 [Links].

## Models of Care/Scale Up of Treatment

## P039

### Trends in CD4 cell count at diagnosis of HIV and initiation of ART in Haiti (2004 to 2016)

**A Dua^1^, S Bousleiman^1^, A Marcelin^2^, V Rivera^2,3^, S Koenig^4^, P Cremieux^1^, M Deschamps^2^ and J Pape^2,3^**

^1^Analysis Group, Inc., Boston, MA, USA. ^2^The Haitian Group for the Study of Kaposi's Sarcoma and Opportunistic Infections (GHESKIO), Port‐Au‐Prince, Haiti. ^3^Weill Cornell Medicine, New York, NY, USA. ^4^Brigham and Women's Hospital, Boston, MA, USA

**Background**

World Health Organization (WHO) guidelines have progressively changed over the past 15 years, to recommend earlier initiation of antiretroviral therapy (ART) for people living with HIV (PLWH). In 2006, 2009 and 2013, ART was recommended for PLWH with CD4 count <200 cells/mm^3^, ≤350 cells/mm^3^ and ≤500 cells/mm^3^ respectively. In 2015, universal ART was recommended. However, despite these changes in guidelines, a substantial proportion of patients continue to present with advanced disease. We analysed temporal trends and predictors of late presentation and late initiation of ART at GHESKIO (Haitian Study Group for Kaposi's Sarcoma and Opportunistic Infections) in Port‐au‐Prince, Haiti.

**Materials and methods**

This retrospective cohort study included electronic medical record data on patients >18 years who were diagnosed with HIV at GHESKIO between 2004 and 2016. We analysed trends in CD4 count at HIV diagnosis and ART initiation over time. We examined demographic predictors associated with CD4 count <200 cell/mm^3^ in 2016 with multivariable logistic regression.

**Results**

In total, 32,751 patients tested positive for GHESKIO from 1 January 2004 to 31 December 2016 and were included in the analysis. Of these, 19,320 (59%) patients had blood drawn for CD4 count within six months of HIV diagnosis or ART initiation. The median CD4 count at HIV diagnosis increased over time, from 172 cell/mm^3^ (interquartile range (IQR): 44, 387) in 2004 to 347 cell/mm^3^ (IQR: 176, 551) in 2016 (*p* < 0.01) (Figure 1). Similarly, the median CD4 count at ART initiation increased from 2004 to 2016, with the lowest median CD4 count of 63 cell/mm^3^ (IQR: 21, 152) observed in 2005, and the highest median CD4 count of 402 cell/mm^3^ (IQR: 212, 634) observed in 2016 (*p* < 0.001) (Figure 1). The proportion of patients initiating ART with CD4 count <200 cell/mm^3^ decreased from 80.9% in 2004 to 24.1% in 2016. In the latest study year (2016), age (decade) was the only predictor of  CD4 count <200 cell/mm^3^ at HIV diagnosis (odds ratio (OR) 1.26; 95% confidence interval (CI): 1.06 to 1.51). Age and education status were also significantly associated with initiating ART with CD4 count <200 cell/mm^3^.

**Conclusion**

Over the past 15 years, the proportion of patients presenting with advanced HIV disease has substantially declined in Haiti, and CD4 counts at ART initiation have significantly increased. Age and education status are associated with late treatment initiation, highlighting the need for additional efforts for early testing and linkage to care in this population.

Abstract P039‐Figure 1. Trends in median CD4 cell count at HIV diagnosis and ART initiation over time. Dashed lines indicated change in WHO guidelines for ART initiation.

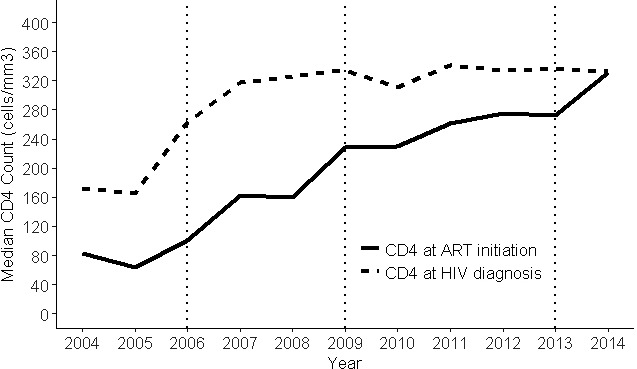

## P040

### An individualised communication strategy to enhance retention and ART uptake in naïve individuals: a randomised controlled trial

**M Bullo^1^, M Michaan^2^, M Fuentes^1^, V Losso^1^, L Pastore^1^ and M Losso^1^**

^1^HIV Unit, Hospital Ramos Mejia, CABA, Argentina. ^2^Infectologia, Hospital San Juan de Dios, La Plata, Argentina

**Background**

Several strategies have been designed to improve retention in care and viral suppression (VS) in patients starting antiretroviral therapy (ART). In Latin America, few strategies have been locally tested in a randomised way and evidence‐based decisions are scarce.

**Materials and methods**

From 2015 to 2018, we conducted a multicentric RCT including naïve patients prescribed ART in two public hospitals in Argentina. The main goal was to compare retention in care between arms. Subjects starting ART were randomly assigned to standard of care follow‐up (SOC) or SOC plus an individualised communication strategy (ICS). Every four weeks up to one year, trained personnel contacted patients using patient‐selected communication method to screen for retention and adherence problems with a semi‐structured interview. The primary outcome was successful linkage, defined as ambulatory care in the last six months with no ART interruption. Descriptive statistics were used for baseline characteristics. Risk differences (RD) and 95% confidence intervals (CI) were estimated using linear regression for primary and secondary outcomes obtaining crude and adjusted estimates. Secondarily, we wanted to assess, in the intervention arm, if the number of successfully established contacts (NEC) was associated with successful linkage during the first year after randomisation. Poor NEC (PNEC) was defined as ≤4 contacts, and fair NEC (FNEC) as ≥4.

**Results**

A total of 207 participants were randomised (107 to SOC, 100 to ICS). The median age was 31 years (IQR 26, 40), 81% were male, 62% were MSM, 26% were immigrants. The median baseline HIV‐RNA log was 4.44 (IQR 3.73, 5.10) and CD4 count/mm^3^ was 398 (IQR 220, 584). There was not significant RD in successful linkage across arms in the crude (SOC = 0.61, ICS = 0.67, RD = 0.06, 95% CI: −0.07, 0.19) and adjusted by sex, age, transmission category, immigration status and baseline CD4 (SOC = 0.28, ICS = 0.36, RD = 0.08, 95% CI: −0.06, 0.21) estimates. Four hundred and seventy‐seven contacts were established. Mean number of contacts was 4.91. Sixty‐four participants had FNEC and thirty‐six, PNEC. FNEC and PNEC subgroups had no significative difference across covariates. Successful linkage was 0.4 higher among FNEC than among PNEC (95% CI: 0.21, 0.58; FNEC: 0.81; PNEC: 0.42). In the migration‐status adjusted model, RD was 0.42 (95% CI: 0.24, 0.60; FNEC: 0.61; PNEC: 0.19) (Table 1).

**Conclusion**

In a mostly male MSM cohort in Argentina, linkage was successful one year after ART initiation in approximately two thirds of the population. However, over one quarter was not linked to care at one year. The communication strategy showed increased linkage success, but not statistically significant. New strategies need to be developed to engage hard‐to‐retain patients.

**Abstract P040‐Table 1. Summary table of baseline characteristics overall and across arms of the RCT**

**Table:** 

	Total (n = 207)	Arm A (n = 107)	Arm B (n = 100)
Demographic characteristics			
Age (median, IQR)	31 (26, 40)	31 (26, 40)	31 (26, 40)
Male at birth	0.81	0.79	0.83
MSM	0.62	0.61	0.64
Education years (median, IQR)	12 (10, 14)	12 (10, 14)	12 (9, 13)
Non‐immigrant	0.74	0.76	0.71
Comorbidities			
Recreational drug user	0.25	0.24	0.27
Alcohol user	0.52	0.5	0.55
History of psychiatric treatment	0.03	0.05	0.01
Clinical and laboratory data			
Baseline events	0.03	0.04	0.02
Baseline viral load (log)	4.44 (3.73, 5.10)	4.60 (3.86, 5.17)	4.37 (3.63, 5.07)
Baseline CD4 count (median, IQR)	398 (220, 584)	389 (195, 561)	415 (266, 604)

## P041

Abstract withdrawn

## P042

## Monitoring treatment gap of HIV in Brazil: a national public system to surveillance healthcare for PLHIV

**R Vianna Brizolara, P Adamy, A Kolling, A Sposito Tresse, F de Barros Perini and G Fernando Mendes Pereira**

Department of STI, HIV/AIDS and Viral Hepatitis, Ministry of Health, Brasília, Brazil

**Background**

Brazil has been implementing the Clinical Monitoring System of People Living with HIV (SIMC) since 2013, the same year the country adopted test and treat strategy as a free of charge public policy. Nowadays, SIMC monitors people living with HIV (PLHIV): 1) without antiretroviral therapy (ART), 2) lost to follow‐up (>100 days without antiretroviral delivery) and 3) who have detectable viral load despite ART. It remains an important tool to manage the process of continuous care of PLHIV and develop national efforts to scale up early treatment of HIV patients in partnership with municipalities and states.

**Materials and methods**

A cross‐sectional study was carried out to observe the tendency of the number of PLHIV without ART (treatment gap). The study population was PLHIV with viral load results (>50 copies/mL), and without antiretroviral delivery. The database was the SIMC; it realises data crossing from National Laboratory Test Control System (SISCEL) and National Antiretroviral Delivery Control System (SICLOM).

**Results**

Between January 2014 and January 2019, 193,654 PLHIV who had not started ART were identified by SIMC. Most of them, 159,191 cases, were analysed, 141,961 patients were located and started treatment over the period, and 43,043 were not found out yet by health teams and had not started ART. Between July 2016 and January 2019, 27,364 PLHIV left treatment gap and started ART (Figure 1).

**Conclusions**

Despite Brazil's intensified HIV testing and treatment efforts, PLHIV remain diagnosed without treatment. Maintaining HIV surveillance is recommended to strengthen tools such as SIMC to improve healthcare for PLHIV, creating conditions to move toward 90‐90‐90 targets for HIV and eliminate AIDS epidemic until 2030.

Abstract P042‐Figure 1. PLHIV in treatment gap, Brazil, July 2016 to January 2019.

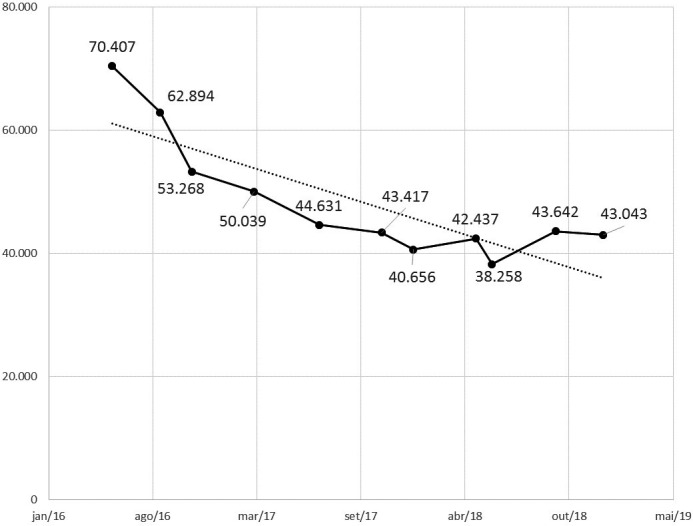

## P043

## Why do HIV‐infected patients miss scheduled appointments?

**M Sandoval, M Kundro and M Losso**

HIV Unit, Hospital Gral. de Agudos J. M. Ramos Mejía, CABA, Argentina

**Background**

Effective management of HIV as a chronic disease needs a continuum of care which can only be achieved if patients are retained in care. Missed clinic visits represent a significant problem as they have been related to worse clinical outcomes, such as virologic failure and mortality. Moreover, missed appointment rates are a significant predictor of loss to follow‐up. Understanding the reasons for missed appointments may help to increase adherence to clinical schedules. We examined the reasons why HIV‐infected patients missed HIV medical visits in a large public hospital in Buenos Aires.

**Materials and methods**

We performed a cross‐sectional analysis including all subjects who missed an HIV‐care appointment during a six‐month period. Missed appointments were defined as those visits that were not cancelled either by the patient or by the clinic for which the patient did not arrive. We collected patient‐level data on demographic characteristics, immunological and virological status, and evaluated the reasons of missed visits through telephone calls or self‐administered surveys.

**Results**

During the study period, 631 patients who scheduled an appointment missed one or more clinic visits, contributing to a total of 830 missed appointments. Overall, the median age was 40 years (IQR 39 to 41), 67.4% were male, 73.1% were Argentinean, 87.5% did not have medical insurance and 60% did not have a formal employment. Of these subjects, 67.7% (244 out of 360) patients have an undetectable viral load, and as much as 42.9% patients did not perform any viral load or CD4 count during the previous year. Remarkably, 13.4% (91 out of 675) interrupted antiretroviral therapy for at least one month in the last year. A total of 575 (69.2%) self‐reported reasons for missing the clinic appointment were obtained. The most frequently cited were: “forgetting or confusing the time of the appointment” (18.6%), “overlapping with work schedules” (18.2%) and “health problems” (9.7%). Fourteen per cent of patients answered that they did not remember why they missed the appointment.

**Conclusions**

We identified some frequent factors that caused patients to miss appointments. Patients who underused HIV care had a negative impact on clinical outcomes in this cohort. Novel interventions aimed at reducing missed appointments need to be based upon these findings.

## P044

### Modest progress in early linkage to care in Latin American HIV‐care centres from the HIV Latin American Workshop Group (2013 to 2017)

**P Belaunzarán‐Zamudio^1^, P Zitko^2^, A Araúz Rodríguez^3^, A Celi^4^, J Chaverri^5^, C Gallo^6^, E González^3^, M Greco^7^, M Hojman^8^, D Larreategui^9^, M Lasso^10^, J Maquera Afaray^11^, G Loza^12^, A Lucchetti^13^, M Morales^14^, S Valderrama^15^, C Beltrán^16^ and Taller Latinoamericano de VIH^17^**

^1^Departamento de Infectología, Instituto Nacional de Ciencias Médicas y Nutrición Salvador Zubirán, Mexico City, Mexico. ^2^Departamento de Enfermedades Infeciosas, Complejo Asistencial Barros Luco, Santiago, Chile. ^3^Infectología, Hospital Santo Tomás, Panama, Panama. ^4^Departamento de Infectología, Hospital de las Fuerzas Armadas, Quito, Ecuador. ^5^Departamento Clínico, Hospital Dr. Rafael Ángel Calderón Guardia, San José, Costa Rica. ^6^Departamento de Epidemiología, Hospital Regional de Arica, Arica, Chile. ^7^Infectología, Centro de Estudio y Tratamiento Infectológico, La Plata, Argentina. ^8^Departamento de Infectología, Hospital General de Agudos, Buenos Aires, Argentina. ^9^Clinica Pichincha, Hospital Carlos Andrade Marín, Quito, Ecuador. ^10^Departamento de Infectología, Hospital Sótero del Río, Santiago, Chile. ^11^Departamento de Infectología, Hospital Nacional Guillermo Almenara, Lima, Peru. ^12^Infectología, Hospital Eugenio Espejo, Quito, Ecuador. ^13^Infectología, Hospital Nacional Arzobispo Loayza, Lima, Peru. ^14^Infectología, Hospital Militar Dr Carlos Arvelo, Caracas, Venezuela. ^15^Infectología, Hospital Universitario San Ignacio, Bogotá, Colombia. ^16^Infectología, Complejo Asistencial Barros Luco, Santiago, Chile. ^17^Taller Latinoamericano de VIH, Santiago, Chile

**Background**

Delays in HIV diagnosis and enrolment in care have been persistent barriers to achieve the 90‐90‐90 WHO goals to end the HIV/AIDS epidemic as a public health problem by 2030 in Latin America. Between 38% and 45% of patients in our region enrol in care with advanced HIV disease (CD4 counts <200 cells/μL) driving steadily high HIV‐mortality rates in Latin America. Moreover, more than 60% present late to care (CD4 < 350 cells/μL). Our goal is to describe the presentation to care among centres from the HIV Latin American Workshop from 2013 to 2017.

**Material and methods**

We performed an ecological analysis using collected data from 31 centres in 10 Latin American countries. Data were organised in 12 strata of age and sex, 4 strata of CD4 cells (<200, 200 to 349, 350 to 499, >499 cells/μL) and 5 strata of calendar‐year of enrolment (2013 to 2017). We defined “timely presentation” (TP) as CD4 > 499 cells/μL, late presentation (LP) as CD4 count <350 cells/μL and advanced HIV disease (AIDS) as <200 cells/μL, all at enrolment. We used contingency tables and random effect (strata, centre and country) logistic models to observe trends over time of these conditions. We explored covariables associated to AIDS at enrolment and reported crude and adjusted odd ratios (OR). In sensitivity analysis, we considered weights based on the expected fraction of people analysed in respect to the total population receiving care by country using distributional assumptions.

**Results**

We analysed data of 34,679 (7.4%) people of an estimate of 470,165. During 2013 to 2017, LP decreased from 60.7% to 52% and AIDS at enrolment from 37.1% to 28.4%, with important heterogeneity by country and age group (Table 1 and Figure 1). TP increased from 21.4% to 27.7% (*p* < 0.05). In multivariate analysis, covariables associated to decreased risk of AIDS at enrolment were: younger age, female gender (OR 0.91, 95% CI: 0.85 to 0.97), enrolment in centres with private funding (OR 0.54, 95% CI: 0.32 to 0.92) and being enrolled in care in recent years (2016 to 2017). Heterogeneity for AIDS between countries was around 1%, while between centres close to 6%.

**Conclusions**

We reported a large observational study showing that AIDS and LP at enrolment in care decreased in recent years in Latin America, but heterogeneity in trends across countries is large with important differences by age group and source of funding. Nevertheless, LP is still an important problem in most countries of our region with consequences in HIV transmission, morbidity and mortality.

**Abstract P044‐Table 1. Covariates associated to presentation to care at advanced HIV infection (LP) (<200 cells/μL)**

Abstract P044‐Figure 1. Trends in the frequency of (A) HIV‐infection stage according to CD4 count at enrolment in 31 HIV‐care centres in 10 countries in Latin America and (B) in the frequency of AIDS at enrolment (<200 cells/μL) by country (2013 to 2017). Percentages estimated over the total number of patients enrolled each year overall (A) and in each centre (B).

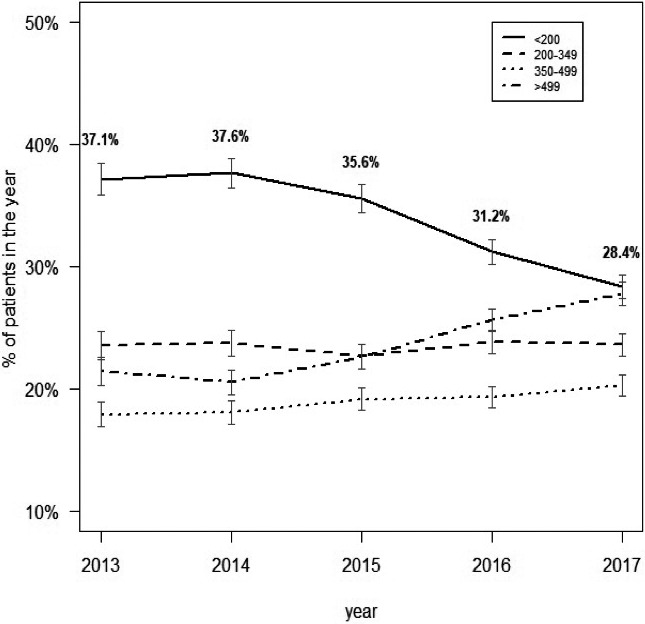

**Table:** 

Covariable	Univariate	Multivariate
	OR (95% CI)	OR (95% CI)
Female versus male	1.02 (0.96, 1.09)	0.91 (0.85, 0.97)
Age group (in years)		
15 to 29 (reference)	1
30 to 39	2.03 (1.92, 2.15)	2.02 (1.90, 2.13)
40 to 49	2.80 (2.62, 3.00)	2.77 (2.59, 2.97)
50 to 59	3.07 (2.82, 3.35)	3.04 (2.79, 3.32)
60 to 69	3.32 (2.86, 3.85)	3.26 (2.81, 3.78)
≥70	3.95 (2.97, 5.24)	3.93 (2.96, 5.21)
Year of enrolment		
2013 (reference)	1	1
2014	1.03 (0.96, 1.12)	1.06 (0.98, 1.15)
2015	0.98 (0.90, 1.06)	1.01 (0.94, 1.10)
2016	0.81 (0.75, 0.88)	0.87 (0.80, 0.94)
2017	0.71 (0.66, 0.76)	0.78 (0.72, 0.84)
Private versus public centre	0.69 (0.45, 1.07)	0.50 (0.30, 0.82)
Centre in capital versus province	0.85 (0.58, 1.23)	0.94 (0.64, 1.36)
Specialised MD	0.94 (0.80, 1.11)	0.85 (0.71, 1.01)
Shortage VL	1.18 (0.68, 2.05)	0.73 (0.42, 1.28)
Shortage ART	1.05 (0.65, 1.71)	1.34 (0.81, 2.23)

## P045

### HIV rapid test plus linkage to care programme in Argentina: experience from a NGO

**N Haag^1^, M Laurido^2^, M Pedrola^1^**

^1^Testing and Prevention, AIDS Healthcare Foundation Argentina, Buenos Aires, Argentina. ^2^Medical Quality, AIDS Healthcare Foundation Argentina, Buenos Aires, Argentina. ^3^Global Public Health Institute, AIDS Healthcare Foundation, Buenos Aires, Argentina

**Background**

According to the Argentina's Ministry of Health report of 2018, HIV prevalence in adults is 0.4%, 80% out of them are diagnosed and 83.5% of them are on antiretroviral treatment. Aids Healthcare Foundation (AHF) is committed with the 90‐90‐90 proposal by UNAIDS and carrying on since 2013 annual prevalence assessments based on rapid testing programmes and linking to care for those with positive results. Therefore, the complete information available for 2018 is presented.

**Methods**

Between 2 January 2018 and 30 December 2018, HIV antibody determinations were performed in individuals from different provinces of Argentina, which together represent more than 60% of the total country population, through a previously validated rapid test (Alere Determine™ HIV‐1/2). Counselling was given to all people. For positive cases, the referral for confirmation and linkage to care was made through a programme using case managers in charge of accompanying patients until the third visit in their health system. The information was collected in an *ad hoc* database. Chi‐square, Fisher exact or Mid P tests and maximum likelihood odds ratios were used as appropriate.

**Results**

Determinations were made in 54625 subjects. Age range: 18 months to over 49 years. Gender distribution: women 61.4%, men 38%, transgender 0.6%. It was the first HIV test for 58.4%. There were 477 HIV‐positive results. Prevalence: 0.87% (95% CI: 0.80 to 0.95). Prevalence was significantly higher in transgender and in men than in women (1.68%, 1.62%, 0.40%, respectively; *p* < 0.001). There was a greater probability of having a positive test in men who had sex with men (OR: 6.11 – 95% CI: 4.57 to 8.16; *p* < 0.001) and people between 25 and 49 years old (OR: 1.63 – 95% CI: 1.35 to 1.97; *p* < 0.001). In terms of linkage to care, 263 subjects were linked to an AHF‐supported facility and 161 were linked to a non‐AHF facility. In total, 88.9% of subjects with positive results were linked to care.

**Conclusions**

HIV prevalence observed in this population was twice as high as that reported by the Ministry of Health for the adult general population of Argentina. Linkage to care mediated by AHF was high, but continuous efforts should be maintained to reach the percentages recommended by UNAIDS.

## P046

### Regulation and health market effects in the delivery of HIV health services in Colombia

**L Arevalo, L Garavito, C Alvarez and A Perez**

Investigación, Processum Consultoria Institucional, Bogotá, Colombia

**Background**

The Colombian health system relies on a regulated market for the provision of health services. Both public and private hospitals compete for capitated contracts. A comprehensive health services package is usually settled on a yearly timeframe basis. The Ministry of Health defines a basket of services with the benefits and their expected use frequencies for each period. The aims for this study is to characterise contracting models, the fulfilment of regulatory guidelines, the incentives applied and their effect on health outcomes.

**Materials and methods**

An electronically delivered survey for health insurers covering 57% of the affiliated population (roughly 25 million Colombians) was issued. The sample includes insurers responsible for 49,000 persons living with HIV (PLHIV). This represents 66% of the total PLHIV reported in the cohort databases for their clinical surveillance. A descriptive and correlation analysis was performed following the study objectives.

**Results**

Capitation is the single payment method used for all insurers to purchase the preventive component of the benefit package. Incentives and indicators for the screening of pregnant women were identified. However, no indicators and incentives for other populations at risk (drug users, migrants, mine workers, violence displaced people) were individualised. 58% of the insurers do not provide community services. Their main argument was poor supply of these services in some Colombian regions. 98% of the outpatient services are bought from hospitals as comprehensive delivery packages. These services are provided in large Colombian cities, 22% of PLHIV must travel to other cities to get medical services. Relevant variation in the availability of specialised physicians was documented. Psychiatrists and professional pharmacists show the largest human resources shortages. Just 17% of the health insurers apply incentives directed towards improved health outcomes.

**Conclusions**

The study results indicate deficiencies in the health service networks affecting PLHIV. Centralised contracting services generates an inequity burden for those living in the poorer and underserved urban and rural areas of the country as health services delivery to these sparsely populated areas is not provided. Contracting practices have focused on the compliance of regulated services and expected frequencies, avoiding health outcomes. The lack of evidence for the application of risk adjusted reimbursement schemes indicates low emphasis in risk management and intervention from the insurers. The Colombian Ministry of Health should review the health insurance regulatory framework inducing changes in health service delivery models, incentives alienation and the innovation in contracting practices.

## P047

### Effectiveness in follow‐up and recapture interventions in patients who leave an HIV programme care in Bogotá, Colombia between 2015 and 2017

**L Arevalo, C Oviedo, N Villabona, R Pulido, M Macias, C Avila and M Mantilla**

Centro de Expertos para Atencion Integral, Bogotá, Colombia

**Background**

A fundamental factor in the HIV management has been to achieve the adherence to antiretroviral treatment, whose failure brings consequences produced by the increase in morbidity and mortality. The aim of this study is to determine the prevalence of non‐adherence, and to evaluate the effectiveness of the follow‐up area of an HIV care centre; this area is responsible for recovering non‐attendant patients (those without physician consultations in a period of more than six months and/or who do not make the claim of medicines in the following month in the Centre).

**Materials and methods**

A cross‐sectional retrospective study was developed in the CEPAIN HIV programme care at Bogotá, Colombia. The databases of patients not attending to the HIV programme in the period 2015 to 2017 were reviewed, with subsequent review of clinical histories.

**Results**

During the period from 2015 to 2017, the programme had 4551 patients, follow‐ups were performed in 555 patients who were identified as patients who left the programme or were inactive due to absences of more than six months. 2015 started with 134 patients, 2016 detected 214 and 2017 141 patients (Figure 1), this implies a lost‐to‐care of antiretroviral treatment in 3.17%, 4.68% and 4.55% for each year. It was identified that the discontinuation rate is higher in women than in men, since for every 100 women registered 5.3 do not return on average in the three years, while for every 100 men, 4.4 leave the programme (Figure 1). The Engaged‐in‐Care with the intervention of the follow‐up group was 88.8%, 64.0% and 63.8% for those years, the average time between diagnosis and the abandonment of patients who do not return is 5.4 years 95% CI (4.7 to 6.0).

**Conclusion**

The factors associated with non‐adherence have been widely described, but the effectiveness of the intervention is associated with having a work team aimed at identifying factors that may lead to abandonment and establishing concerted, educational and individual actions with the patient that facilitate their re‐entry and guarantee the continuity of the services and ensure that the patient understands the risk of non‐adherence.

Abstract P047‐Figure 1. Dropout rate by gender.

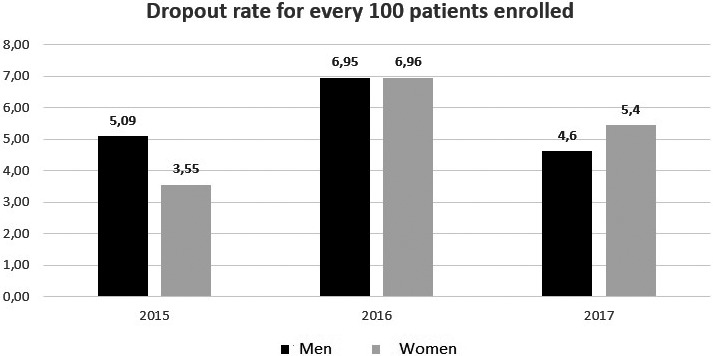

## P048

### Impact of supervised antiretroviral therapy in patients with HIV who have no adherence to therapy and are with detectable viral load

**L Coral^1^, H Paez Ardila^2^, C Hernandez^3^, N Fonseca^4^, F Barrientos^3^, K Romero^3^, E Mujica^4^ and O Sussman^2^**

^1^Chemical Pharmacist, Asistencia Cientiica de Alta Complejidad, Bogotá, Colombia. ^2^Infectious Diseases, Asistencia Cientiica de Alta Complejidad, Bogotá, Colombia. ^3^Physician, Asistencia Cientiica de Alta Complejidad, Bogotá, Colombia. ^4^Nursing Department, Asistencia Cientiica de Alta Complejidad, Bogotá, Colombia

**Background**

Therapeutic adherence is a key factor to ensure the sustainability of the health system and ensure adequate virological suppression in patients with HIV; however, there are patients who do not have adequate adherence to ART, which leads to a risk of opportunistic infections and antiretroviral drug resistance. For patients who have tuberculosis, a strategy of daily medication dispensation is designed, which leads to better clinical outcomes. Our centre sees approximately 8000 patients with HIV in Colombia each month, and of these the great majority is in the capital city Bogotá. We have patients who, despite being educated by the support group (psychologist, social worker, pharmacist, doctor and nurse), cannot improve their adherence. Therefore, an attempt was made to extrapolate the tuberculosis strategy and we discharged the antiretroviral drug for non‐adherent patients, daily, weekly or twice a month and then we evaluated the results of the intervention.

**Materials and methods**

A descriptive study of supervised therapy and its impact on patients without adherence to ART who present with virological failure, in the period from January to November 2018 in a HIV care centre was performed. Forty‐seven patients were included who entered in the supervised therapy programme which was dispensed by the pharmacy service daily, weekly or twice a month, the storage of the ART was done in individual boxes labelled for each patient. With face‐to‐face and telephone follow‐ups. Viral load monitoring was carried out at two months and finally six months after the intervention.

**Results**

Of the 47 patients who underwent supervised therapy, 20 patients remained non‐adherent despite the supervised therapy, 6 patients despite being fully adherent had virological failure defined as HIV viral load >200 copies/mL after six months of adjustment of adherence, and 21 patients managed to have control of the viral load (<200 copies/mL) after six months considering how therapeutic success (Figure 1).

**Conclusions**

The supervised therapy in non‐adherent HIV patients, carries to 45% of patients who achieve virological suppression below 200 copies/mL. We consider it to be a highly recommended strategy and intervention to avoid resistance to antiretroviral drugs and, consequently, a lower probability of hospitalisations and opportunistic infections.

Abstract P048‐Figure 1. Distribution of patients with VL < 200 copies/mL, after intervention.

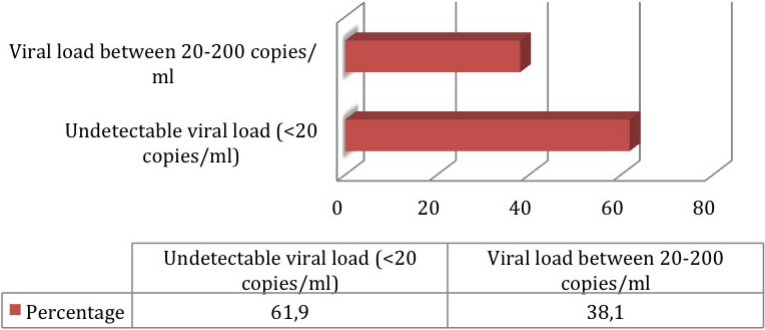

## P049

Abstract withdrawn

## P050

### A comparison of baseline conditions and time to initiation of ART between 2005, 2011 and 2016 at an HIV treatment centre in Lima, Peru

**J Hidalgo and R Ayarza**

^1^HIV Clinic, Via Libre, Lima, Peru. ^2^HIV Research, Via Libre, Lima, Peru

**Background**

Timely initiation of antiretroviral therapy (ART) is a global priority to improve survival of HIV‐infected persons and to decrease transmission of HIV. Late diagnosis and limited access to prompt initiation of treatment still affect the capacity of many countries to overcome the consequences of the AIDS epidemics. We aimed to evaluate progress over time in this regard at a Clinic affiliated with Peruvian national HIV treatment programme.

**Materials and methods**

We reviewed medical records of patients that initiated ART for the first time at Vía Libre, a large HIV Clinic in Lima, Peru, in 2005, 2011 and 2016. Baseline conditions, time to access to therapy and early complications were evaluated to compare between the three selected years.

**Result**

For each year, similar numbers of patients were identified: 98 for 2005, 92 for 2011 and 114 for 2016 (a sample for this year, total was 357). Table 1 shows the results. For the three years reported, the median age was similar, and gender was predominantly masculine. Baseline CD4+ cell count showed a gradual increase and there was a decreasing proportion of patients presenting with clinical stage IV at initiation. Access to ART improved: time between HIV diagnosis and initiation of ART, and time between initiation of care at Vía Libre and initiation of ART, showed a decreasing trend for each evaluated year. Significant early (<3 months) ART toxicity, leading to a modification or interruption of HAART was higher in 2005 and 2011, in comparison to 2016. Treatment regimens were always predominantly NNRTI based (98.9% to 99.1%). In 2016, use of zidovudine was clearly diminished. Complications associated to progression of disease or immune reconstitution inflammatory syndrome (IRIS), observed within three months of initiation of ART, were higher in 2005 and less frequent in following years. This included early mortality cases (n = 5), which were observed only in 2005.

**Abstract P050‐Table 1. Comparison of main results for the three years selected**

**Table:** 

Year (number of individuals)	2005 (n = 97)	2011 (n = 98)	2016 (n = 114)
Median age (years)	4.7	34.2	33.2
Male (%)	77.6	87.0	89.2
Mean baseline CD4+ count (cells/mm^3^)	108.2	247.6	318.3
Clinical stage IV (%)	96.9	48.4	25.4
Time diagnosis of HIV to initiation of ART (years)	2.45	1.43	0.82
Time initiation of care to initiation of ART (years)	0.8	0.63	0.44
Initial use of zidovudine (%)	76.5	84.8	3.5
Significant early (<3 months) ART toxicity (%)	34.7	35.8	14.9
Progression of disease + IRIS events (<3 months) (%)	71.4	48.9	25.4

**Conclusions**

Since launch of the HIV treatment programme by the Peruvian Ministry of Health in 2004, we have observed ART initiation over the following years with higher CD4 count, a higher proportion of clinically stable patients, shorter time to initiation since diagnosis, and a lower proportion of early complications. This progress reflects changes in criteria to start of treatment and improvements in access, but still needs to get much closer to current international goals.

## P051

### Late HIV presentation in a Venezuelan hospital

**M Morales, M Niño, F Moy, V Ochoa, M Benitez, Y Gil and O Perez**

Infectious Diseases, Hospital Militar Dr Carlos Arvelo, Caracas, Venezuela

**Background and objective**

In order to control the HIV epidemic, it is essential to intensify early diagnosis, as a strategy to reduce late presentation to 10% [1]. Previous reports in Latin America found percentages between 38% and 45% of late presentation with advanced disease (CD4 T lymphocytes <200 cells/μL), and an additional 23% with late presentation (CD4 T lymphocytes: 200 to 350 cells/μL) [2]. To determine the frequency and associated factors of late presentation (LP) and with advanced disease of HIV infection (LPAD).

**Materials and methods**

Cross‐sectional study with secondary data of adult patients diagnosed with HIV during the 2014 to 2016 period.

**Results**

From 160 patients, 104 (65%) had LP, 71 (44.4%) LPAD. The following results were obtained: mean count of CD4 320 cells/μL and 54% with <15%; average viral load 134,410 copies/mL and 81% reported >10,000; 49% with age <28 years; 78.8% men with LP, 55% heterosexual, 30% MSM of which 71% versatile role; Singles 85.6%, couple fixes 45%, couple HIV positive 23%, intravenous drug users 6.7%, sex with a sex worker 9.6%, previous transfusions 6.7%, LP asymptomatic 17.3%; associated wasting 42%, >2 opportunistic infections 12%, more frequent confection: tuberculosis; one case with cervical cancer and one case with Kaposi's sarcoma. 37% performed rapid HIV testing and 91.9% attended 1 consultation in the first month of diagnosis. 36.5% required hospitalisation in the first month. Frequent paraclinical findings: anaemia, lymphopenia and thrombocytopenia.

**Conclusions**

Understanding local data and associated factors helps develop public surveillance programmes by providing targeted intervention, focusing on people at high risk [3]. HIV testing should be intensified, stigma should be reduced, the benefits of treatment should be made known and retention should be improved in consultation.

**References**

1. Raffetti E, Postorino MC, Castelli F, et al. The risk of late or advanced presentation of HIV infected patients is still high, associated factors evolve but impact on overall mortality is vanishing over calendar years: results from the Italian MASTER Cohort. BMC Public Health. 2016;16(1):878.

2. Waters L, Sabin C. Late HIV presentation: epidemiology, clinical implications & management. Expert Rev. Anti Infect Ther. 2011;9(10):877–89.

3. Celi AP, Greco M, Martinez E, Vargas C, Belaunzaran F, Mejia F, on behalf of The Latinamerican Workshop Study Group. Presentation to care with advanced HIV disease is still a problem in Latin America. J Int AIDS Soc. 2016; 19 2Suppl 1: 21083.

## Opportunistic Infections

## P052

Abstract withdrawn

## P053

### Disseminated histoplasmosis in a population with AIDS in Pereira, Colombia

**J Hoyos Pulgarín^1^, J Sierra Palacio^2^, A Jaramillo Torres^2^, J Alzate Piedrahita^3^ and K Ordoñez^4^**

^1^Infectious Disease and Internal Medicine, Universidad Tecnológica de Pereira, Pereira, Colombia. ^2^Medicine, Universidad Tecnológica de Pereira, Pereira, Colombia. ^3^Internal Medicine, Universidad Tecnológica de Pereira, Pereira, Colombia. ^4^Infectious Disease and Internal Medicine, Hospital Universitario San Jorge, Pereira, Colombia

**Background**

Histoplasmosis is an endemic mycosis considered worldwide as an orphan disease [1], which has a very variable clinical presentation and usually is disseminated in the population with HIV/AIDS [2]. Knowing that clinical suspicion and early treatment are important for a successful outcome and there is usually a delay in diagnosis, there is a need to identify early signs and symptoms associated with the definitive diagnosis of disseminated histoplasmosis.

**Materials and methods**

Retrospective cohort study that included patients diagnosed with HIV/AIDS and suspicion of disseminated histoplasmosis in a population attended in a reference hospital in Pereira, Colombia, between January 2015 and May 2018. Clinical and laboratory variables were taken and univariate, bivariate and multivariate analyses were performed to determine the characteristics associated with the diagnosis of disseminated histoplasmosis.

**Results**

Of 147 patients with HIV and clinical suspicion of histoplasmosis, who underwent antigenuria for histoplasma, 37 had positive antigenuria and 110 negative antigenuria. 75.5% were male, 78.9% came from urban areas, 53% had HIV recently diagnosed, while 47% were diagnosed during hospitalisation. The mean haemoglobin was 9.8 mg/dL; CD4 count with a median of 55 cells/mL and viral load of 258.727 copies/mL. The most common clinical manifestations were constitutional symptoms 89.1%, fever 71.4% and weight loss 82.3%. The most common abnormal tests were the presence of anaemia 89.1%, lymphopenia 82.9% and lactate dehydrogenase elevation 44.9%. Other infections were present in 70%, of which the most common was tuberculosis in 69.9%. Of the total population, 69.4% received antifungal therapy and 25.2% died in the hospital. In the bivariate analysis, the conditions associated with the diagnosis of disseminated histoplasmosis were leukocytosis (*p* = 0.001), thrombocytopenia (*p* = 0.001), elevated alkaline phosphatase (*p* = 0.03), AST/ALT ratio (*p* = 0.002) and lactate dehydrogenase elevation (*p* = 0.11). In a logistic regression analysis, the conditions associated were the elevation of alkaline phosphatase (*p* = 0.015) and lactate dehydrogenase elevation (*p* = 0.035), and in a Poison regression analysis the conditions associated with the diagnosis were lactate dehydrogenase elevation (*p* = 0.001).

**Conclusions**

The behaviour of histoplasmosis in our population establishes some differences with the behaviour reported in studies from other regions [3], such as less skin involvement and lymphadenopathy, but with greater haematological manifestations. The adequate analysis of these clinical and laboratory findings can facilitate decision‐making to suspect the presence of disseminated histoplasmosis in patients with HIV/AIDS in our region. According to the high mortality found in this population, new studies are required to determine the factors associated with mortality.

**References**

1. Nacher M, Adenis A, Mc Donald S, Do Socorro Mendonca Gomes M, Singh S, Lopes Lima I, et al. Disseminated Histoplasmosis in HIV‐Infected Patients in South America: A Neglected Killer Continues on Its Rampage. PLoS Negl Trop Dis. 2013;7(11): e2319. Available from: https://doi.org/10.1371/journal.pntd.0002319.

2. Nacher M, Adenis A, Blanchet D, Vantilcke V, Demar M, Basurko C, et al. Risk Factors for Disseminated Histoplasmosis in a Cohort of HIV‐Infected Patients in French Guiana. PLoS Negl Trop Dis. 2014;8(1):e2638. Available from: https://doi.org/10.1371/journal.pntd.0002638.

3. Adenis AA, Aznar C, Couppié P. Histoplasmosis in HIV‐Infected Patients: A Review of New Developments and Remaining Gaps. Curr Trop Med Rep. 2014;1:119. Available from: https://doi.org/10.1007/s40475‐014‐0017‐8.

## P054

### Study of tuberculosis cases registered among prisoners in South Santa Fe, Argentina

**F Biasutti^1^, E Rossi^1^, A Moro^2^, R Huanca^3^, M Lodigiani^3^, E Crochet^3^ and E Careno^1^**

^1^TB/HIV Program, Ministry of Health, Santa Fe, Argentina. ^2^Municipal System of Epidemiology, Ministry of Public Health, Rosario, Argentina. ^3^Ministry of Security, Penitentiary Service of Santa Fe, Santa Fe, Argentina

**Background**

It is widely known that certain infections, such as tuberculosis (TB), present much higher health impact among imprisoned people than in general population. Thus, WHO reported the TB prevalence for prisoners is 100 times higher than in the general population [1]. Several factors, like socio‐economically disadvantages or overcrowding in prisons, lead this particularly vulnerable population to a critic condition with an increased risk to their health.

**Materials and methods**

The present work studied TB cases notified among prisoners of the six centres of detention of South Santa Fe region (SSF), Argentina; where 4250 people (4145 men and 105 women) have been imprisoned during the analysis period (January 2017 to May 2018). TB diagnosis and treatment is systematically controlled by the medical staff of the prison units.

**Results**

During the studied period, 35 cases of TB (0.8% of studied population) were notified, being 25 (71.4%) pulmonary TB; 21 (84.0%) presented positive bacilloscopies, with an average of 2 + + at the diagnosis; 90.0% were new cases or relapses and 3 (8.6%) presented also HIV infection. This number of TB cases would represent an estimated notification rate of 823.5 cases per 100,000 persons, which is much higher than the TB notification rate for the corresponding population in the same area (16.1 per 100,000 inhabitants). Only one case was classified as multiresistant TB. Among the treated patients, a 67.0% presented negative bacilloscopies after two months of treatment. A 77.1% of the treated patients reached the cured/completed treatment condition, with a mean of 7.75 months (Figure 1).

**Conclusions**

TB infection among prisoners is a critic health problem which requires a proactive attitude by the medical staff to improve the epidemiological situation in prisons. In SSF, the immediate aim to achieve is carry out an active information campaign to make prisoners aware of the problem and their possibilities of treatment and cure.

Fuente: SNVS (National Health Surveillance System)

Abstract P054‐Figure 1. Distribution of patients with TB, according to the final treatment condition. Penitentiary Service Santa Fe South Region. Period: 01/01/2017 to 01/01/2018. (n = 35).

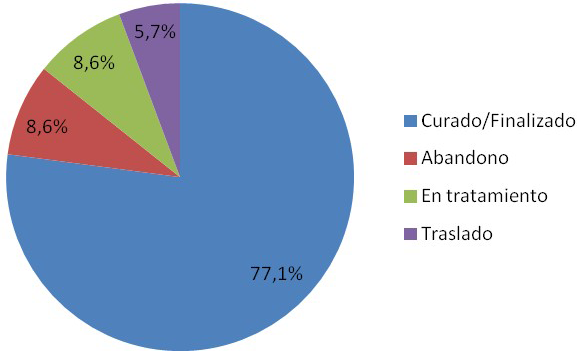

**Reference**

1. Tuberculosis in prisons. WHO. Available from: https://www.who.int/tb/areas‐of‐work/population‐groups/prisons‐facts/en/.

## Treatment, Prevention, Strategies and Outcomes

## P055

### DTG vs LPV/r (DAWNING): efficacy by baseline NRTI resistance and second‐line NRTI use

**D Brown^1^, R Wang^2^, M Underwood^3^, J Hopking^4^, M Nascimento^5^, M Aboud^6^ and J Sievers^5^**

^1^ViiV Healthcare, Abbotsford, Australia. ^2^Clinical Development, ViiV Healthcare, Research Triangle Park, NC, USA. ^3^Virology, ViiV Healthcare, Research Triangle Park, NC, USA. ^4^Statistics, GlaxoSmithKline, Stockley Park, United Kingdom. ^5^Clinical Development, ViiV Healthcare, Brentford, United Kingdom. ^6^Medical Affairs, ViiV Healthcare, Brentford, United Kingdom

**Background**

DAWNING is a non‐inferiority study comparing dolutegravir (DTG) + two nucleoside reverse transcriptase inhibitors (NRTIs) with lopinavir/ritonavir (LPV/r) + two NRTIs in HIV‐1‐infected adults failing first‐line therapy (HIV‐1 RNA ≥400 c/mL) of a non‐NRTI (NNRTI) + 2 NRTIs.

**Materials and methods**

Participants were randomised (1:1, stratified by screening HIV‐1 RNA and number of fully active NRTIs) to 52 weeks of open‐label treatment with DTG or LPV/r + two investigator‐selected NRTIs, including ≥1 fully active NRTI based on screening resistance testing. The primary endpoint was the proportion of participants with HIV‐1 RNA <50 c/mL at Wk48 (Snapshot algorithm). *Post hoc* efficacy analyses were performed based on baseline NRTI resistance profile and NRTI use in the second‐line background regimen (BR).

**Results**

Of 624 participants randomised and treated, 499 (80%) received <2 active NRTIs at baseline. Overall, 84% (261/312) of participants on DTG versus 70% (219/312) on LPV/r achieved HIV‐1 RNA<50 c/mL at Wk8 (adjusted difference 13.8%; 95% confidence interval (CI): 7.3 to 20.3; *p *<* *0.001 for superiority). This difference was consistent regardless of the use of<2 or 2 fully active NRTIs in the BR. NRTI resistance was present in 561 participants (90%) at baseline, M184V/I (alone or plus additional NRTI resistance‐associated mutations (RAMs)) in 513 (82%), K65R in 187 (30%), and ≥1 thymidine analogue mutations (TAMs) in 152 participants (24%). Of participants with M184V/I alone or plus ≥1 NRTI RAMs, 430 participants (84%) took lamivudine (3TC) or emtricitabine (FTC) as part of their BR. Tenofovir disoproxil fumarate (TDF) was included in the BR in the presence of K65R in 15 participants while 86 participants with ≥ 1 TAMs took zidovudine (AZT). Among participants receiving 3TC or FTC in the presence of M184V/I, 85% (187/220) on DTG versus 72% (152/210) on LPV/r had HIV‐1 RNA <50 c/mL at Wk48 (difference 12.6%; 95% CI: 4.9 to 20.3). High responses were also observed in the DTG arm, when AZT or TDF were included in the BR in the presence of TAMs or K65R, respectively; however, participant numbers in these subgroups were small (Table 1).

**Abstract P055‐Table 1. Proportion of participants with HIV‐1 RNA <50 c/mL (Snapshot algorithm) at Week 48 by baseline resistance and NRTI use**

**Table:** 

NRTI used	Baseline NRTI mutations	Treatment	N	Number responded/total assessed	Difference in Proportion (95% CI)
Any	M184V/I only or + ≥1 NRTIs^a^	LPV/r	252	182/252 (72%)	12.1 (5.0, 19.1)
	DTG	261	220/261 (84%)
3TC or FTC + other NRTI	M184V/I only or +≥1 NRTIs^a^	LPV/r	210	152/210 (72%)	12.6 (4.9, 20.3)
	DTG	220	187/220 (85%)
Any	K65R only or + ≥1 NRTIs^b^	LPV/r	92	68/92 (74%)	10.3 (−1.3, 21.9)
	DTG	95	80/95 (84%)
TDF + other NRTI	K65R only or + ≥1 NRTIs^b^	LPV/r	8	7/8 (88%)	−1.8 (−36.4, 32.8)
	DTG	7	6/7 (86%)
AZT + other NRTI	≥1 TAMs	LPV/r	51	40/51 (78%)	7.3 (−8.9, 23.5)
	DTG	35	30/35 (86%)

^a^Including K65R; ^b^Including M184V/I.

**Conclusion**

In DAWNING, response rates were high in participants receiving DTG + 2 NRTIs, regardless of pre‐existing resistance to one of the NRTIs in the BR, including in participants using 3TC or FTC in the presence of M184V/I. In World Health Organization interim guidance on HIV treatment, DTG + 2 NRTIs is now recommended second‐line therapy for patients failing an NNRTI‐based regimen.

## P056

### Initial viral load decline and response rates by baseline viral load strata with dolutegravir plus lamivudine versus dolutegravir plus tenofovir disoproxil fumarate/emtricitabine: pooled results from the GEMINI studies

**P Cahn^1^, J Eron^2^, C Hung^3^, J Baril^4^, J Slim^5^, V Falcó^6^, J Bogner^7^, F Maggiolo^8^, A Mills^9^, J Sievers^10^, C Man^11^, A Currie^12^, M Underwood^13^, A Tenorio^11^, K Pappa^11^, B Wynne^14^, J Koteff^15^, M Gartland^13^, K Smith^16^ and M Aboud^17^**

^1^Infectious Diseases, Fundación Huésped, Buenos Aires, Argentina. ^2^Infectious Diseases, University of North Carolina at Chapel Hill School of Medicine, Chapel Hill, NC, USA. ^3^Infectious Diseases, National Taiwan University Hospital, Taipei, Taiwan. ^4^Family Medicine and Emergency Medicine, Clinique Médicale du Quartier Latin, Montreal, Canada. ^5^Infectious Diseases, Saint Michael's Medical Center, Newark, NJ, USA. ^6^Infectious Diseases, Hospital Vall d'Hebron, Barcelona, Spain. ^7^Clinical Infectiology, Klinikum der Universität München, Munich, Germany. ^8^Infectious Diseases, ASST Papa Giovanni XXIII, Bergamo, Italy. ^9^Men's Health Foundation, Los Angeles, CA, USA. ^10^Clinical Development, ViiV Healthcare, Brentford, United Kingdom. ^11^Clinical Development, ViiV Healthcare, Research Triangle Park, NC, USA. ^12^Statistics, GlaxoSmithKline, Stockley Park, United Kingdom. ^13^Research and Development, ViiV Healthcare, Research Triangle Park, NC, USA. ^14^ID Medicines Discovery and Development, ViiV Healthcare, Research Triangle Park, NC, USA. ^15^Medical Communications, ViiV Healthcare, Research Triangle Park, NC, USA. ^16^Global Research & Medical Strategy, ViiV Healthcare, Research Triangle Park, NC, USA. ^17^International Medical Affairs, ViiV Healthcare, Brentford, United Kingdom

**Background**

At 48 weeks, results from GEMINI‐1/‐2 (NCT02831673; NCT02831764) showed DTG + 3TC was non‐inferior to DTG+TDF/FTC in achieving plasma HIV‐1 RNA <50 c/mL in treatment‐naive adults with baseline HIV‐1 RNA ≤500,000 c/mL. We explored the rapidity of initial VL decline and efficacy responses in those with baseline VL >100,000 c/mL.

**Materials and methods**

Participants (randomised 1:1) received DTG 50 mg + 3TC 300 mg QD or DTG 50 mg+TDF 300 mg/FTC 200 mg QD (stratified by baseline HIV‐1 RNA and CD4+ ). The primary endpoint was proportion of participants with HIV‐1 RNA <50 c/mL at Week 48 (using snapshot algorithm, ITT‐E population), with a 10% non‐inferiority margin. As a *post hoc* analysis, mean change log10‐transformed HIV‐1 RNA from baseline and 95% CIs were calculated at Weeks 4, 8, 12, 16, 24, 36 and 48. Proportions of participants with plasma HIV‐1 RNA <50 c/mL at Week 48 (using snapshot) for the 2DR versus 3DR by baseline HIV‐1 RNA strata ≤100,000, >100,000, >250,000 and >400,000 c/mL were analysed.

**Results**

In the pooled analysis at Week 48, 91% (655/716) of participants in the 2DR versus 93% (669/717) in the 3DR group achieved HIV‐1 RNA <50 c/mL (adjusted treatment difference, −1.7%; 95% CI, −4.4 to 1.1). 20% (140/716) in the 2DR and 21% (153/717) in the 3DR group had baseline HIV‐1 RNA >100,000 c/mL (including 2% with baseline VL >500,000 c/mL). Similar rapid VL log decline was observed in both groups overall (median change from baseline at Week 4: −2.77 and −2.80 log10c/mL in the 2DR and 3DR groups, respectively) and in participants with baseline VL >100,000 c/mL (median change from baseline at Week 4: −3.38 and −3.40 log10c/mL in the 2DR and 3DR groups, respectively). High and similar response rates were seen across baseline VL strata < and >100,000 c/mL. For participants with baseline VL ≤100,000 c/mL, 91% (526/576) in the 2DR versus 94% (531/564) in the 3DR group achieved HIV‐1 RNA <50 c/mL (adjusted treatment difference, −2.8%; 95% CI, −5.8 to 0.2). For participants with baseline VL >100,000 c/mL, 92% (129/140) in the 2DR versus 90% (138/153) in the 3DR group achieved HIV‐1 RNA <50 c/mL (adjusted treatment difference, 1.9%; 95% CI, −4.5 to 8.4). A consistent response pattern was observed in HIV‐RNA strata >250,000 c/mL and >400,000 c/mL.

**Conclusion**

VL decline with DTG + 3TC was rapid and comparable to DTG+TDF/FTC. Response rates in participants with baseline HIV‐1 RNA >100,000 c/mL were high with DTG + 3TC, consistent across strata, including participants with HIV‐1 RNA >400,000 c/mL, and similar to the 3DR. These data demonstrate a high potency of DTG + 3TC, similar to standard care.

## P057

### Clinical and virological outcomes among patients entering into care according to baseline pretreatment NNRTI drug resistance and treatment experience in a nationwide representative survey in Mexico

**Y Caro‐Vega^1^, F Alarid‐Escudero^2^, E Enns^3^, S Sosa‐Rubí^4^, C Chivardi^4^, C Rebolledo^5^, C García‐Morales^6^, G Reyes‐Terán^6^, J Sierra‐Madero^1^ and S Avila‐Rios^6^**

^1^Instituto Nacional de Ciencias Medicas y Nutrición Salvador, Departamento de Infectología, Mexico City, Mexico. ^2^Centro de Investigación y Docencia Economicas, Programa de Politica de Drogas, Aguascalientes, Mexico. ^3^University of Minnesota, Policy Health, Minneapolis, MN, USA. ^4^Instituto Nacional de Salud Pública, Economía de la Salud, Cuernavaca, Mexico. ^5^CENSIDA, Dirección de atención integral, Mexico City, Mexico. ^6^Instituto Nacional de Enfermedades Respiratorias, Centro de Investigación en Enfermedades Infecciosa, Mexico City, Mexico

**Background**

HIV pretreatment drug resistance (PDR) is associated with lower viral suppression (VS) and higher death rates [1]. NNRTI‐PDR in Mexico is approaching 10% [2]. We explored longitudinal associations of PDR in patients entering into care with different outcomes such as remaining in care, loss to follow‐up (LTFU), VS, and death, which are not well described in Mexico.

**Materials and methods**

We analysed PDR and sociodemographic data from 1623 patients of a nationally representative survey carried out in Mexico from 9/2017 to 3/2018 among patients initiating care, with follow‐up data in Mexico's national ART database (SALVAR). Participants were stratified into four groups according to prior ART exposure and presence of NNRTI‐PDR: experienced+resistant, experienced+non‐resistant, naive+resistant and naive+non‐resistant. Proportion of patients remaining in care (recorded as active patients in dataset), LTFU (non‐active patients due to: abandonment, change to security social system, unknown), VS at end of follow‐up (last viral load <200 copies/mL) and death were compared between groups. Multivariate models were developed to assess the relationship between ART exposure and NNRTI resistance and retention in care and VS, adjusted by demographics.

**Results**

From patients in the sample, 18 (1%) experienced+resistant, 185 (11%) experienced+non‐resistant, 147 (9%) naive+resistant and 1273 (78%) naive+non‐resistant. Patients were followed up for a median of 233 days (IQR: 167 to 288). 19% were female, median age was 30 years (IQR: 25 to 38), and median CD4 count 280 cells/mm^3^ (IQR: 136 to 459). In women and heterosexual men, there was a higher proportion of ART‐experienced individuals compared to men and MSM (45% and 25% experienced+resistant, 34% and 21% experienced+non‐resistant, *p* < 0.01 for both). At the end of the follow‐up period, 1373 (84%) patients remained in care. In the experienced+resistant group, 66% of patients remained in care compared to 77% among experienced+non‐resistant, and 86% in naives (*p* < 0.001). Proportion of deaths was 11%, 5%, 3%, and 4% (*p* = 0.22), and LTFU 22%, 14%, 7% and 6% (*p* < 0.01) among experienced+resistant, experienced+non‐resistant, naive+resistant and naive+non‐resistant respectively. Among 1198 individuals with viral load data available, VS was achieved in 47% and 63% resistant and non‐resistant+experienced and 84% and 80% resistant and non‐resistant+naïve groups (*p* < 0.001). After multivariable correction, experienced+resistant patients remained in care at a rate significantly lower (aOR = 0.31, 95% CI: 0.11 to 0.91) and had lower VS (aOR = 0.20; 95% CI: 0.07 to 0.62) compared to naïve+non‐resistant patients (Table 1).

**Abstract P057‐Table 1. Clinical and sociodemographic characteristics according to prior exposure to ART and the presence of NNRTI‐PDR**

**Table:** 

	Experienced+resistant n = 18	Experienced+non‐resistant n = 185	Naïve+resistant n = 147	Naïve+non‐resistant n = 1273	*p* value
Female; n (%)	8 (45%)	63 (34%)	31 (21%)	200 (16%)	<0.01
Median age; years (IQR)	35 (28 to 40)	34 (27 to 41)	30 (25 to 40)	29 (25 to 38)	0.25
Risk of transmission; n (%)					
Heterosexual/others	8 (50%)	66 (38%)	31 (23%)	211 (18%)	<0.01
MSM	4 (25%)	74 (43%)	74 (56%)	757 (64%)	
Heterosexual men	4 (25%)	31 (21%)	28 (21%)	211 (18%)	
Median CD4 count; cells/mm^3^ (IQR)	383 (182 to 534)	239 (115 to 443)	304 (134 to 466)	281 (140 to 458)	0.31
Education; n (%)					
Elementary or lower	8 (44%)	54 (30%)	25 (18%)	189 (15%)	<0.01
High school or higher	10 (56%)	126 (70%)	117 (82%)	1040 (85%)	
Occupation; n (%)					
Employed	4 (22%)	72 (40%)	70 (51%)	651 (54%)	<0.01
Unemployed	14 (78%)	94 (53%)	52 (38%)	444 (37%)	
Student	0 (0%)	12 (7%)	15 (11%)	111 (9%)	

**Conclusions**

Among patients entering into care, the group of ART‐experienced patients especially those with PDR showed the worst clinical outcomes compared to ART naives. This group was enriched with women and persons with lower education and unemployed, which indicates higher levels of social vulnerability.

**References**

1. WHO 2017. Global action plan on HIV drug resistance 2017 to 2021. Available from: http://www.who.int/hiv/pub/drugresistance/hivdr‐action‐plan‐2017‐2021/en/.

2. Ávila‐Ríos S, García‐Morales C, Matías‐Florentino M, et al. HIVDR MexNet Group. Pretreatment HIV‐drug resistance in Mexico and its impact on the effectiveness of first‐line antiretroviral therapy: a nationally representative 2015 WHO survey. Lancet HIV. 2016;3(12):e579‐e591. https://doi.org/10.1016/s2352‐3018(16)30119{00ENDSH00}9.

## P058

### Outcomes with continuation of tenofovir–lamivudine versus switching to zidovudine–lamivudine in second‐line antiretroviral therapy in Haiti

**S Pierre^1^, I Bocharova^2^, C Nguyen^2^, P Cremieux^2^, S Koenig^3^ and J Pape^1^**

^1^Medicine, GHESKIO, Port‐au‐Prince, Haiti. ^2^Analysis Group, Boston, MA, USA. ^3^Medicine, Brigham and Women's Hospital, Boston, MA, USA

**Background**

The World Health Organization guidelines recommend switching the nucleoside reverse transcriptase inhibitor (NTRI) backbone, so that patients who fail tenofovir (TDF)/lamivudine (3TC) should be switched to zidovudine (AZT)/3TC. However, due to the toxicity of AZT and the demonstrated efficacy of NRTIs in spite of genotypic resistance in several recent studies, many providers in resource‐poor settings continue TDF/3TC in second‐line ART, after first‐line failure.

**Materials and methods**

This retrospective cohort study included all patients at GHESKIO in Port‐au‐Prince, Haiti, who failed first‐line efavirenz (EFV)/TDF/3TC and switched to a second‐line regimen that included a ritonavir‐boosted protease inhibitor (bPI), in combination with either TDF/3TC or AZT/3TC. Outcomes for patients who continued TDF versus switched to AZT were compared with the Chi Square test.

**Results**

A total of 1017 patients who failed first‐line EFV/TDF/3TC was included in the study. After documentation of first‐line failure, 733 (72%) continued TDF/3TC in combination with a bPI for second‐line treatment (continuation group), and 284 (28%) switched to AZT/3TC/bPI (switch group). More patients in the switch group were male (55% vs. 48%; *p* = 0.048), and fewer in the switch group were single (19% vs. 25%; *p* = 0.029), compared with the continuation group. Patients in the switch group were more likely to have started second‐line ART in earlier years (*p* < 0.001); 69% started second‐line ART from 2012 to 2016, and 31% in 2017 to 2018. Among patients in the TDF continuation group, 42% started second‐line treatment in 2012 to 2016, and 58% in 2017 to 2018. The median follow‐up time on second‐line ART was shorter for the TDF continuation group, compared with the group that switched to AZT (8.2 months vs. 17.9 months). Overall, 78% of patients were retained in care, and 16% were lost to follow‐up, 4% died, 1% transferred and 1% started a third‐line regimen. Loss to follow‐up was higher for the group that switched to AZT versus the group that continued TDF (21% vs. 15%; *p* = 0.014), and retention was lower for those who switched versus those who continued TDF (72% vs. 81%; *p* = 0.003). More patients who switched to AZT received a viral load after starting second‐line ART (82% vs. 60%; 0.004), but the proportion of patients with viral suppression was higher in the TDF continuation group (60% vs. 46%; *p* = 0.004).

**Conclusions**

In recent years, due to evidence indicating NRTI efficacy in spite of genotypic resistance, more patients are continuing TDF/3TC than switching to AZT/3TC for second‐line treatment in Port‐au‐Prince. Preliminary data suggest that treatment outcomes are not inferior with this approach.

## P059

### Access to PrEP for MSM: opportunities and challenges, Brazil, 2018

**D Calixto, A Pati Pascom, I Pereira, N Araújo, G Silva, A Schwartz Benzaken and G Fernando Mendes Pereira**

Department of Surveillance, Prevention and Control of STIs, HIV/AIDS and Viral Hepatitis, Ministry of Health of Brazil, Brasília, Brazil

**Background**

In Brazil, pre‐exposure prophylaxis (PrEP) free‐of‐charge at the public health system has gained a central role in combination prevention of HIV infection. PrEP is recommended for HIV prevention for most at‐risk MSM by the Public Health System (SUS). The aim of this study is to report the profile and characteristics of MSM using PrEP during the first year of PrEP implementation as public health.

**Materials and methods**

Programmatic PrEP data from three forms for PrEP monitoring were analysed for this study: First Service Record, First Return Record and Clinical Monitoring Record as well as the sociodemographic data collected from PrEP user registration from the Medicine Logistic Control System (Siclom), collected from 1 January to 31 December 2018.

**Results**

Out of the 6733 PrEP users in December 2018, 83% (5567) were MSM (Figure 1). A similar distribution pattern is noted in most all cities of Brazil where PrEP is implemented, having MSM as the majority of users. 17% were between 18 and 29 years and 40% were 30 and 39 years. Approximately 60% self‐declared themselves as White and 40% as Black. Considering education, 84% of the MSM have at least 12 years of study. Regarding sexual practices, 49% reported a decrease in the number of partnerships and 24% reported an increase in condom use.

**Conclusions**

Free‐of‐charge PrEP in the public health system can reach a large number of MSM, but access also needs to be equitable considering intersectional risk factors. Our analyses suggest the need to invest in community‐led strategies to increase the reach of PrEP among younger, Black and less educated MSM.

Abstract P059‐Figure 1. PrEP users by category, Brazil, January to December 2018.

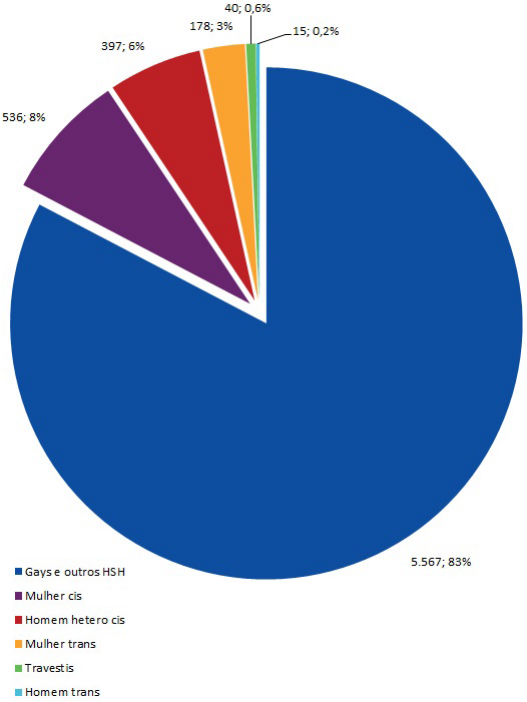

## P060

### Durable suppression and low rate of virologic failures three years after switch to DTG+RPV 2‐drug regimen: SWORD 1 & 2 studies

**M Aboud^1^, J van Wyk^1^, C Orkin^2^, R Rubio^3^, J Bogner^4^, D Baker^5^, M Khuong‐Josses^6^, D Parks^7^, K Angelis^8^, L Kahl^9^, J Matthews^10^, M Underwood^11^, B Wynne^12^, M Nascimento^9^, K Vandermeulen^13^, M Gartland^14^ and K Smith^15^**

^1^Medical Affairs, ViiV Healthcare, Brentford, United Kingdom. ^2^HIV and HIV/Hep C Research, Queen Mary University of London, London, United Kingdom. ^3^Internal Medicine, Hospital 12 de Octubre, Madrid, Spain. ^4^Infectious Diseases, Hospital of the University of Munich, Munich, Germany. ^5^HIV Medicine, East Sydney Doctors, Darlinghurst, Sydney, Australia. ^6^Infectious Diseases, CHG – Hôpital Delafontaine, Saint Denis Cedex, France. ^7^Internal Medicine, Central West Clinical Research, St Louis, MO, USA. ^8^Statistics, GlaxoSmithKline, Stockley Park, United Kingdom. ^9^Clinical Development, ViiV Healthcare, Brentford, United Kingdom. ^10^Clinical Development, ViiV Healthcare, Research Triangle Park, NC, USA. ^11^Virology, ViiV Healthcare, Research Triangle Park, NC, USA. ^12^Dolutegravir, ViiV Healthcare, Research Triangle Park, NC, USA. ^13^Global Regulatory, Janssen Pharmaceutica NV, Beerse, Belgium. ^14^Medicine Development, ViiV Healthcare, Research Triangle Park, NC, USA. ^15^Global Medical Strategy, ViiV Healthcare, Research Triangle Park, NC, USA

**Background**

SWORD 1 and 2 studies demonstrated efficacy of dolutegravir (DTG) + rilpivirine (RPV), a two‐drug regimen for maintenance of virologic suppression, that was non‐inferior to continuing current antiretroviral regimen (CAR; 3‐drug regimen) at Week 48. Data through Week 100 demonstrated maintenance of high‐level suppression, low rates of virologic failures with few non‐nucleoside reverse transcriptase inhibitor (NNRTI) resistance‐associated mutations (RAMs), no integrase strand transfer inhibitor (INSTI) RAMs, and improvements in bone and renal biomarkers. Here, we present results through Week 148.

**Materials and methods**

Two identical open‐label, global, phase III, non‐inferiority studies evaluated efficacy and safety of switching from CAR to DTG+RPV once daily in HIV‐1‐infected adults, with viral load <50 c/mL for >6 months and no history of virologic failure. Participants were randomised 1:1 to switch to DTG+RPV (early switch (ES) group) or continue CAR. Participants’ randomised to CAR with confirmed suppression at Week 48 switched to DTG+RPV at Week 52 (late switch (LS) group). Efficacy by Snapshot, virology and safety endpoints were evaluated through Week 148 for the intent‐to‐treat–exposed and safety populations, respectively.

**Results**

The studies randomised and exposed 1024 participants (DTG+RPV 513; CAR 511). Efficacy and key safety for the ES and LS DTG+RPV groups are shown in Table 1. Confirmed virologic withdrawal (CVW) criterion was low in both groups: ES DTG+RPV 8 (2%) and, LS DTG+RPV 3 (<1%). The safety profile of the LS DTG+RPV group following 96 weeks of DTG+RPV at Week 148 was comparable to the ES group at Week 100. No INSTI resistance was observed, with limited resistance to RPV (n = 5; 0.5%) observed; 1 of these participants had pre‐existing NNRTI RAMs.

**Conclusions**

Switching participants from any three‐drug to two‐drug regimen of DTG+RPV was associated with maintenance of viral suppression, low frequency of CVWs, a very limited number of NNRTI RAMs and no INSTI RAMs over treatment for three years in the ES group and two years in the LS group. DTG+RPV has demonstrated durable efficacy, is well tolerated and offers a HIV treatment option with less cumulative antiretroviral exposure.

**Abstract P060‐Table 1. Pooled SWORD 1 and 2 efficacy and key safety results at Week 148**

**Table:** 

Outcome, n (%)	Early Switch DTG+RPV (N = 513)			Late Switch DTG+RPV (N = 477)^a^	
	Day 1 to Week 48	Day 1 to Week 100^b^	Day 1 to Week 148	Week 52 to Week 100^b^	Week 52 to Week 148
Virologic success	486 (95)	456 (89)	432 (84)	444 (93)	428 (90)
Virologic non‐response	3 (<1)	13 (3)	14 (3)	10 (2)	11 (2)
No virologic data^c^	24 (5)	44 (9)	67 (13)	23 (5)	38 (8)
Key safety					
AEs leading to withdrawal	21 (4)	34 (7)	42 (8)	15 (3)	19 (4)
Drug‐related Grade 2 to 4 AEs	29 (6)	29 (6)	31 (6)	13 (3)	16 (3)
Serious AE	27 (5)	58 (11)	71 (14)	30 (6)	43 (9)
Drug‐related serious AEs	4 (<1)	4 (<1)	4 (<1)	0	0

^a^34 participants randomised to CAR did not meet the criterion to switch to DTG+RPV at Week 52; ^b^AEs reported from analysis of the safety population who received ≥1 dose of DTG+RPV represents cumulative data through to the data cutoff date for the Week 100 analysis; ^c^No virologic data due to discontinuation due to AE or death, discontinuation for other reasons or missing data during window but on study.

## P061

### Efficacy of dolutegravir (DTG) plus lamivudine (3TC) versus DTG plus tenofovir/emtricitabine (TDF/FTC) in antiretroviral treatment‐naive adults with HIV‐1 infection: 48‐week subgroup results from the GEMINI studies in Latin American participants

**N Porteiro^1^, J Andrade‐Villanueva^2^, P Cahn^3^, J Madero^4^, C Man^5^, J Sievers^6^, R Urbaityte^7^, D Brown^8^, I Prudente^9^ and M Aboud^10^**

^1^Infectious Diseases, Fundacion IDEAA, Buenos Aires, Argentina. ^2^Investigación en Inmunodeficiencias, Instituto de Investigación en Inmunodeficiencias y VIH, Centro Universitario de Ciencias de la Salud, Universidad de Guadalajara, Jalisco, Mexico. ^3^Infectious Diseases, Fundación Huésped, Buenos Aires, Argentina. ^4^Internal Medicine, Instituto Nacional de Ciencias Médicas y Nutrición Salvador Zubirán, Mexico City, Mexico. ^5^Clinical Development, ViiV Healthcare, Research Triangle Park, NC, USA. ^6^Clinical Development, ViiV Healthcare, Brentford, United Kingdom. ^7^Statistics, GlaxoSmithKline, Stockley Park, United Kingdom. ^8^International, ViiV Healthcare, Abbotsford, Australia. ^9^Medical Affairs, ViiV Healthcare, Santiago, Chile. ^10^Medical Affairs, ViiV Healthcare, Brentford, United Kingdom

**Background**

The requirement for lifelong antiretroviral therapy of HIV infection has highlighted interest in two‐drug regimens (2DRs) to minimise cumulative drug exposure. In the GEMINI‐1/‐2 studies, the 2DR of dolutegravir (DTG) plus lamivudine (3TC) was recently shown to be non‐inferior at Week 48 compared with the three‐drug regimen DTG + tenofovir disoproxil fumarate (TDF)/emtricitabine (FTC) in achieving plasma HIV‐1 RNA <50 c/mL in treatment‐naive adults [1].

**Materials and methods**

GEMINI‐1/‐2 are identical, global, double‐blind, multicentre, phase III studies evaluating the efficacy and safety of DTG + 3TC once‐daily in treatment‐naive HIV‐1‐infected adults with screening HIV‐1 RNA ≤500,000 c/mL (NCT02831673/NCT02831764). Participants were randomised 1:1 (stratified by screening plasma HIV‐1 RNA and CD4+ cell count) to treatment with DTG + 3TC or DTG+TDF/FTC. The primary endpoint was the proportion of participants with plasma HIV‐1 RNA <50 c/mL at Week 48 (snapshot algorithm). A secondary analysis was conducted to look at outcomes in participants recruited from Latin American centres.

**Results**

A total of 1433 adults were randomised and treated across the 2 GEMINI studies, including 307 (21%) participants treated at 18 sites from 3 Latin American countries (Argentina, Mexico, Peru). Based on a 10% non‐inferiority margin, DTG + 3TC was non‐inferior to DTG+TDF/FTC at Week 48 in both GEMINI‐1/‐2 and the pooled analysis [1]. Responses were high and generally consistent in participants from Latin American countries, noting the small patient numbers in individual countries (Table 1). Across the full population for both studies, 6 participants on DTG + 3TC and 4 on DTG+TDF/FTC met protocol‐defined virologic withdrawal criteria through Week 48; none had treatment‐emergent primary integrase strand transfer inhibitor or nucleoside reverse transcriptase inhibitor resistance mutations. Overall rates of adverse events (AEs) were similar between arms, with low rates of AEs leading to withdrawal for both DTG + 3TC and DTG+TDF/FTC (2% each arm). More drug‐related AEs were reported with DTG+TDF/FTC. Across Latin American participants, low rates of AEs leading to withdrawal were observed at Week 48 – 2 participants per treatment arm (<1% – and was consistent with the overall study population.

**Conclusions**

DTG + 3TC demonstrated non‐inferior efficacy to DTG+TDF/FTC in treatment‐naive adults with screening HIV‐1 RNA ≤500,000 c/mL at Week 48. Both regimens were well tolerated. Subgroup analysis of efficacy in participants’ randomised and treated across Latin American sites was generally consistent with overall study results. These results further demonstrate DTG + 3TC as an option for initial treatment of HIV infection across different geographies, including Latin America. The studies are ongoing to explore long‐term durability/safety.

**Abstract P061‐Table 1. Proportion of participants with plasma HIV‐1 RNA <50 c/mL at Week 48: snapshot analysis – ITT‐E population**

**Table:** 

Pooled GEMINI‐1/‐2 population/Country	Arm	Snapshot responders (n/N)	Snapshot responders, %
Total study population	DTG + 3TC	655/716	91
	DTG+TDF/FTC	669/717	93
	Adjusted difference (95% CI): −1.7 (−4.4, 1.1)		
Argentina	DTG + 3TC	85/91	93
	DTG+TDF/FTC	88/91	97
	Unadjusted difference (95% CI): −3.3 (−9.6, 3.0)		
Mexico	DTG + 3TC	47/50	94
	DTG+TDF/FTC	48/52	92
	Unadjusted difference (95% CI): 1.7 ( −8.1, 11.5)		
Peru	DTG + 3TC	9/11	82
	DTG+TDF/FTC	11/12	92
	Unadjusted difference (95% CI): −9.8 (−37.5, 17.8)		
Total LATAM	DTG + 3TC	141/152	93
	DTG+TDF/FTC	147/155	95
	Unadjusted difference (95% CI): −2.1 (−7.5, 3.3)		

**Reference**

1. Cahn, et al. GEMINI Study Team. Lancet. 2019;393:143–55.

## P062

### Long‐term B/F/TAF switch efficacy in patients with archived pre‐existing resistance

**K Andreatta^1^, M Willkom^1^, R Martin^2^, S Chang^1^, H Liu^3^, Y Liu^3^, H Graham^4^, H Martin^3^, C Falistocco^5^, M Mora^6^ and K White^1^**

^1^Clinical Virology, Gilead Sciences, Foster City, CA, USA. ^2^Public Health & Medical Affairs, Gilead Sciences, Foster City, CA, USA. ^3^Biostatistics, Gilead Sciences, Foster City, CA, USA. ^4^Clinical Research, Gilead Sciences, Foster City, CA, USA. ^5^Public Health & Medical Affairs, Gilead Sciences, Buenos Aires, Argentina. ^6^Public Health & Medical Affairs, Gilead Sciences, São Paulo, Brazil

**Background**

Studies 1844 and 1878 demonstrated non‐inferior efficacy of switching suppressed HIV‐1‐infected adults to bictegravir/emtricitabine/tenofovir alafenamide (B/F/TAF) versus continuing dolutegravir/abacavir/lamivudine (DTG/ABC/3TC) or boosted protease inhibitor (PI)‐based regimens. At week 48, 93% in the B/F/TAF groups versus 95% in the ABC/DTG/3TC group and 89% in the PI group had HIV‐1 RNA <50 copies/mL by snapshot algorithm, after which B/F/TAF treatment continued open‐label. Here, we present resistance analyses and virologic outcomes after two years of B/F/TAF treatment.

**Materials and methods**

Archived pre‐existing HIV‐1 drug resistance was assessed by historical genotypes (documented resistance to study drugs was exclusionary) and retrospective baseline proviral DNA genotyping (participants with resistance to study drugs detected post‐randomisation were allowed to remain on study). Virologic outcomes were based on last available on‐treatment HIV‐1 RNA.

**Results**

Altogether, 572 participants switched to B/F/TAF and were treated for a median of 108 weeks (IQR 106 to 118 weeks). Pre‐switch reverse transcriptase (RT) genotypic data were available for 78% (447/572) of B/F/TAF‐treated participants; integrase data were available for 55% (314/572). Pre‐existing primary NRTI resistance (‐R), NNRTI‐R and INSTI‐R substitutions were observed in 16% (71/447), 21% (93/447) and 1.9% (6/314) respectively. High frequencies of NRTI‐R substitutions M184V or M184I (9.8%, 44/447) and thymidine analogue mutations (TAMs; 8.5%, 38/447) were detected by DNA genotyping. Substitutions associated with resistance to the NNRTI rilpivirine (RPV) were observed in 9.6% (43/447). At the time of analysis, 99% (564/572) of B/F/TAF‐treated participants were suppressed (HIV‐1 RNA <50 copies/mL), including 95% (42/44) with archived M184V/I, 95% (36/38) with TAMs, 98% (42/43) with RPV‐R, and 100% (6/6) with INSTI‐R. There was no resistance development in B/F/TAF‐treated participants through week 48, and no participants met criteria for resistance testing after week 48.

**Conclusions**

Pre‐existing RT resistance was common among suppressed participants switching to B/F/TAF, notably RPV‐R and previously unidentified M184V/I and TAMs. High rates of virologic suppression were observed in the overall and drug resistant populations through 108 weeks of B/F/TAF treatment with no resistance development, indicating that B/F/TAF is a durable switch option for suppressed patients, including those with evidence of these pre‐existing NNRTI and NRTI resistance.

## P063

### Brazilian challenge: reduction of the time between first CD4+ and early antiretroviral therapy (ART)

**A Kolling, M Camelo Madeira de Moura, R Vianna Brizolara, A Sposito Tresse, F de Barros Perini, A Pati Pascom and G Fernando Mendes Pereira**

Department of STI, AIDS and Viral Hepatitis, Ministry of Health, Brasília, Brazil

**Background**

In 2017, 42,420 new cases of HIV and 37,791 cases of AIDS were diagnosed in Brazil. Despite the adoption of treatment for all in 2013, early diagnosis and treatment of HIV remain as challenges in the country. In order to evaluate the access and quality of the PLWHIV care, it is proposed to evaluate the time between the first CD4+ test and the initiation of ART.

**Materials and methods**

Included in this study are all the PLWHIV who had at least one CD4 test within the criteria for eligibility of ART defined for each year: 2009 to 2012, ≤350 cells/mm^3^; 2013, ≤500 cells/mm^3^, and 2014 to 2018, was adopted “treatment for all.” The measurement used: (data of first dispensation) – (data of first request for CD4+ ). The period of analysis was 01/2009 to 09/2018.

**Results**

Analysing the time between the first CD4+ and the beginning of ART, over the years (2009 to 2018), there are periods of falling with oscillations. In 2009, when Brazilian protocol recommended the initiation of therapy by individuals with CD4 ≤350 cells/mm^3^, 38% diagnosed PLWHIV started ART after six months since first CD4+. In this period, there was a significant increase in number of PLWHIV who started treatment less than one month, 29% in 2009 and 51% in 2018 [1] (Figure 1).

**Conclusions**

Even after “treatment for all,” Brazil continues with an annual increase of more than 40,000 new diagnoses. Initiatives such as testing, decentralisation and treatment in primary care services, and adoption of strategies to linkage and retention of these people to services has progressively reduced the time between first CD4+ and ART initiation over the years. The challenge now is to support earlier initiation of ART for PLWHIVs with the goal of beginning ART in the same day and avoiding AIDS and other comorbidities.

Abstract P063‐Figure 1. Proportion of PLWHIV of 18 years old or more according to ART initiation per year of first CD4+. Brazil, 2009 to 2018.

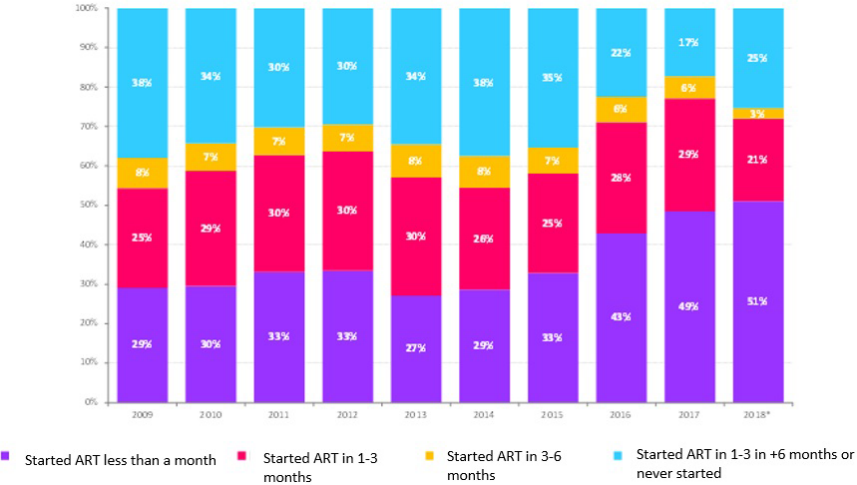

**Reference**

1. BRAZIL. Ministry of Health. Secretariat of Health Surveillance. Department of Sexually Transmitted Infections, AIDS and Viral Hepatitis. Clinical Monitoring Report of HIV, 2018.

## P064

### Renal safety of TAF versus TDF in a pooled analysis of 26 phase 2/3 clinical trials

**S Gupta^1^ , F Post^2^, J Arribas^3^, D Podzamczer^4^, A Clarke^5^, E Negredo^6^, S Guo^7^, L Zhong^7^, C Carter^8^, H Martin^8^, D SenGupta^8^, R Campo^9^ and M Das^8^**

^1^Department of Medicine, Indiana University School of Medicine, Indianapolis, IN, USA. ^2^West Education Centre, Kings College Hospital NHS Foundation, London, United Kingdom. ^3^Instituto de Investigación, Hospital Unversitario La Paz, Madrid, Spain. ^4^Infectious Diseases Service, Hospital Universitario de Bellvitge, Barcelona, Spain. ^5^Sexual Health and Clinical Trials Unit, Royal Sussex County Hospital, Brighton, United Kingdom. ^6^Department of Medicine, Universitat de Vic‐Universitat Central de Catalunya, Barcelona, Spain. ^7^Biostatistics, Gilead Sciences, Inc., Foster City, CA, USA. ^8^Clinical Research, Gilead Sciences, Inc., Foster City, CA, USA. ^9^Public Health and Medical Affairs, Gilead Sciences, Inc., Miami, FL, USA

**Background**

Compared with tenofovir disoproxil fumarate (TDF), tenofovir alafenamide (TAF) has been associated with improvement in markers of renal dysfunction in individual randomised trials; however the comparative incidence of clinically significant renal events remains unclear. We used a pooled data approach to increase the person‐years of drug exposure analysed, maximising our ability to detect differences in clinically significant outcomes.

**Materials and methods**

We pooled clinical renal safety data across 26 treatment naïve and antiretroviral switch studies in order to compare the incidence of proximal renal tubulopathy (PRT) and discontinuation due to renal adverse events (AEs) between participants taking TAF‐containing regimens versus those taking TDF‐containing regimens. We performed secondary analyses from seven large randomised studies (two treatment‐naïve and five switch studies) to compare incidence of renal AEs, treatment‐emergent proteinuria, changes in serum creatinine, creatinine clearance, and urinary biomarkers (albumin, beta‐2‐microglobulin and retinol binding protein to creatinine ratios).

**Results**

Our integrated analysis included 9322 adults and children with HIV (n = 6360 TAF, n = 2962 TDF) with exposure of 12,519 person‐years to TAF and 5947 to TDF. There were no cases of PRT in participants receiving TAF versus 10 cases in those receiving TDF (*p* < 0.001), and fewer individuals on TAF (3/6360) versus TDF (14/2962) (*p* < 0.001) discontinued due to a renal AE. Participants initiating TAF‐ versus TDF‐based regimens had more favourable changes in renal biomarkers through 96 weeks of therapy.

**Conclusion**

These pooled data from 26 studies, with over 12,500 person‐years of follow‐up in children and adults, support the comparative renal safety of TAF over TDF.

## P065

### A phase 3b, open‐label, pilot study to evaluate switching to elvitegravir/cobicistat/emtricitabine/tenofovir alafenamide (E/C/F/TAF) in virologically suppressed HIV‐1 infected adult subjects harbouring the NRTI resistance mutation M184V and/or M184I (GS‐US‐292‐1824)

**I Perez‐Valero^1^, J Llibre^2^, A Lazzarin^3^, J Molina^4^, N Margot^5^, D Piontkowsky^5^, M Das^5^, L Espinoza^6^ and R Haubrich^5^**

^1^Unidad VIH, Hospital Universitario, Madrid, Spain. ^2^Fundacion Lucha contra el Sida, Madrid, Spain. ^3^Fondazione IRCCS, San Rafaelo del Monte Tabor, Milan, Italy. ^4^Infectious Diseases, Saint‐Louis Hospital and University, Paris, France. ^5^Gilead Sciences Inc., Foster City, CA, USA. ^6^Public Health and Medical Affairs, Gilead Sciences, Inc, Miami, FL, USA

**Background**

Treatment with once‐daily E/C/F/TAF in HIV‐1‐infected therapy‐naïve patients was shown to be effective and safe through 144 weeks in two randomised, double‐blinded trials, which excluded participants whose HIV‐1 harboured the M184V and/or M184I mutation.

**Materials and methods**

This ongoing, prospective open‐label, single arm, multicentre, 48‐week trial is evaluating the efficacy and safety of switching suppressed participants to E/C/F/TAF from a stable regimen (≥6 months) of a third agent plus either F/tenofovir disoproxil fumarate or abacavir/lamivudine. Participants had a historical genotype report showing M184V and/or M184I and no evidence of previous virologic failure (VF) or resistance to boosted PIs or INSTIs. At screening, HIV‐1 RNA <50 c/mL was required as well as the absence of additional NRTI or PI resistance mutations based on sequencing of integrated HIV DNA (GenoSure Archive, Monogram Biosciences). The primary objective is to evaluate the efficacy of switching to E/C/F/TAF in maintaining HIV‐1 RNA <50 c/mL at Week 12 using pure virologic response (PVR). Participants with discontinuation or missing values were considered responders if they never had HIV‐1 RNA >50 c/mL at 2 consecutive visits and the last HIV‐1 RNA was <50 c/mL. This report presents the Week 24 data.

**Results**

Thirty‐seven participants were enrolled and switched to E/C/F/TAF. The mean age was 50 years (range 22 to 76), 73% White, 19% Black, 22% women, median CD4 count 724 cells/μL and 100% HIV‐RNA <50 c/mL at baseline. Through Week 24, all 37 participants (100%) had HIV‐1 RNA <50 c/mL based on PVR. Three participants who discontinued prior to Week 24 with last recorded HIV‐1 RNA <50 c/mL were not considered VF. Four serious adverse events occurred (none were study drug‐related): one each of squamous cell carcinoma, acute kidney injury (with poorly controlled hypertension and diabetes), transient proteinuria (resolved on study drug) and pulmonary embolism. Twenty‐two per cent (8/37) of participants experienced a study drug‐related AE (grade 1 or 2); one participant discontinued due to grade 2 muscle spasms (Table 1).

**Conclusions**

E/C/F/TAF offers an effective, well‐tolerated switch option for patients with pre‐existing M184V and/or M184I mutations. These data on continued virologic suppression despite resistance are encouraging though longer term data are needed.

**Abstract P065‐Table 1.**

**Table:** 

Baseline characteristics	
Presence of M184V, M184I or both mutations, screening historic genotype	100% (37/37)
Baseline archive DNA mutations: M184V/I, M184V/I + NNRTI, NNRTI only, none	22%, 22%, 5%, 51%
Regimen prior to switching:	
2 NRTI + PI + ritonavir or cobicistat	54%
2 NRTI + INSTI, 2 NRTI + INSTI + NNRTI	32%, 3%
2 NRTI + NNRTI	11%
Results	
Week 24 HIV‐1 RNA <50 c/mL (PVR)	100% (37/37)
Week 24 HIV‐1 RNA <50 c/mL (missing = failure)	92% (34/37)
Week 24 HIV‐1 RNA <50 c/mL (missing = excluded)	100% (34/34)
Virological failures or emergent resistance	0

## P066

### A phase 3, randomised, controlled clinical trial of bictegravir in a fixed‐dose combination, B/F/TAF, versus ABC/DTG/3TC in treatment‐naïve adults at Week 96

**D Wohl^1^, Y Yazdanpanah^2^, A Baumgarten^3^, A Clarke^4^, D Hagins^5^, M Ramgopal^6^, K White^7^, S Collins^7^, R Chirino^8^, F Silva^8^ and H Martin^7^**

^1^University of North Carolina at Chapel Hill, Chapel Hill, NC, USA. ^2^Hôpital Bichat Claude Bernard, Paris, France. ^3^Zentrum für Infektiologie Berlin Prenzlauer Berg (ZIBP), Berlin, Germany. ^4^Royal Sussex County Hospital, Brighton, United Kingdom. ^5^Central Texas Clinical Research, Austin, TX, USA. ^6^Midway Immunology Center, Fort Pierce, FL, USA. ^7^Gilead Sciences Inc, Foster City, CA, USA. ^8^Gilead Sciences Inc, Public Health & Medical Affairs, Mexico City, Mexico

**Introduction**

Bictegravir (B), a potent INSTI with a high barrier to resistance, is coformulated with emtricitabine (F) and tenofovir alafenamide (TAF) as the FDA‐approved single‐tablet regimen B/F/TAF. We report Week (W) 96 results from an ongoing phase 3 study comparing B/F/TAF to coformulated dolutegravir, abacavir, and lamivudine (DTG/ABC/3TC) in treatment‐naïve adults living with HIV‐1. Primary outcome at W48 demonstrated non‐inferior virologic responses, similar bone and renal profiles, and no viral resistance.

**Materials and methods**

We randomised 1:1 HLA‐B*5701‐negative adults, without HBV and with estimated glomerular filtration rate (eGFR) ≥50 mL/min to receive blinded B/F/TAF (50/200/25 mg) or DTG/ABC/3TC (50/600/300 mg) with matching placebos QD. Primary endpoint was proportion with HIV‐1 RNA <50 c/mL at W48 (FDA snapshot), with secondary analyses at W96. Non‐inferiority was assessed with 95% confidence intervals (CI) (12% margin). Other secondary endpoints were safety (adverse events (AEs), laboratory abnormalities) and predefined analyses of bone mineral density (BMD) and measures of renal function (eGFR, proteinuria).

**Results**

In total, 629 adults were randomised/treated (314 B/F/TAF, 315 DTG/ABC/3TC). At W96, B/F/TAF was non‐inferior to DTG/ABC/3TC: 87.9% versus 89.8%, respectively, achieved HIV‐1 RNA <50 c/mL (difference −1.9%; 95% CI −6.9% to 3.1%, *p* = 0.45). In per‐protocol analysis, 99.6% on B/F/TAF versus 98.9% on DTG/ABC/3TC achieved HIV‐1 RNA <50 c/mL (*p* = 0.33). Most common AEs overall were nausea (11% B/F/TAF, 24% DTG/ABC/3TC, *p* < 0.001), diarrhoea (15%, 16%) and headache (13%, 16%). Through W96, no participant had emergent resistance to study drugs. No participant discontinued B/F/TAF due to AEs; 5 (2%) discontinued DTG/ABC/3TC due to AEs (1 after W48). Treatment‐related AEs occurred in 28% B/F/TAF versus 40% DTG/ABC/3TC (*p* = 0.002); most common was nausea (6%, 17%. *p* < 0.001). At W96, mean % changes in spine and hip BMD were small and similar between groups (Figure 1); median change in eGFR was significantly less with B/F/TAF, while median % changes in proteinuria were similar.

**Conclusions**

At W96, B/F/TAF was virologically non‐inferior to DTG/ABC/3TC, with no viral resistance or safety‐related discontinuations. B/F/TAF was well tolerated with less nausea than DTG/ABC/3TC and similar bone and renal safety.

Abstract P066‐Figure 1. Mean % changes in spine and hip BMD through W96. CI, Confidence interval. *Comparison of B/F/TAF versus DTG/ABC/3TC at Week 96 by ANOVA model.

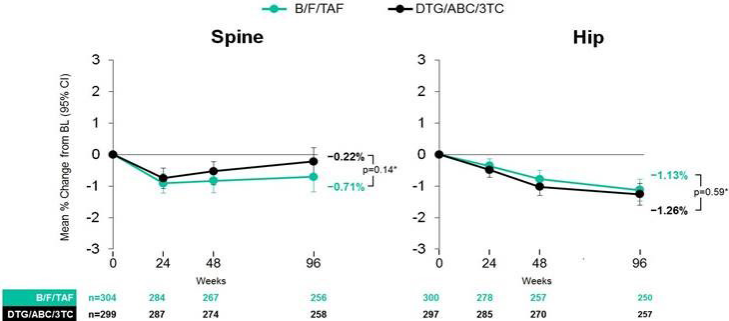

## P067

### HPV‐related malignancies screening to strengthen persistence in pre‐exposure prophylaxis among MSM and transgender women in the Dominican Republic

**R Paulino‐Ramirez, L Tapia, E Sanchez, A Benitez and A Mariño**

Instituto de Medicina Tropical & Salud Global, Universidad Iberoamericana, Santo Domingo, Dominican Republic

**Background**

Many studies have been exploring interactions of human papillomaviruses (HPV) among MSM and transgender populations from the Dominican Republic [1‐4]. These studies have reported persistently high prevalence of HPV high risk and low risk genotypes [2]. Pre‐exposure prophylaxis (PrEP) is a new preventive strategy to reduce HIV infection that has been evaluated throughout many countries, and more recently, in the DR. As part of this intervention, we integrated a comprehensive service to increase persistence in care, including: psychological support, anal health, hormonal and drug abuse counselling, and male health. The aim of this study was to evaluate the acceptability of anal smears for cytological evaluation, and persistence in PrEP during an implementation pilot.

**Materials and methods**

Anal pap smears were offered to all PrEP users during follow‐up visits to a primary care unit in Santo Domingo, DR. Smears were collected and placed in liquid‐cytology medium (ThinPrep®) for microscopic evaluation. All results were classified using the Bethesda method from ASCUS, to low and high squamous intraepithelial lesion (LSIL‐HSIL), or carcinoma *in situ*. Laboratory results were discussed with users, and educational materials developed to increase awareness of HPV infection in males and transwomen. Persistence rates were assessed before and after services were provided.

**Results**

PrEP services were provided to a total of 149 MSM and transwomen. Pap smear was offered at the third visit to the centre together with counselling and education about the importance of HPV early detection. Persistence in care was 93.1% after 120 days of PrEP start. Anal pap smears acceptability was 63.8% (Table 1). Of those, 6.3% were positive for HPV‐related cytological modifications, and 66.7% were diagnosed with LSIL.

**Abstract P067‐Table 1.**

**Table:** 

	Anal Pap Smears, n (%)
Anal pap smear acceptability	
Total anal pap smears offered	149 (100)
Pap smears realised	95 (63.76)
Pap smears results	
Negative evidence for HPV	86 (90.5)
Positive evidence for HPV	6 (6.3)
Description of positive anal pap results	
LSIL	4 (66.7)
ASC‐US	2 (33.3)

**Conclusions**

PrEP scaling‐up among key populations requires innovation and integrated services. More efforts are needed to increase awareness of HPV‐related malignancies among most‐at‐risk populations, and early screening should be offered as a package of care not only to people living with HIV, but their partners, and transwomen.

**References**

1. Paulino R, Tejada JC, Peña N, Garrido L. Atypical high‐risk human papillomavirus genotypes detection in anal versus cervical cytopathological modifications in an HIV (+) women cohort, Dominican Republic. HPV. 2014;CS.PP02.29.

2. Paulino R, Payano M, Pena N, Tejada. Comparison of Cervical and Anal Cytopathological Modifications in HIV (+) Female Individuals, a Hidden Epidemic. 28th International Papillomavirus Conference, San Juan, Puerto Rico, 2012.

3. Paulino R, Garrido LE, Fernandez A, Gomez N, Tejada JC. High‐risk HPV Genotypes and Anal and Cervical Dysplasia among HIV (+) Women, Dominican Republic. J Int AIDS Soc. 2014;17 Suppl 1.

4. Richards SD, Stonbraker S, Halpern M, Amesty S. Cervical cancer screening among transactional female sex workers in the Dominican Republic. Int J STD AIDS.

## P068

### First‐line INSTI use in Latin America: increasing, but still not enough. Observed trends for 2013 to 2017 from the HIV Latin American Workshop Group

**M Hojman^1^, L Calanni^2^, P Zitko^3^, S Sabato^4^, R Cuini^1^, P Parenti^5^, C Miglioranza^6^, R Teran^7^, J Contarelli^8^ and C Beltran^3^**

^1^Infectious Diseases, Hospital, Buenos Aires, Argentina. ^2^Infectious Diseases, CEIN, Neuquén, Argentina. ^3^Infectious Diseases, Complejo Asistencial Barros Luco, Santiago, Chile. ^4^Infectious Diseases, FUNCEI, Buenos Aires, Argentina. ^5^Infectious Diseases, Hospital Provincial del Centenario, Rosario, Argentina. ^6^Infectious Diseases, Hospital Interzonal de Agudos, Mar del Plata, Argentina. ^7^Infectious Diseases, Hospital, Quito, Ecuador. ^8^Infectious DIseases, Hospital Español, La Plata, Argentina

**Background**

Since the advent of potent combination therapy, HIV transmission and associated morbidity and mortality were reduced. The aim of this study is to describe characteristics related to ART in a time series of HIV patients of centres participating in TLA‐VIH (The Latin American Workshop Group).

**Material and methods**

An ecological analysis was performed using collected data from 29 centres of 10 Latin American countries. Data were organised by strata of age and sex (12 or 6 depending on the analysis), 4 strata of backbone, 4 by third drug and 5 corresponding to the year of admission to each centre (2013 to 2017). Contingency tables were used to observe trends as well as logistic models including three level random effect (strata, centre and country). ART use and covariables association was explored reporting odds ratio (OR). Sensibility analysis was carried out considering weights in accordance to the expected people living with HIV under control in each country.

**Results**

Data from 60,904 patients were obtained. During the study, 35,168 were newly diagnosed. Patient's distribution was similar between public and private centres. Genotypic‐resistance test (GRT) availability for naive patients was 55.2%. In 2017, 95.6% of patients under control were on ART. The least‐treated age stratum was 15 to 29 years, mainly males (*p* < 0.05). During 2013 to 2017, ART initiation at the year of enrolment increased in all countries (77.1% vs. 86.6%, *p* < 0.05) except in Chile, being higher in private centres (OR 2.28, 1.31 to 3.99). Tenofovir based regimens were the most used backbone and increased during years 2013 to 2017 (54.3% vs. 75.7%), AZT first‐line use decreased (28.7% vs. 4.1%) and abacavir remained stable (14.0% vs. 17.0%). Zidovudine replacement for tenofovir was observed in Colombia, Peru, Chile, Argentina and Costa Rica during the study, remaining countries had abandoned zidovudine before 2013. Efavirenz was the most frequent third drug used in first‐line, with a slight decrease (66.7% vs. 60.0%), followed by a boosted‐PI: 21.8% vs. 20.1%. Raltegravir was used in <5% of patients with a slight increase over time. Dolutegravir and darunavir/ritonavir were included in “others,” with <15.0% of use (Figure 1).

**Conclusions**

Current rate of patients on treatment is over the UNAIDS targeted 90%. There was a delay in ART initiation during the first year in care in <30 year old males. Targeted policies to this population should be urgently developed and implemented in our region. High use of efavirenz and low use of INSTI still persist despite international guidelines. Adequate measurement of cost‐effectiveness of certain drug use, GRT availability for naive patients and continue medical education are mandatory.

Abstract P068‐Figure 1. Percentage of type of backbone and third drug and used as first ART according to the year of admission (n = 35,168, n = 29 centres, 10 countries).

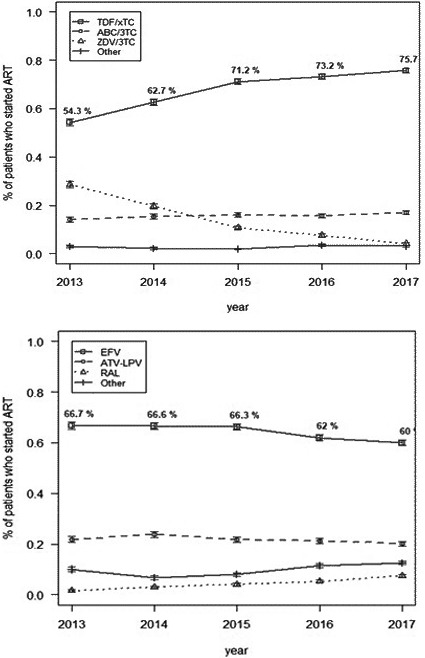

## P069

### Increasing transmitted drug resistance (TDR) to NNRTIs in Suriname

**M MacDonald‐Ottevanger^1^, K Brinkman^2^, G Culbaard^3^, J Roosblad^4^, S Harkisoen^3^, M Holtuin^5^, M van Eer^6^, K Waldring^7^, J Codrington^4^, L Woittiez^6^ and S Vreden^3^**

^1^Research Center, Academic Hospital, Paramaribo, Suriname. ^2^Department of Infectious Diseases, Onze Lieve Vrouwe Gasthuis, Amsterdam, Netherlands. ^3^Internal Medicine and Infectious Diseases, Academic Hospital, Paramaribo, Suriname. ^4^Clinical Laboratory, Academic Hospital, Paramaribo, Suriname. ^5^National AIDS Program, Ministry of Health, Paramaribo, Suriname. ^6^Internal Medicine and Infectious Diseases, Diakonessenhuis, Paramaribo, Suriname. ^7^Department of Internal Medicine, St. Vincentius Ziekenhuis, Paramaribo, Suriname

**Background**

HIV transmitted drug resistance (HIV‐TDR) has been increasingly observed in resource‐challenged countries and should therefore be monitored regularly. An HIV‐TDR prevalence >10% indicates a switch in first‐line antiretroviral therapy according to the World Health Organization (WHO) [1]. Suriname, a middle‐income country in South America, with an HIV prevalence of 1% to 2%, implemented universal access to combination antiretroviral therapy (cART) since 2005, and prescribes first and second‐line cART based on WHO guidelines. In 2009 the last TDR surveillance in Suriname showed a 1.0% occurrence. Since 2015, Suriname has implemented the HIV “test and treat” policy. Here, we present a recent evaluation of HIV‐TDR in Suriname.

**Materials and methods**

Treatment‐naive patients (≥18 years) presenting to care at the largest HIV clinic in Suriname were included during the period July 2015 to December 2016. Age, sex, CD4 and viral load (VL) at time of inclusion were registered. Genotyping was done on dried plasma spots at the Laboratory of the Public Health Agency of Canada by PCR on *pol* gene to cover all major protease and reverse transcriptase mutations for TDR. Drug resistance interpretation was done using the Stanford HIVdb (Version 8.4).

**Results**

In total, 113 patients were included. 58.4% (66/113) were male and the median age was 37.2 (IQR 29.7 to 47.2 years). 77.0% were late presenters (CD4 < 350, IQR 52.0 to 348.5); 61.1% with advanced HIV disease (CD4 < 200). The median VL at time of inclusion was 49000 copies/mL (IQR 5750–210000). Technically successful genotyping could be performed in 75 (66.4%). NNRTI mutations included non‐polymorphic mutations K103N(S) (4.0%) and polymorphic accessory mutations E138 (5.3%) and V179(V)D (2.6%). Most common NRTI mutation was K219Q (1.3%). Major drug mutations showed an overall TDR of 5.3%; NNRTI: 4.0% and NRTI: 1.3%, and no major PI resistance.

**Conclusions**

HIV transmitted drug resistance in Suriname increased from 1.0% in 2009 to an intermediate 5.3% in 2016, in particular NNRTI resistance. Although E138A was the most commonly found mutation, rilpivirine is not prescribed in Suriname. It is however, standard first‐line therapy in bordering French Guyana; important transborder migration has been documented. Adjustment of the first‐line therapy with NNRTI is not yet necessary according to WHO guidelines; careful monitoring of patients starting on NNRTI to prevent further spreading is however. HIV‐TDR testing should be repeated more frequently.

**Reference**

1. WHO. Guidelines on the public health response to pretreatment HIV drug resistance. 2017. [cited 2018 May 1]. Available from: http://apps.who.int/iris/bitstream/10665/255880/1/9789241550055‐eng.pdf?ua=1.

## P070

### Feasibility of the implementation of a hepatitis B vaccination programme in men who have sex with men and transgender women in Lima, Peru

**E Montalban Sandoval^1^ and J Lama Valdivia^2^**

^1^Infectious Disease, Impacta Peru, Lima, Peru. ^2^Direction, Impacta Peru, Lima, Peru

**Background**

Despite it can be prevented by currently available safe and effective vaccine, hepatitis B infection is still a major health problem which affects approximately 257 million people around the world and, in 2015, caused 887000 deaths [1]. This could be explained by low immunisation coverages and lack of vaccination programmes for population at risk. According to many investigations among hard to reach populations, a better acceptability would improve vaccination coverage when prevention programmes are included in clinics specialised in sexually transmitted infections (STD) [2‐4]. In Peru, core hepatitis B antibody seroprevalence has been estimated in 22.3% among men who have sex with men (MSM), in contrast among general population reaches 5% [5]. We think that prevention and vaccination programmes tailored for MSM and transgender women (TW) should be implemented in Peru. Our study explored the feasibility of implementing a hepatitis B vaccination programme in MSM and TW in Lima, Peru.

**Materials and methods**

Cross‐sectional study among MSM and TW who attend an STD clinic in Lima, Peru. A survey was used to measure 06 items: perception of barriers, benefits, and compliance to hepatitis B vaccination, communication with the primary care physician, severity and susceptibility to hepatitis B infection. Associated factors were identified through multivariate analysis.

**Results**

In total, 523 MSM, among them 103 TW, were recruited. The median age was 26 years; 87.1% perceived benefits; 85.7% would be compliant; 89.2% considered hepatitis B infection as serious; 42% could not get the vaccine; 68.8% did not have a primary care physician; and 53.5% felt they are not are risk. Receptive sexual role was associated to vaccination barriers (OR 1.88), low compliance (OR 1.78), and perceived poor vaccination benefits (OR 1.78). Age <25 years (OR 1.6) and history of sex on drugs (OR 1.56) were associated with difficulties in communicating with the primary care physician. History of sex under the effects of alcohol (OR 1.68) was a factor for perception of no vaccination benefits. Insertive sexual role was associated (OR 1.57) to perception that hepatitis B is not severe. Self‐definition as heterosexual was a significant factor (OR 3.16) for perceiving them at no risk to be infected. Education, self‐definition as sexual worker, condom use with MSM, and exchanging sex for money were not associated.

**Conclusions**

It is feasible to implement an optimised hepatitis B vaccination programme for MSM and TW in Peru. However, several identified factors threaten the success of such a programme.

**References**

1. Hepatitis B. WHO 2018.

2. Gunn RA, et al. Hepatitis B vaccination of men who have sex with men attending an urban STD clinic: impact of ongoing vaccination program, 1998‐2003. Sex Transm Dis. 2007;34(9):663–8.

3. McMillan A. Hepatitis B vaccination of men who have sex with men: experience with accelerated course of vaccination in a genitourinary medicine clinic. Int J STD AIDS. 2005;16(9):633–5.

4. Rudy ET, et al. Factors affecting hepatitis vaccination refusal at a sexually transmitted disease clinic among men who have sex with men. Sex Transm Dis. 2003;30(5):411–8.

5. Lama JR, et al. Hepatitis B infection and association with other sexually transmitted infections among men who have sex with men in Peru. Am J Trop Med Hyg. 2010;83(1):194–200.

## P071

### Experience with dolutegravir in real‐life in an HIV centre in Bogotá, Colombia

**H Paez Ardila^1^, A Cerezo^2^, F Barrientos^2^ and O Sussman^1^**

^1^Infectious Diseases, Asistencia Cientiica de Alta Complejidad, Bogotá, Colombia. ^2^Physician, Asistencia Cientiica de Alta Complejidad, Bogotá, Colombia

**Background**

Integrase inhibitors (II) are robust drugs in HIV treatment. We want to show the real‐life experience of patients receiving dolutegravir as a third drug in the treatment of HIV patients in an HIV centre in Colombia. We focus on outcomes and side‐effects.

**Materials and methods**

We did a retrospective descriptive study of HIV patients with dolutegravir treatment, in an HIV centre that attends to approximately 8000 patients in Colombia. We focus on outcomes and side‐effects of dolutegravir therapy.

**Results**

Data from 321 patients with dolutegravir treatment were selected. The exclusion criteria were: a) to have started dolutegravir after July 2018 because it was not possible to evaluate the viral load after six months, and b) to have incomplete data. A total of 182 were excluded because dolutegravir treatment was started between July to December 2018. For the final analysis, we included 139 patients with dolutegravir treatment. The backbone of patients who received dolutegravir was, 51% tenofovir/emtricitabine, 35% abacavir/lamivudina, 14% without nucleoside/nucleotide and 0% zidovudine/lamivudine. The indications to use dolutegravir were 39% (simplification integrase non‐experimented), 23% (simplification integrase experimented), 21.5% (rescue integrase non‐experimented), 10.8% (naïve) and 5.7% (rescue integrase experimented). After six months of dolutegravir treatment 94.2% had viral load <200 copies/mL, the other percentage had more than 200 copies/mL of viral load, associated with non‐adherence (Figure 1). A few side‐effects were presented; non‐hypersensitivity or anxiety was reported, only 1 patient presented depression, 5 patients presented aminotransferases elevation but 3 were associated with fatty liver disease and 1 with hepatitis B, creatinine elevation (average increase 0.27 mg/dL) was presented in 33% of patients and 5% of the patients presented creatinine decreased after dolutegravir treatment.

**Conclusions**

The switch to dolutegravir in any indication leads to excellent clinical outcomes and with a low rate of adverse effects in patients with HIV in Colombia.

Abstract P071‐Figure 1. Viral load control after six months.

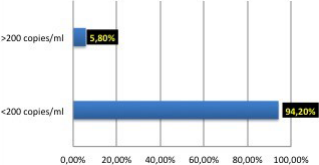

## P072

### Experience, attitudes and knowledge regarding dual antiretroviral regimens among American HIV providers

**J Gonzales Zamora, Z Henry and M Alcaide**

Division of Infectious Diseases, University of Miami, Miller School of Medicine, Medicine, Miami, FL, USA

**Background**

Three‐drug therapy has been the standard of HIV treatment; however, dual regimens have emerged as alternatives in patients with suppressed HIV viral loads [1,2]. Despite the growing data about dual regimens, there is still a lot of debate at the provider level regarding their use and effectiveness [3].

**Material and methods**

An online survey was administered to a convenience sample of HIV providers in the USA from October to December 2018. Our aim was to assess experience, knowledge and attitudes regarding dual antiretroviral regimens. Knowledge was evaluated by six questions (1 point for each correct answer). High knowledge was defined by a score of ≥5 points. We evaluated the association between knowledge and selected characteristics through chi‐square. Data was analysed in SPSS 22, New York, USA.

**Results**

We distributed 55 surveys and received 46 (83%) responses. The majority of the respondents were from academic institutions (45.7%) and private clinics (37.0%). The male to female ratio was 2.5/1. The providers were 31 to 60 years predominantly (84.8%). Most of them (43.4%) had 5 to 14 years of experience, and worked in outpatient settings (65.2%). Respondents were mainly M.D. Infectious disease specialists (45.7%) and nurse practitioners (23.9%). In terms of experience with dual regimens, 84.8% of providers admitted prior prescription of these regimens. Rilpivirine/dolutegravir was the most prescribed combination (76.09%). Dolutegravir+darunavir/ritonavir and dolutegravir+lamivudine were prescribed by 37% and 10.9% of providers respectively. Most providers (69.6%) stated that they inform their patients about the possibility of using dual regimens. Regarding knowledge, 45.7% of respondents self‐reported good knowledge. High knowledge score was obtained by 60.9% of providers. In comparison with the rest of the study cohort, providers with high knowledge worked more in outpatient settings (50.0% vs. 16.7%, *p* < 0.022), and treated a higher number of patients (>100 patients monthly, 39.3% vs. 5.6%, *p* < 0.011). In terms of attitudes, 69.6% of providers would consider prescribing dual regimens in patients who are fully compliant, and 60.9% would prescribe them in patients with undetectable viral load for >6 months. A high rate of providers (73.9%) felt uncomfortable prescribing drugs for new indications, 58.7% expressed concern about the effectiveness of dual regimens, and 65.2% believed that 3‐drug regimens were better than dual regimens.

**Conclusions**

Our study revealed high knowledge and vast experience on the use of dual antiretroviral regimens among American HIV providers. Despite positive attitudes towards dual regimens, providers still have significant concerns about their effectiveness.

**References**

1. Boswell R, Foisy MM, Hughes CA. Dolutegravir Dual Therapy as Maintenance Treatment in HIV‐Infected Patients: A Review. Ann Pharmacother. 2018;52(7):681–89.

2. Lee SA, Kim SW, Chang HH, et al. Effectiveness, Safety, and Tolerability of a Switch to Dual Therapy with Dolutegravir Plus Cobicistat‐Boosted Darunavir in Treatment‐Experienced Patients with Human Immunodeficiency Virus. Infect Chemother. 2018;50(3):252–62.

3. Höring S, Löffler B, Pletz MW, et al. Dual antiretroviral therapy with tenofovir (TDF) and darunavir/ritonavir (DRV/RTV) in an HIV‐1 positive patient: a case report, review, and meta‐analysis of the literature on dual treatment strategies using protease inhibitors in combination with an NRTI. Infection. 2018;46(5):599–605.

## P073

### Six years’ experience in the use of raltegravir as part of regimens for treatment of HIV‐infected patients in a Peruvian reference hospital of the social security

**E Contreras Calero^1^, F Mendo Urbina^1^, R Pichardo Rodriguez^2^ and M Saavedra Velasco^1^**

^1^Hospital Nacional Edgardo Rebagliati Martins, Infectious Diseases Unit, Lima, Peru. ^2^Universidad Ricardo Palma, Faculty of Medicine, Lima, Peru

**Background**

Since 2007, integrase inhibitors (INSTI) were approved for treatment of HIV‐infected patients [1]. Raltegravir was the first licensed INSTI [1,2]. In Peru, regimens of antiretroviral therapy including INSTIs are recommended in the national guideline for treatment of HIV‐infected patients, but its use is limited [3]. Raltegravir is the only INSTI available within the social security in Peru. However, clinical data is also limited in our country. The objective was evaluating the efficacy and security in real clinical condition of the use of Raltegravir as part of regimens for treatment of HIV‐infected patients in a Peruvian reference hospital of social security.

**Materials and methods**

An observational retrospective study in a population of patients receiving antiretroviral treatment in the infectology‐unit of Hospital Nacional Edgardo Rebagliati Martins‐ESSALUD, between 2012 and 2018 was conducted. We included all patients who started using raltegravir and had at least two measurements of HIV viral load and laboratory tests before and after (24 weeks) initiating treatment. Data were collected, using a standardised case report form from clinical records. The efficacy was defined as the viral load negative in a minimum period of 24 weeks after receiving raltegravir and security as the absence of adverse events or need to discontinue treatment. The viral load was determined by the PCR method in HIV Architec team (Roche®). CD4 levels were measured by flow cytometry in the BDFACS Canto II equipment. The haemogram and the biochemical determinations were processed in the Sysmex 2000 and Advia 1800 equipment respectively. Media and standard deviation were used for quantitative variables and frequencies and percentage for qualitative variables. Data was analysed in SPSS V.22.

**Results**

A total of 157 patients were included in the analysis. Age range more frequent was from 45 to 55 years (25.4%; n = 40) and proportion of male was 76% (n = 119). A 10 % (n = 16) of patients were naive with median baseline CD4 counts of 100 cells/μL and whom were on antiretroviral treatment, had HIV viral load median >400 copies/mL. More frequent indications were: virologic failure (n = 102) and toxicity (n = 33). Virological suppression was achieved in 92.4% (n = 143) at week 24 and adverse effects presented in 10% of them (Table1). More frequent commodities were arterial hypertension (23.73 %) and diabetes mellitus (16.95%) and one patient coinfected with hepatitis C. There were no discontinuations of raltegravir due to adverse effects.

**Conclusions**

Use of raltegravir as part of regimens for treatment of HIV‐infected patients appears safe and effective.

**Abstract P073‐Table 1. Effect of raltegravir after initiation of treatment at 24 weeks**

**Table:** 

	At entry, n (%)	At 24 weeks, n (%)
Load viral		
Non detectable	23 (14.65)	145 (92.36)
>400 copies/mL	134 (85.35)	12 (7.64)
CD4 level		
<200 cells/μL	66 (42.04)	32 (20.38)
200 to 500 cells/μL	56 (35.67)	63 (40.13)
>500 cells/μL	35 (22.29)	62 (39.49)
Haemogram and serum biochemistry		
Haemoglobin		
Males (<13 mg/dL)	40 (25.48)	13 (8.28)
Females (<12 mg/dL)	15 (9.55)	9 (5.73)
Neutropenia (absolute neutrophils)		
Mild (1) 1.5 × 10^3^/μL)	17 (10.83)	4 (2.55)
Moderate (0.5 to 1 × 10^3^/μL)	6 (3.82)	0 (0.00)
Severe (<0.5 × 10^3^/μL)	1 (0.64)	2 (1.27)
Absolute platelets		
<150 × 10^3^/μL	10 (6.37)	1 (0.64)
Glucose		
>106 mg/dL	16 (10.19)	26 (16.56)
Creatinine		
Males (>1.1 mg/dL)	9 (5.73)	13 (8.28)
Females (>0.8 mg/dL)	7 (4.46)	5 (3.18)
Hepatic profile		
AST > 34 μL	40 (25.48)	28 (17.83)
ALT > 49 μL	29 (18.47)	23 (14.65)
AP > 129 μL	48 (30.57)	40 (25.48)
TB > 1.2 mg/dL	9 (5.73)	7 (4.46)
DB > 0.3 mg/dL	8 (5.10)	13 (8.28)
IB > 0.5 mg/dL	15 (9.55)	12 (7.64)
Lipidic profile		
Cholesterol > 200 mg/dL	38 (24.20)	43 (27.39)
HDL‐C < 60 mg/dL	127 (80.89)	113 (71.97)
LDL‐C > 100 mg/dL	68 (43.31)	71 (45.22)
Cholesterol/HDL‐C >5	42 (26.75)	49 (31.21)
Triglycerides >250 mg/dL	34 (21.66)	34 (21.66)

ALT, alanine aminotransferase; AP, alkaline phosphatase; AST, aspartate aminotransferase; DB, direct bilirubin; HDL‐C, high‐density lipoprotein; IB, indirect bilirubin; LDL‐C, low‐density lipoprotein; TB, total bilirubin.

**References**

1. Mouscadet J‐F, Tchertanov L. Raltegravir: molecular basis of its mechanism of action. Eur J Med Res. 2009;14 Suppl 3:5–16.

2. Gatell JM. Eficacia del raltegravir: desde los voluntarios sanos a la fase III. Enfermedades Infecc Microbiol Clínica. 2008; 26:29–33.

3. DIGEMID. Technical Report No 09‐2011. Raltegravir 2011. Report No. 9.

## Viral Hepatitis

## P074

### Detection of antibodies anti‐ARFp as biomarker of liver fibrosis progression in patients with chronic HCV

**S Lima de Souza^1,2^, L Liebscher Vidal^3^, R de Mello Perez^1^, J de Araujo Neto^1,2^, H Moraes Coelho^1,4^, M Alves Soares^1,2^ and A Felipe Santos^1^**

^1^Federal University of Rio de Janeiro (UFRJ), Rio de Janeiro, Brazil. ^2^National Institute of Cancer (INCA), Rio de Janeiro, Brazil. ^3^Farmanguinhos ‐ Oswaldo Cruz Foundation, Rio de Janeiro, Brazil. ^4^D'Or Institute for Research and Education (IDOR), Rio de Janeiro, Brazil

**Background**

It is estimated that 71 million people are infected with Hepatitis C virus (HCV) and per year 400,000 people die from diseases related to HCV infection. In chronic hepatitis C, there is progression of liver fibrosis, which can develop to cirrhosis and hepatocellular carcinoma (HCC). Fibrosis can be classified in a five level severity scale, from F0, without fibrosis, to F4, which is cirrhosis. Although available treatments have high cure rates, HCC can develop even after clearance of infection. Alternative Reading Frame protein (ARFp) is a viral protein synthesised by an alternative reading frame −2/+1 in the region encoding the core. This protein is expressed during natural HCV infections and is recognised by serum antibodies. Recently, some of its mechanisms in carcinogenesis were elucidated, such as cell cycle acceleration and increased of tumour foci. Previous studies have shown a higher prevalence of anti‐ARFp antibodies in patients with HCC compared to patients without HCC but did not consider the different stages of fibrosis within the non‐HCC group. This study aims to detect anti‐ARFp antibodies in HCV positive patients with different levels of fibrosis as a biomarker of progression of hepatic disease.

**Materials and methods**

Serum of 279 patients were collected, of which 4 were F0, 74 F1, 54 F2, 51 F3, 83 F4 without HCC and 13 F4 with HCC. Plasmids containing the sequences of ARFp and core genotype 1a were used in this study. The plasmids were transformed into *Escherichiacoli* BL21 (DE3) to express the ARF and core proteins with IPTG. Proteins purification by affinity chromatography and they were used to perform the immunological assay ELISA. The core protein was used as a positive control of the assay.

**Results**

There was no difference in the proportions of anti‐ARFp antibodies between F1 and F2 groups (21% and 18% positive, respectively) and between F3 and F4 (39% and 42% positive respectively). Dividing patients into mild hepatic injury (F0 to F2) and advanced liver injury (F3 and F4), there was a significantly higher proportion of anti‐ARFp antibodies in the advanced disease group, with 41% positive, compared to 20% in the group with F0 to F2. Comparing cirrhotic patients with and without HCC, there was also a higher proportion of positive serology for ARFp in the HCC group, with 91% positive.

**Conclusion**

We conclude that the presence of anti‐ARFp antibody is a biological marker of progression of liver fibrosis in HCV positive patients.

## P075

### A sub‐analysis of Puerto Rican hepatitis C infected patients enrolled in phase 2 & 3 glecaprevir–pibrentasvir clinical programme

**G Sepulveda‐Arzola^1^, B Rosado‐Carrion^2^, S Lovell^3^, G Bugarin^4^, F Aponte^5^, E Crown^3^ and F Rodriguez‐Perez^6^**

^1^Infectious Disease, Instituto de Investigación Científica del Sur, Ponce, Puerto Rico. ^2^Gastroenterology, GHGCPR Research Institute, Ponce, Puerto Rico. ^3^AbbVie Global Medical Affairs, AbbVie. S.A., Chicago, IL, USA. ^4^AbbVie Medical Affairs, AbbVie. S.A., Buenos Aires, Argentina. ^5^AbbVie Medical Affairs, AbbVie Medical Affairs, San Juan, Puerto Rico. ^6^Gastroenterology, Klinical Investigations Group, San Juan, Puerto Rico

**Introduction**

Glecaprevir–pibrentasvir (G/P) is a next generation coformulated and oral antiviral pangenotypic compound containing a PI and N5A inhibitor against hepatitis C virus (HCV). G/P was licensed in the US and Europe in July/Aug 2017. Its approval was supported by data from phase 2/3 global clinical studies showing high efficacy rates and favourable safety profile.

**Objectives**

To describe clinical characteristics and virological response to G/P in Puerto Rican patients included in phase 2/3/3b studies.

**Materials and methods**

Sub‐analysis of Puerto Rican HCV‐infected patients included in a pool of 3233 patients from 14 clinical studies conducted in 32 countries. Patients were either mono‐/coinfected, treatment‐naïve or experienced and treated for 8, 12 or 16 weeks; primary endpoints: SVR12 and safety profile.

**Results**

Seventy‐seven patients from Puerto Rico: 52 were male (65.8%), mean age 54.2 years (29 to 78), mean BMI (kg/m^2^) 27.3 (≥30 in 17 patients), mean baseline HCV RNA 6.2 log 10 IU/mL, mean eGFR 84.7 mL/min. Genotype 1 prevalence 87.3% (n = 69), 49/69 GT1A (62%), GT2 n = 5 (6.3%), GT3 n = 4 (5.1%) GT4 n = 1 (1.3%). Treatment naïve 63 pts (79.7%), baseline fibrosis stage: F0‐F1 39 (53.4%), F2 3 (4.1%), F3 6 (8.2%), F4 25 (34.2%), missing 6 patients (7.5%). All patients included in this analysis had 100% SVR4/SV12. AEs occurred in 38 patients (48.1%); related to DAA 13 (16.5%), serious AE 4 (5.1%). No AE leading to discontinuation.

**Conclusions**

Compared to the published data of the whole integrated analysis, the Puerto Rican patients presented some differences in terms of genotype distribution (lower prevalence of GT3 infection) and high virologic response rates across all GT (100% vs. 98% global analysis). Puerto Rico sub‐analysis demonstrates comparable tolerability profile and 100% of efficacy. The small sample size could be a limitation, but the data still shows an interesting profile of the Puerto Rico experience in the treatment of chronic hepatitis C.

## P076

### Case–control study to identify risk features associated to anti‐HCV serology reagent in prisoners in the state of Paraná, Brazil

**T Marques^1^, L Ferreto de Almeida^2^, F Follador^1^, A Vieira^1^, R Yamada^3^, L Lucio^1^, J Titon^3^, R Torres^4^, G Amaral^5^ and H Coelho^6^**

^1^Postgraduate Program in Applied Health Sciences, State University of West Paraná, Francisco Beltrão, Brazil. ^2^Medicine & Postgraduate Program in Applied Health, State University of West Paraná, Francisco Beltrão, Brazil. ^3^Medicine, State University of West Paraná, Francisco Beltrão, Brazil. ^4^Department Penitentiary of Paraná, Security and Penitentiary Administration of Paraná, Curitiba, Brazil. ^5^Epidemiology, State Department of Health, Maringá, Brazil. ^6^Clinical, Toxicological & Bromatological, University of São Paulo, Ribeirão Preto, Brazil

**Background**

The prison system in Paraná, Brazil presents serious problems related to the increasing number of prisoners [1] and becomes more intense in the control of the hepatitis C virus (HCV) due to the fact that the incarcerated population is considered a high‐risk group for contagious diseases because of the favourable conditions found in prison for spreading these morbidities [2,3]. The objective of this study was to identify features associated with HCV infection among male prisoners in the prison system (correctional institutions) of Paraná, Brazil.

**Materials and methods**

This is a case–control study (27 cases and 54 controls) with men arrested in 11 penitentiaries in Paraná, Brazil, where the information was obtained through the application of a questionnaire in a cross‐sectional epidemiological survey for anti‐HCV infection in the period from May 2015 to December 2016. Eligible men were recruited after the positive result for anti‐HCV. The selection of the cases and controls considered the result of the serology by an enzyme‐linked immunosorbent assay, following the manufacturer's instructions, matched by age, location of the penitentiary and time in prison. Odds ratio (OR) and 95% confidence interval (CI) were estimated using binary logistic regression analysis to identify the predicting factors of the variable to be explained.

**Results**

The participants’ mean age was 39 years, and the prevalence was predominantly among individuals over 30 years of age. The logistic regression analysis showed that the main significant risk factor for the acquisition of HCV infection was the use of injectable drugs (OR = 4.00; 95% CI, 1.41 to 11.35; *p* < 0.001).

**Conclusions**

This is the first case–control study reported with male prisoners in the closed prison system of Paraná, Brazil. This study provides evidence that HCV infection is associated with drug use by this population. This information is pivotal for tailoring prevention programmes and guiding specific socioeducational measures that aim to reduce or prevent HCV transmission within the prison setting.

**References**

1. Brasil (2017). Ministério da Justiça e Segurança Pública Departamento Penitenciário Nacional. Levantamento Nacional de Informações Penitenciárias – INFOPEN. Brasilia, DF.

2. Puga MAM, Bandeira LM, Pompilio MA, Croda J, Rezende GRd, Dorisbor LFP, et al. Prevalence and Incidence of HCV Infection among Prisoners in Central Brazil. PLoS One. 2017;12(1):e0169195. Available from: https://doi.org/10.1371/journal.pone.0169195.

3. Belaunzarán‐Zamudio PF, Mosqueda‐Gomez JL, Macias‐Hernandez A, Sierra‐Madero JG, Ahmed S, Beyrer C. Risk factors for prevalent hepatitis C virus‐infection among inmates in a state prison system in Mexico. . PLoS One. 2017;12(6):e0179931. Available from: https://doi.org/10.1371/journal.pone.0179931.

## P077

### The opportune diagnosis of hepatitis C, an option to prevent vertical transmission and follow‐up on births of infected mothers

**E Silva Cabrera, A Sánchez Gutiérrez, T Licourt Otero and A Arteaga Yera**

Centro de Inmunoensayo, Programas Nacionales de Salud, La Habana, Cuba

**Background**

The hepatitis C virus (HCV) is a known cause of chronic liver disease in adults, but its importance in pregnant women and children is underestimated. It is thought that at present the main route of acquiring HCV in children is vertical transmission. However, it is very likely that more than half of infected infants are not diagnosed since mothers are not evaluated during pregnancy and newborns are asymptomatic after delivery. The objective of the work is to deepen the convenience of introducing the prenatal screening to HCV.

**Materials and methods**

Studies of the state‐of‐the‐art and the behaviour of the disease in Cuba were carried out.

**Results**

Screening in pregnant women or women of reproductive age with an interest in getting pregnant is not common practice in most countries; however, HCV transmission risk is estimated as 4% to 8% among mothers without HIV infection and as 17% to 25% among mothers with HIV infection. The new treatment regimens with direct acting antivirals increase the hope of cure of HCV infection. In Cuba, the National Programme does not include research on pregnant women. From 2012 to date, more than 100 new cases of HCV were reported annually, positivity in blood banks in 2017 was 1.1% and epidemiological surveillance was 3.5%. Cirrhosis and other chronic diseases of the liver ranked ninth in mortality in the country.

**Conclusions**

HCV positivity in blood donors and in epidemiological research, as well as mortality by cirrhosis and other liver diseases, suggest that hepatitis is not a solved issue in Cuba. So, we believe that opportune diagnosis of the infection and the new treatment regimens could be a realistic strategy to eradicate the vertical transmission of HCV in the future and reduce the consequences of a virus infection in young adults.

## Virology and Immunology

## P078

### Evidence of partial seroreversion after early initiation of antiretroviral treatment in an acute HIV infection cohort from Argentina

**G Turk^1^, V Pereyra^2^, C Piccardo^1^, M Figueroa^2^, A Gun^3^, P Cahn^2^, H Salomon^1^ and O Sued^2^**

^1^INBIRS, CONICET, Buenos Aires, Argentina. ^2^Research, Fundación Huésped, Buenos Aires, Argentina. ^3^Laboratory, Fundación Huésped, Buenos Aires, Argentina

**Background**

Initiating cART as early as possible following HIV infection to limit the size of the viral reservoir and improve disease prognosis has been widely recommended. However, some cases of seroreversion after very early antiretroviral therapy have been reported which has important implications for the safety of blood product and organ and tissue donation, among other settings. Here, we assessed the presence of HIV antibodies among individuals initiating cART during acute/early HIV infection in a cohort of Argentinean seroconverters.

**Methods**

Twenty‐four recently diagnosed subjects were enrolled as part of the Grupo Argentino de Seroconversion study group. Baseline samples were obtained at a median of 60 days post‐presumed date of infection. Regular diagnosis algorithm was performed to confirm infection. After subjects initiated cART, samples were obtained at six‐month intervals and up to 54 months’ post‐cART. Fourth‐generation ELISA, rapid tests (RT) and Western Blot (WBs) were performed.

**Results**

All 24 subjects included in this study had serologically confirmed HIV infection at baseline with detectable viral load. Post‐cART, all subjects showed reactive RTs and fourth‐generation ELISAs with elevated sample‐to‐positive ratios at all time points. In the WBs, different longitudinal patterns were observed: some individuals showed the same numbers of bands along time (compared to baseline); others showed an increasing number of bands while others showed a decreasing number of bands along time. It was observed that 50% of early‐treated subjects, that is those who started cART early (<120 days’ post‐infection), showed, compared to the baseline WB, less WB bands or the same number of bands but with decreasing intensity, than those who delayed the start of treatment after acquiring infection (>120 days post‐infection). Moreover, one subject turned from one positive WB at baseline (5 bands: gp160/gp120/gp41/p24/p17) to an indeterminate state by 24 months’ post‐cART (only the gp160/gp120 band). Within the same group, 22.2% showed the same and 27.8% showed an increasing number of bands. However, these proportions were 20%, 40% and 40%, respectively, in the delayed‐treated subjects.

**Conclusions**

Evidence of partial seroreversion was found in the studied cohort. The rate of reversion was higher within subjects who received cART at very early times post‐infection and might become even more frequent with more prolonged time on suppressive cART. This phenomena, in addition to the delayed seroconversion seen among individuals receiving PrEP and the seropositivity produced by new HIV vaccines among individuals without infection, might challenge our current HIV diagnosis algorithms. Further research is warranted in the context of Test and Treat strategy and Combined Prevention paradigm.

## P079

### Surveillance of HIV pretreatment resistance in Cuban patients according to WHO recommendations

**L Machado^1^, M Blanco^1^, H Díaz^2^, L López^1^, A Súarez^3^, T Rojas^3^, M Dubed^3^, N Váldes^4^, E Noa Romero^3^, L Martínez^2^, M Pérez^5^, J Joanes^6^, I Cancio^6^, D Romay^1^, C Rivero^1^, M Lantero^6^, M Rodríguez^1^ and G Ravasi^7^**

^1^Molecular Biology, AIDS Research Laboratory, Mayabeque, Cuba. ^2^Infectious Diseases, Hermanos Ameijeirias Hospital, Havana, Cuba. ^3^Virology, AIDS Research Laboratory, Mayabeque, Cuba. ^4^Diagnosis, AIDS Research Laboratory, Mayabeque, Cuba. ^5^Quality Assurance, AIDS Research Laboratory, Mayabeque, Cuba. ^6^HIV/AIDS, Ministry of Public Health, Havana, Cuba. ^7^HIV Hepatitis, TB & STI Unit, Pan American Health Organization, Washington, DC, USA

**Background**

The World Health Organization (WHO) recommends a method to estimate nationally representative pretreatment HIV drug resistance (PDR) in order to evaluate the effectiveness of first‐line treatments. The aim of this study was to determine the prevalence of PDR in Cuban adults infected with HIV‐1.

**Materials and methods**

A cross‐sectional study in Cuban adults infected with HIV‐1 over 18 years was conducted. The probability proportional to size method for the selection of municipalities and patients without a prior history of antiretroviral treatment during the period from January 2017 to June 2017 was used. The plasma from 141 patients from 15 municipalities for the determination of viral subtype and HIV drug resistance was collected. Some clinical and epidemiological variables were evaluated.

**Results**

80.9% of the patients corresponded to the male sex and 76.3% were men who were having sex with other men (MSM). The median CD4 count was 371 cells/mm^3^ and the median viral load was 68000 copies/mL. The predominant genetic variants were subtype B (26.9%), CRF19_cpx (24.1%), CRF 20, 23, 24_BG (23.4%) and CRF18_cpx (12%). Overall, the prevalence of PDR was 29.8% (95%, CI 22.3 to 38.1). The prevalence was 12.8% (95%, CI 6.07 to 16.9) for any nucleoside reverse transcriptase inhibitor (NRTI), 23.4% (95%, CI 16.7 to 31.3) for any non‐reverse transcriptase inhibitor (NNRTI) and 1.4% (95%, CI 0.17 to 5.03) for any protease inhibitor (PI). The most frequent mutations detected were K103N (12.9%), G190A (6.4%) and Y181C (4.8%).

**Conclusions**

The values above 10% in the prevalence of PDR to the NNRTI evidence the compromise of the first lie of antiretroviral therapy used in Cuba and the need to look for new treatment options that contribute to therapeutic success and compliance with global goals 90‐90‐90 listed by UNAIDS.

## P080

### Biological and molecular characterisation of HIV‐1 isolates from Cuban patients at two moments of their natural history

**E Noa Romero^1^, L Machado^2^, J Enriquez^1^, A Duran^1^, M Dubed^1^, M Blanco^2^, A Fraga^3^, A Suárez^1^, H Díaz^2^ and D Romay^2^**

^1^Virology, AIDS National Research Laboratory, San José de las Lajas, Cuba. ^2^Molecular Biology, AIDS National Research Laboratory, San José de las Lajas, Cuba. ^3^Immunodiagnostic, AIDS National Research Laboratory, San José de las Lajas, Cuba

**Background**

Variation in viral characteristics, host defence responses (likely explained by variation in host genetics), and environmental factors may all contribute to the variation in the natural course of HIV infection. In this work, we aimed to correlate the genotypic and phenotypic characteristics of strains of HIV‐1 isolated from Cuban patients with the natural history of the infection.

**Methods**

Sixteen patients without antiretroviral therapy (ART) were included. The first sample was taken in the eight months after the diagnostic and the second sample two years later. Peripheral blood with anticoagulant was used for biological and molecular characterisation. The viral subtype was determined by phylogenetic analysis of the protease‐RT region and the V3 loop of HIV‐1. Viral isolation and biological characterisation (syncytium induction, SI) was performed using the standardised technique of the WHO (2002) and the prediction of the use of coreceptors through the genotypic analysis of the V3 loop (rule 11/25).

**Results**

The viral subtypes identified were B (5 patients); CRF19_cpx (5); CRF 20, 23, 24_BG (4) and CRF18_cpx (2). At the beginning of the study, 11 patients were in clinical stage A1 and A2, the average CD4 count was 389.3 cells/mL (range 53 to 1260), the average viral load was 5.29 log, the virus was isolated from 10 patients (3 SI) and strains R5 predominated (8 patients). In the second sample, patients had evolved clinically; eight in clinical stages A3 to C3 and ten had started antiretroviral treatment, with an increase in the CD4 count at 443.5 cells/mL (range 205 to 792) and a decrease in viral load to 4.34 log. The virus was isolated from 10 patients (4 SI) and strains X4 and R5X4 predominated (12 patients). The patients with ART were related to the viral variant CRF19_cpx (5 patients); under mean CD4 cell count in the first sample versus in the naives and strains X4 and R5X4 predominated. The patients without ART had not evolved clinically, all at the beginning of the study had strains R5 and only in 2 had the virus been isolated. Of these, in the second sample, the virus was isolated from 5 patients; but only one was SI and strains R5 or R5X4 predominated.

**Conclusions**

The natural history of HIV infection in these Cuban patients was marked by immunological damage at the time of their serological diagnosis and the emergence of strains X4 and R5X4, that provide insight for the development of therapeutic or preventive intervention strategies.

## P081

### HIV‐1 integrase inhibitor resistance in Cuban patients during 2018

**L López Rizo^1^, L Machado Zaldivar^1^, M Blanco de Armas^1^, D Romay Franchi^1^, B Rivero Martínez^1^, N Valdes de Calzadilla^2^, H Díaz Torres^3^ and R Rodriguez Feo‐Cou^4^**

^1^Molecular Biology, AIDS Research Laboratory, Mayabeque, Cuba. ^2^Diagnostic, AIDS Research Laboratory, Mayabeque, Cuba. ^3^Infectious Diseases, Hermanos Ameijeiras Hospital, Havana, Cuba. ^4^Epidemiology, MINSAP, Mayabeque, Cuba

**Background**

High levels of HIV resistance to non‐reverse transcriptase inhibitors (NNRTI) have led to the use of integrase inhibitors (INSTI) in the first line of treatment. Currently, in Cuba, a group of patients with multiple therapeutic failures and multiresistance to highly active antiretroviral therapy (HAART) receive treatment with INSTI. Therefore, it would be beneficial to know the resistance behaviour to these new drugs, due to their potential to be used in the first line of ART in our country. The aim of this study is to evaluate resistance to INSTI in a group of treated and untreated Cuban patients.

**Materials and methods**

A prospective study was conducted in 59 HIV‐1 infected individuals (6 were untreated and 38 received HAART) during September to December 2018. The viral RNA for the genotyping of the HIV‐1 integrase region was isolated. The HIV subtype was determined by phylogenetic analysis. The profile of mutations associated with INSTI resistance was also determined. Some clinical and epidemiological variables were evaluated.

**Results**

The genotypic analysis was performed on 44 of the 59 patients. The 88.1% of the samples studied were male. The predominant subtypes were: CFR20, 24_BG (30%), CRF18_cpx (26%), CRF19_cpx (24%), B (17%) and A (2%). All untreated patients were susceptible to INSTI. The 8% of the treated patients presented mutations associated with resistance to INSTI, which were detected in subtypes B and CRF24_BG. The most frequent mutations were: T97A (5.3%), G143S (2.6%) and Q148H (2.6%). The 8% of treated patients had low and intermediate levels of resistance to elvitegravir and raltegravir, and 2.6% showed high resistance to dolutegravir and bictegravir.

**Conclusions**

The low levels of HIV resistance to INSTI in treated Cuban patients in this study demonstrate the need for a constant surveillance and monitoring of resistance to these drugs.

## P082

### Pharmacological resistance profile and the most frequent mutations in IGSS patients

**G Rios^1^, L Godínez^1^ and R López^2^**

^1^Internal Medicine, Instituto Guatemalteco de Seguridad Social, Guatemala City, Guatemala. ^2^Infectology, Instituto Guatemalteco de Seguridad Social, Guatemala City, Guatemala

**Background**

We studied 286 patients with virological failure to antiretroviral treatment (ART) of the first‐line admitted to the infectology service of the Hospital General de Enfermedades del Instituto Guatemalteco de Seguridad Social (IGSS) from 2015 to 2017. All patients who underwent a viral genotype, with virological failure to the first‐line treatment and those who were discarded bad adherence were included in the study. The objective was to determine the pharmacological resistance profile and the most frequent mutations in IGSS patients.

**Methodology**

An observational, retrospective and transversal study was performed. Mutations are reported in frequencies and percentages, the platform hivdb.stanford.edu/hivdb/by‐mutations/was used for the interpretation of mutations and they were reported in frequencies and percentages.

**Results**

Combined resistance for non‐nucleotide reverse transcriptase inhibitors (NNRTIs) and nucleoside reverse transcriptase inhibitors (NRTIs) accounted for 25% of cases, the combination of NNRTIs and NRTIs and protease inhibitors (PIs) 9.1%, followed by the NNRTIs 10%, the NRTIs 6% and finally for the IP 5%. The most frequent mutations for NNRTIs were K103N, L100I, V108I, G190A and E138G that confers high level of resistance to efavirenz (EFV) nevirapine (NVP), intermediate to etravirine (ETR). The most frequent mutations for NRTIs were M184V/I , T215Y, Y115F and K65R conferring high level of resistance to lamivudine (3TC), emtricitabine (FTC), abacavir (ABC), tenofovir (TDF) and for PIs The V11I, I54V, L76V and M46I mutation that confers high level of resistance to atazanavir/ritonavir (ATV/r), lopinavir/ritonavir (LPV/r), darunavir/ritonavir (DRV/r).

**Conclusions**

To our knowledge, this is the largest report of mutations performed in Guatemala which provides valuable information on the behaviour of HIV‐1 in our population. The combined mutation to NNRTIs and NRTIs was the most common, which occurred in 1 in 4 patients. Mutations to NNRTIs and NRTIs together with PIs were the third most frequent.

